# Supplementary material for: Association of cataract and sun exposure in geographically diverse populations of India: The CASE study. First Report of the ICMR-EYE SEE Study Group
Source: PLoS One. 2020 Jan 23;15(1):e0227868. doi: 10.1371/journal.pone.0227868 (PMC6977762; doi:10.1371/journal.pone.0227868)
Supplement: S1 File — The Hindi and English versions of the questionnaires used in the study. (PDF) [file pone.0227868.s001.pdf]

<u>Participant Information Sheet</u>

Title of project: - Multi-centric Collaborative Study on the impact of Environment Changes and Ultra Violet Radiation(UVR) exposure on ocular health in India

Dr. Rajendra Prasad Centre For Ophthalmic Sciences, All India Institute of Medical Sciences, New Delhi is conducting a study related to Ultra Violet Radiation and eye diseases. Environment Changes and Ultra Violet Radiation has been the overriding physical environmental concern for the past decade as it significantly affects the ocular health. The potential effects on ocular health of long-term climatic change include allergic diseases, diseases of the conjunctiva, cornea, lens and retina. The main concern of the study is UV related ocular damage. Cataract is the most common cause of blindness in India. In this study we would like to know the correlation of UV Radiation and prevalence of eye disease like cataract, dry eye, Pterygium and spring catarrh (VKC). This will help us in preventing the most serious problem of eyes in the community.

To get the above information, we will seek participation from children in the age group of 5 to 15 for V.K.C. and people above 40 years of age for cataract, dry eye and pterygium in the population. A questionnaire will be administered to you. We shall examine your eyes thoroughly at Dr. R. P. Centre of Ophthalmic Sciences, AIIMS in accordance to the normal standard of care. Investigations on the eye will include dilating your pupil by instilling some eye drops. This will lead to harmless blurring of vision for 1-2 days. It will subside by itself. Photographs of the lens of your eyes will be taken by special cameras. All of the above examinations and tests shall be completed in one visit of approximately 3 hours.

All the information that you provide and your investigation results shall be kept confidential. There is no risk involved to you in this study.
You are free to participate or withdraw from this research study at any time. You will be provided free treatment for your eye problem. Your decision to participate or withdraw will not affect your treatment in anyway.

In case of any further information or clarification at any time, you are requested to contact the following:

Prof. Radhika Tandon, Professor **26593145**

Dr. Praveen Vashist, Associate Professor **9868398410, 26593143**

Participant Informed Consent Form

Participant identification number for this trial: ________________________

Title of project: - Multi-centric Collaborative Study on the impact of Environment Changes and Ultra Violet Radiation (UVR) exposure on ocular health in India.

Name of Principal Investigator: **Prof. Radhika Tandon, Professor Tel. No. (S) 26593145**

The contents of the information sheet dated - - /- -/- - - - that was provided have been read carefully by me / explained in detail to me, in a language that I comprehend, and I have fully understood the contents. I confirm that I have had the opportunity to ask questions.

The nature and purpose of the study and its potential risks / benefits and expected duration of the study, and other relevant details of the study have been explained to me in detail. I understand that my participation is voluntary and that I am free to withdraw at any time, without giving any reason, without my medical care or legal right being affected.

I understand that the information collected about me from my participation in this research and sections of any of my medical notes may be looked at by responsible individuals from AIIMS. I give permission for these individuals to have access to my records.

I agree to take part in the above study.

Date:
Place:

(Signatures / Left Thumb Impression) _____________________________
Name of the Participant: ______________________________________
Son / Daughter / Spouse of: ___________________________________
Complete postal address: ______________________________________

This is to certify that the above consent has been obtained in my presence.

Date:
Place:

Signatures of Investigator

1) Witness – 1

Name: -----------------------------

Address: ------------------------------

2) Witness – 2

Name: -------------------------------

Address: -----------------------------

Version 2.0

<u>रोगी सूचना प्रपत्र</u>

<u>परियोजना का शीर्षकः – भारत में नेत्र स्वास्थ्य पर वातावरण बदलाव तथा अल्ट्रावॉयलेट रेडिऐशन (यू०वी०आर०) के प्रभाव का बहु केन्द्रीय सहयोगी अध्ययन।</u>

डॉ० राजेन्द्र प्रसाद नेत्र विज्ञान केन्द्र, अखिल भारतीय आयुर्विज्ञान संस्थान, नई दिल्ली द्वारा अल्ट्रावॉयलेट रेडिऐशन तथा नेत्र रोगों से संबंधित एक अध्ययन संचालित किया गया है। पिछले दशको में अल्ट्रावॉयलेट रेडिऐशन ने प्राकृतिक वातावरण को प्रभाकवत करने के साथ–साथ नेत्र स्वास्थ्य को भी महत्त्वपूर्ण रूप से प्रभावित किया है। जलवायु परिवर्त्तन के कारण आँख की ऊपरी सतह, कार्निया, लैंस तथा रेटिना पर दुष्प्रभाव पड़ा है। इस अध्ययन का मुख्य उद्देश्य अल्ट्रावायलेट के कारण उत्पन्न नेत्र क्षति है।भारत में मोतियाबिंद अंधता का एक मुख्य कारण है। इस अध्ययन के द्वारा हम यू०वी० रेडिएश्न तथा विभिन्न नेत्र रोग जैसे मोतियाबिंद, शुष्क नेत्र, टेरीजियम तथा स्प्रिंग नजला (वी०के०सी०) के उपचार के सहसंबंध अति गंभीर समस्याओं को रोकने में सहायता प्राप्त होगी।

उपर्युक्त जानकारी प्राप्त करने के लिए हमें वी०के०सी० के लिए 5 से 15 वर्ष की आयु वर्ग के बच्चो की तथा मोतियाबिंद, शुष्क नेत्र तथा टेरीजियम के लिए 40 वर्ष से अधिक आयु वर्ग के व्यक्तियों के नेत्रो की जॉच होगी। आपको एक प्रश्नावली दी जाएगी। सामान्यमानक के अनुसार डॉ० राजेन्द्र नेत्र विज्ञान, केन्द्र, अखिल भारतीय आयुर्विज्ञान संस्थान, नई दिल्ली में आपकी ऑखो का उपचार एवं संपूर्ण जांच की जाएगी। नेत्र परीक्षण के दौरान नेत्र डॉप्स की कुछ बूंदो द्वारा आपकी पुतली को विस्तारित किया जाएगा। इससे 1-2 दिनों तक आपको धुंधला दिखाई दे सकता है जो पूर्णतः हानिरहित है। यह स्वतः ही ठीक हो जाएगा। विशेष कैमरो द्वारा आप के नेत्रों के लैंसो की तस्वीरें ली जाएगी। उपर्युक्त सभी परीक्षण एवं जाँच एक बार में लगभग 3 घंटो में पूरी हो जाएंगे।

आप के द्वारा दी गई सभी जानकारियाँ तथा सभी परीक्षणों के परिणाम गोपनीय रखा जाएगा। इस अध्ययन में शामिल होने से आपको किसी प्रकार की हानि नही होगी।आप इस अध्ययन में सम्मिलित होने अथवा इसे बीच में छोड़ देने के लिए स्वतंत्र है। आपकी नेत्र संबंधी समस्याओं का निःशुल्क उपचार किया जाएगा। आपकी इस अध्ययन में सहभागिता अथवा अध्ययन छोड़ देने से आपके उपचार पर कोई प्रभाव नही पड़ेगा।

आपसे अनुरोध है कि इससे अतिरिक्त किसी भी जानकारी अथवा स्पष्टीकरण के लिए आप किसी भी समय निम्न व्यक्तियों से संपर्क कर सकते है :–

आचार्या राधिका टडंन, आचार्या **26593145**

डॉ० प्रवीण वशिष्ठ, सह आचार्य **9868398410, 26593143**

रोगी सूचित स्वीकृति पत्र

इस ट्रायल के लिए रोगी की आईडीः ....

परियोजना का शीर्षकः – भारत में नेत्र स्वास्थ्य पर वातावरण बदलाव तथा अल्ट्रावॉयलेट रेडिऐशन (यू०वी०आर०) के प्रभाव का बहु केन्द्रीय सहयोगी अध्ययन।

मुख्य अन्वेषक का नामः **आचार्या राधिका टंडन 26593145**

इस सूचना पत्र की सामग्री दिनांक .... को प्रदान की गई थी, मैने सावधानीपूर्वक पढ़लिया है। मुझे उस भाषा में विस्तार से समझा दिया गया है जो मुझे समझ में आती है और मैंने पूरी सामग्रीको अच्छी तरह समझ लिया है। मैं पुष्टि करता / करती हूँ कि मुझे प्रश्न पूछने का अवसर दिया गया है।

अध्ययन का प्रकार और प्रयोजन तथा इसकेसंभावित जोखिम / लाभ और अध्ययन पूरा होने की अनुमानित आवधि तथा अध्ययन के अन्य संगत विवरण मुझे विस्तार से समझा दिये गए हैं। मुझे बताया गयाहै कि मेरी भागीदारी स्वेच्छानुसार है और मैं कोई कारण बताए बिना किसी भी समय वापस जाने के लिए पूरी तरह स्वतंत्र हूँ और इस पर मेरी चिकित्सा देखभाल या कानूनी आधिकारों पर कोई प्रभाव नही पड़ेगा।

मुझे पता है कि इस अनुसंधान में मेरी भागीदारी केबारे में जमा की गई जानकारी और मेरे चिकित्सा नोट्स अखिल भारतीय आयुर्विज्ञान संस्थान (एम्स) के जिम्मेदार व्यक्तियों द्वारा देखी जाएगी, जो विनियामक प्राधिकरणों से हैं, जहां इस अनुसंधान में मेरे भाग लेने को संगत पाया जाए। मैं इन व्यक्तियों को अपने अभिलेख देखने की अनुमति देता/देती हूँ।

मैं उपरोक्त अध्ययन में भाग लेने के लिए सहमत हूँ।

दिनांकः ....

स्थान ः ....

हस्ताक्षर/बांए अंगूठे का निशानः ....

सहभागी का नामः ....

पुत्र/पुत्री/जीवन साथीः ....

डाक का पूरा पताः ....

यह प्रमाणित किया जाता है किउपरोक्त स्वीकृति मेरी उपस्थिति में प्राप्त की गई है।

अन्वेषककेहस्ताक्षर

दिनांकः ....

स्थानः ....

गवाह – 1

गवाह का नाम ....

डाक का पूरा पता ....

गवाह – 2

गवाह का नाम ....…..

डाक का पूरा पता ....

Form I
Household Enumeration Form
(PLEASE ENCIRCLE AND WRITE THE GIVEN RESPONSE)

IDENTIFICATION DATA	RESPONSE CATEGORIES	FOR OFFICE USE ONLY
Study Location<br>प्रोजेक्ट की जगह	**Delhi=1, Guwahati=2, Chennai=3**<br>दिल्ली =1, गोवाहाटी =2, चेन्नई = 3	[ ]
Name of village<br>ग्राम का नाम	______________________________	
Cluster Code<br>समूह संख्या एवं कोड		[ ] [ ]
Household ID<br>मकान का दिया गया नम्बर	Study House No. ______________<br>मकान का दिया गया नम्बर __________	[ ] + [ ] [ ] + [ ] [ ] [ ]<br>Location 1D (1) +Cluster 1D (2) +HH No (3)
Interviewer Name/ Code<br>साक्षात्कार लेने वाले का नाम / कोड	[ ] [ ]	[ ] [ ]
Date of Enumeration<br>गणना की तिथि	**(dd/mm/yyyy)** _ _ / _ _/_ _ _ _	[ ] [ ] – [ ] [ ] – [ ] [ ]
Name of respondent:<br>उत्तरदाता का नाम		
Relation to Head of the House Hold (HOH)<br>परिवार के मुखिया से सम्बन्ध	01. Self मुखिया<br>02. Spouse मुखिया की पत्नी /पति<br>03. Father पिता<br>04. Mother माता<br>05. Son बेटा<br>06. Daughter बेटी<br>07. Brother भाई<br>08. Sister बहन<br>09. Daughter-in-law बहु<br>10. Grand Son पोता/नाती<br>11. Grand Daughter पोती/ नतनी<br>12. Servant नौकर<br>66. Other(Specify) अन्य (उल्लेख करे) ______________	[ ] [ ]
Religion<br>धर्म	1.Hindu, 2.Muslim, 3.Sikh, 4.Christian 66.Others(specify)<br>1. हिन्दू, 2. मुस्लिम, 3.सिख, 4. ईसाई,<br>66.अन्य (उल्लेख करें) ___________________________	[ ]
Caste<br>जाति	1.SC/ST, 2.OBC, 3.General<br>1. अ०सु०जा०/अ०सु०ज०जा०, 2. अन्य पिछड़ी जाति, 3. सामान्य,	[ ] [ ]

Number of rooms in the house: excluding kitchen, toilets and bathrooms.<br>मकान में कमरों की सख्यंाः रसोई, शौचालय व नहाने के कमरे को छोड़कर।	Exact Number<br>वास्तविक संख्या ____________	[ ] [ ]
Landholdings (Cultivable)<br>कृषि योग्य भूमि	00. No land holding 01. Less than / equal to one acre ☐<br>Give exact number of acres if more than one<br>00. कोई भूमि नहीं , 01. एक किले से कम / बराबर,<br>यदि एक किले से अधिक हैं तो किलों की सही संख्या __________	[ ] [ ]
Total family income per month by all sources<br>कुल मिलाकर परिवार की मासिक आय	₹ ____________________<br>99. Not known 99. नहीं पता	

Total Number of Household Members<br>(Those staying continuously for the last 6 months or more)<br>घर के सदस्यों की कुल संख्या<br>(जो लगातार पिछले छः माह या अधिक से रह रहें है)	Actual No. of persons (including children of all ages)<br>सदस्यों की वास्तविक संख्या (सभी उम्र के बच्चों को मिलाकर) __________<br>00-04 years (00-04 वर्ष) _____________<br>05-15 years (05-15 वर्ष) _____________<br>16-39 years (16-39 वर्ष) _____________<br>40+ years (40+ वर्ष) _____________	[ ] [ ]<br>[ ] [ ]<br>[ ] [ ]<br>[ ] [ ]<br>[ ] [ ]

Details of all members in the household (Start with oldest living member)
मकान में रहने वाले सभी सदस्यों की जानकारी (सबसे बड़े जीवित सदस्य से शुरु करें)

S. No	Unique ID No	Name	Relation to HOH	Age	Gender	Marital Status	Education	Current Occupation	Type of resident	VKC	
										RE	LE
1.											
2.											
3.											
4.											
5.											
6.											
7.											
8.											
9.											
10.											
11.											
12.											
13.											
14.											
15.											

Relation	Age	Marital Status	Education	Occupation	Type of Resident
01. Self मुखिया<br>02. Spouse मुखिया की पत्नी /पति<br>03. Father पिता<br>04. Mother माता<br>05. Son बेटा<br>06. Daughter बेटी<br>07. Brother भाई<br>08. Sister बहन<br>09. Daughter-in-law बहु<br>10. Grand Son पोता/नाती<br>11. Grand Daughter पोती/ नतनी<br>12. Servant नौकर<br>66. Other(Specify) अन्य (उल्लेख करे)<br>_______________	Actual No. of years ---------<br><br>99. Not known<br><br>**Gender**<br><br>1. Male<br>2. Female	1. Married<br>2. Unmarried<br>3. .Divorced<br>4. Separated<br>5. Widow/Widower<br>33. Not applicable<br>66. Others (Specify)<br>____________	00. Illiterate,<br>50. Can read & write<br>1-12. Years of schooling<br>14. Diploma<br>15. Graduation<br>17. Post Graduation<br>20. Professional Education<br>33. Not applicable<br>66. Others (specify)<br>_______________<br>99. Not known	01. House work<br>02. Cultivator<br>03. Agricultural laborer<br>04. Non Agricultural laborer<br>05. Skilled worker<br>06. Office Job (Class I)<br>07. Office Job(Class II/III)<br>08. Office Job(Class IV)<br>09. Business<br>10. Professional (Doctor, Engineer, Lawyer etc.)<br>11. Unemployed<br>12. Retired/ Not working because of old age<br>13. Not working because of handicap/ sickness<br>14. Student<br>33. Not applicable<br>66.Others (specify)___________________	1. Usual resident<br>2. Resident for less than 6 months<br><br>**CO/ VKC**<br>0. None<br>1. Vernal kerato conjunctivitis (VKC)

Status of Enumeration Data<br>गणना की स्थिति	1.Completed interview (पूर्ण साक्षात्कार) ☐<br>2.Incomplete interview (अपूर्ण साक्षात्कार)<br>3.Refused (मना कर दिया)<br>66.Others (Specify) अन्य (उल्लेख करें) _________	[ ]

Date of First Visit for Enumeration: ________________________

Date of Second Visit for Enumeration: ________________________

Date of Third Visit for Enumeration: ________________________

Signature of Enumerator **Name of Enumerator**

UVR FORM II

INDIVIDUAL RISK ASSESSMENT QUESTIONNAIRE ≥40 YRS

PLEASE ENCIRCLE THE GIVEN RESPONSE

**IDENTIFICATION DATA**<br>**पहचान तथ्य**	**RESPONSE CATEGORIES**<br>**उत्तरदाता श्रेणी**
Study Location<br>प्रोजेक्ट की जगह	☐ **Delhi=1, Guwahati=2, Chennai=3**<br>दिल्ली =1, गोवाहाटी =2, चेन्नई = 3
Name of Village<br>ग्राम का नाम	____________________________
Cluster Code<br>समूह संख्या	☐☐
House No.<br>मकान का दिया गया नम्बर	☐☐☐
Person No.<br>व्यक्ति नम्बर	☐☐
Person Unique ID No.<br>**(From Enumeration Form)**<br>व्यक्ति को दिया गया नम्बर	☐ - ☐☐ - ☐☐☐ - ☐☐<br>Location ID (1) + Cluster ID (2) + HH No (3) + Person No (2)
Respondent Name<br>उत्तरदाता का नाम	
Gender<br>लिंग	Male=1; Female=2<br>पुरूष =1; स्त्री =2
Age<br>उम्र	_________ (In completed years)
Interviewer Name and Code<br>साक्षात्कारकर्ता का नाम एवं कोड	☐☐
Date of Interview<br>साक्षात्कार की तिथि	**(dd/mm/yyyy)** _ _/ _ _/_ _ _ _

SECTION A: OUTDOOR EXPOSURE

PRESENT ACTIVITY

1. What type of work are you mainly involved in throughout the day?

(Multiple responses possible)

आप की प्रत्येक दिन की दिनचर्या क्या है? **(एक से अधिक उत्तर संभव)**

1. Agricultural work (खेती का कार्य) ☐
2. Outdoor Non Agricultural Work (घर के बाहर अन्य कार्य) ☐
3. Indoor work (घर के अन्दर का कार्य) ☐

2. For how many years have you been doing this kind of work?
आप यह कार्य कितने वर्षो से कर रहे हैं?

_________ years (वर्ष)

3. How many hours do you usually spend outdoor after sunrise and before sunset? (9:00 AM to 5:00 PM) **Record in decimal form (eg: 1:30hr = 1.5hr)**
आप सूरज निक्लने से सूरज डूबने तक प्रायः कितने घंटे घर से बाहर बिताते हैं ? (सुबह 9:00 बजे से शाम 5:00 बजे तक) 0 = Nil (कुछ नहीं)

_________ hours (घंटे)

4. For how many hours are you usually outdoors in the middle of the day? (From 11:00 AM to 3:00 PM)
आमतौर पर आप दोपहर में कितने घंटे घर से बाहर बिताते है? (सुबह 11:00 बजे से दोपहर 3:00 बजे तक)

_________ hours (घंटे)

5. What type of head gear or eye gear do you normally wear when outside b/w 9 AM to 5 PM?
आमतौर पर आप जब धूप में जाते है तो सूबह 9:00 बजे से 5:00 बजे तक सिर व आँख ढ़कने के लिए क्या इस्तेमाल करते है ? **Record in decimal form (eg: 1:30hr = 1.5hr)**

	घंटे प्रति दिन
0. None (कुछ नहीं)	……….
1. Veil/ Dupatta/Saree pallu/ Ghunghat(बूरका / दूपट्टा / साड़ी पल्लू / घूँघट)	……….
2. Pagdi/saroopa/ mundas /towel(पगड़ी / सरूपा / मुंडास / तौलीया)	……….
3. Umbrella(छाता)	……......
4. Cap (टोपी)	……......
5. Sunglasses/prescription glasses (धूप का चश्मा / नम्बर वाला चश्मा)	……......
66. Others अन्य (उल्लेख करें) _________	…………

PAST ACTIVITY

6. Were you doing some other work in the past? **(Multiple responses possible)**

क्या आप इसके पहले दूसरा काम करते थे? **(एक से अधिक उत्तर संभव)**

0 . Not applicable (लागू नही) ☐
1. Agricultural work (खेती का कार्य) ☐
2. Outdoor Non Agricultural Work (घर के बाहर अन्य कार्य) ☐
3. Indoor work (घर के अन्दर का कार्य) ☐

7. For how many years did you follow this routine?
आप ने यह कार्य कितने वर्षो तक किया था?

_________ years (वर्ष)

8. How many hours did you usually spend outdoor after sunrise and before sunset? (9.00am to 5.00PM) **Record in decimal form (eg: 1:30hr = 1.5hr)**
आप सूरज निक्लने से सूरज डूबने तक प्रायः कितने घंटे घर से बाहर बिताते थे? (सुबह 9:00 बजे से शाम 5:00 बजे तक) 0 = Nil (कुछ नहीं)

________ hours (घंटे)

9. For how many hours were you usually outdoors in the middle of the day. (From 11:00 AM to 3:00 PM)
आमतौर पर आप दोपहर में कितने घंटे घर से बाहर बिताते थे? (सुबह 11:00 बजे से दोपहर 3:00 बजे तक)

_________ hours (घंटे)

10. What type of head gear or eye gear did you normally wear when outside b/w 9:00 AM to 5:00 PM ?
आमतौर पर आप जब धूप में जाते थे तो सूबह 9:00 बजे से शाम 5:00 बजे तक सिर व आँख ढ़कने के लिए क्या इस्तेमाल करते थे? **Record in decimal form (eg: 1:30hr = 1.5hr)**

	घंटे प्रति दिन
0. None (कुछ नहीं)	…………..
1. Veil/ Dupatta/Saree pallu/ Ghunghat(बूरका / दूपट्टा / साड़ी पल्लू / घूँघट)	…………..
2. Pagdi/saroopa/ mundas /towel(पगड़ी / सरूपा / मुंडास / तौलीया)	…………..
3. Umbrella (छाता)	…………..
4. Cap (टोपी)	…………...
5. Sunglasses/prescription glasses (धूप का चश्मा / नम्बर वाला चश्मा)	…………...
66. Others अन्य (उल्लेख करें) _________	………………

REMOTE PAST ACTIVITY

11. Were you doing some other work in the past? **(Multiple responses possible)**

क्या आप इसके पहले दूसरा काम करते थे? **(एक से अधिक उत्तर संभव)**

0 . Not applicable (लागू नही) ☐

1. Agricultural work (खेती का कार्य) ☐
2. Outdoor Non Agricultural Work (घर के बाहर अन्य कार्य) ☐
3. Indoor work (घर के अन्दर का कार्य) ☐

12. For how many years did you follow this routine?

आप ने यह कार्य कितने वर्षो तक किया था ?

_________ years (वर्ष)

13. How many hours did you usually spend outdoor after sunrise and before sunset? (9.00AM to 5.00PM) **Record in decimal form (eg: 1:30hr = 1.5hr)**

आप सूरज निकलने से सूरज डूबने तक प्रायः कितने घंटे घर से बाहर बिताते थे? (सुबह 9:00 बजे से शाम 5:00 बजे तक) 0 = Nil (कुछ नहीं)

________ hours (घंटे)

14. For how many hours were you usually outdoors in the middle of the day. (From 11:00 AM to 3:00 PM)

आमतौर पर आप दोपहर में कितने घंटे घर से बाहर बिताते थे? (सूबह 11:00 बजे से दोपहर 3:00 बजे तक)

_________ hours (घंटे)

15. What type of head gear or eye gear did you normally wear when outside b/w 9AM to5PM?

आमतौर पर आप जब धूप में जाते थे तो सुबह 9:00 बजे से शाम 5:00 बजे तक सिर व आँख ढ़कने के लिए क्या इस्तेमाल करते थे?

घंटे प्रति दिन

0. None (कुछ नहीं) …………..
1. Veil/ Dupatta/Saree pallu/ Ghunghat(बूरका / दूपट्टा / साड़ी पल्लू / घूँघट) …………..
2. Pagdi/saroopa/ mundas /towel(पगड़ी / सरूपा / मुंडास / तौलीया) …………..
3. Umbrella (छाता) …………..
4. Cap (टोपी) …………...
5. Sunglasses/prescription glasses (धूप का चश्मा / नम्बर वाला चश्मा) …………...
66. Others अन्य (उल्लेख करें) _________ ………………

SECTION B: INDOOR SMOKE EXPOSURE

KITCHEN (रसोईघर)

16. Do/Did you ever cook food/ spend time in the kitchen daily?

क्या प्रतिदिन आप खाना पकाने / रसोईघर में समय बिताते है/थे ?

Yes (हाँ) =1 No (नहीं) =2 **(If No, go to Q.No. 19)**

17. If yes, then how much time do you spend in the cooking place/kitchen every day?

यदि हाँ तो आप प्रतिदिन कितना समय खाना पकाने या रसोईघर में बिताते हैं/थे ?

Total Time (कुल) _______hours (घंटे)

18. For how many years have you been cooked food/spent time in the kitchen?

आप कितने वर्ष खाना बनाने /रसोई में समय बितायें है/थे ?

__________ years(वर्ष)

19. In your house what fuel is generally used? **(Multiple responses possible)**

सामान्यतः आपके घर में कौन सा ईंधन इस्तेमाल होता है /था?

(एक से अधिक उत्तर संभव है)

FUEL (ईंधन)

S.No. कोड	**Type of fuel** ईंधन के प्रकार	**Number of years**कितने साल से		
		**Present**	**Past**	**Remote past**
1.	Wood/Crop residues/Dung cakes (लकड़ी/ फसल का बचा हुआ हिस्सा /उपले)			
2.	Coal/coke/lignite/ Charcoal (कोयला / लकड़ी का कोयला)			
3.	Kerosene (किरोसिन)			
4.	Electricity (बिजली का हीटर)			
5.	LPG (एल०पी०जी०)			
6.	Bio Gas/Gobar Gas/Solar Cooker (वायोगैस/गोबरगैस)			

SECTION C: SMOKING HABITS

20. Have you ever smoked tobacco daily for more than three months?
क्या आप ने कभी लगातार तीन महीने से अधिक समय तक प्रतिदिन धूम्रपान किया है?

Yes (हाँ) = 1 No (नही) = 2 **(If No then finish the form III)**

21. If yes, for how long have you been smoking/smoked tobacco?
यदि हाँ तो आपने कितने समय तक धूम्रपान किया है?

__________ months/ years(महीने / वर्ष)

22. What do you smoke? **(Multiple responses possible)**
धूम्रपान के लिए आप क्या इस्तेमाल करते है? (एक से अधिक उत्तर संभव)

1. Cigarette (सिगरेट) ☐
2. Bidi (बीड़ी) ☐
3. Hukka (हुक्का) ☐

66. Others (Specify) (अन्य उल्लेख करें) ___________

23. How many cigarettes/bidi/chillum do you smoke each day?
आप प्रतिदिन कितने सिगरेट/बीड़ी/हुक्का पीते है?

	Present	
	Years	Numbers
1. Cigarette (सिगरेट)		
2. Bidi (बीड़ी)		
3. Hukka (हुक्का)		
**66.** Others (Specify) (अन्य उल्लेख करें)		

If you do not smoke now or if the pattern of smoking was different in the past then
यदि आप वर्तमान में धूम्रपान नहीं करते या धूम्रपान के तरीके में कोइ बदलाव आया है तो

24. What did you smoke? **(Multiple responses possible)**
पहले आप धूमपान के लिए क्या इस्तेमाल करते थे ? (एक से अधिक उत्तर संभव)

1. Cigarette (सिगरेट) ☐
2. Bidi (बीड़ी) ☐
3. Hukka (हुक्का) ☐

66. Others (Specify) (अन्य उल्लेख करें) ___________

25. How many cigarettes/bidi/chillum did you smoke each day?
धूमपान के लिए आप क्या इस्तेमाल करते थे ?

	Past	
	Years	Numbers
**1.** Cigarette (सिगरेट)		
**2.** Bidi (बीड़ी)		
**3.** Hukka (हुक्का)		
**66.** Others (Specify) (अन्य उल्लेख करें)		

UVR FORM III: Ocular Surface Disease Index (OSDI)

नेत्र ऊपरी रोग सूचक (ऑक्यूलर सरफेस डिजीज इन्डेक्स)

Ask your participant the following 12 questions, and circle the number in the box that best represents each answer.

उत्तरदाता से निम्नलिखित १२ सवाल पूछें, और सर्वोत्तम जवाब केअंक को गोला करे

Have you experienced any of the following during the last month :-
क्या आपने पिछले महीने निम्न में से किन्हीं तकलीफ का अनुभव किया है?

	All of the time हर समय	Most of the time ज्यादा समय	Half of the time आधे समय	Some of the time कुछ समय	None of the time कभी नहीं
1. Eyes that are sensitive to light? आँखों का रोशनी से प्रभावित होना	4	3	2	1	0
2. Eyes that feel gritty? आँखों में किरकिरापन महसूस होना	4	3	2	1	0
3. Painful or sore eyes? आँखों में दर्द या जलन	4	3	2	1	0
4. Blurred vision (आँखों में धुँधलापन)	4	3	2	1	0
5. Poor vision (नजर कमजोर होना) ?	4	3	2	1	0
**Subtotal score for answers 1 to 5:** ☐ **A**					

Have problems with your eyes limited you in performing any of the following during the last month (क्या पिछले महीने से आँखों की तकलीफ के कारण आप निम्न कार्यो को नहीं कर पा रहे हैं ?)

	All of the time हर समय	Most of the time ज्यादा समय	Half of the time आधे समय	Some of the time कुछ समय	None of the time कभी नहीं	Not applicable लागू नहीं है
6. Reading (पढ़ना)	4	3	2	1	0	N/A
7. Driving at night? रात में गाड़ी चलाना	4	3	2	1	0	N/A
8. Working with a computer or bank machine (ATM)? कम्प्यूटर या एटीम पर काम करना	4	3	2	1	0	N/A
9. Watching TV? टी०वी० देखना	4	3	2	1	0	N/A
**Subtotal score for answers 6 to 9:** ☐ **B**						

Have your eyes felt uncomfortable in any of the following situations during the last month?
क्या पिछले महीने से निम्न में से किसी परिस्थितियों में आपकी आँखों में तकलीफ हुई है?

	All of the time हर समय	Most of the time ज्यादा समय	Half of the time आधे समय	Some of the time कुछ समय	None of the time कभी नहीं	Not applicable लागू नहीं है
10. Windy conditions? तेज हवा के कारण	4	3	2	1	0	N/A
11. Places or areas with low humidity (very dry)? कम उमस वाली जगहों में (अत्यधिक शुष्क)	4	3	2	1	0	N/A
12. Areas that are air conditioned? वातानुकूलित (एयर कंडीशंड) जगहों में	4	3	2	1	0	N/A

Subtotal score for answers 10 to 12: ☐ **C** **Total score (A+B+C) =** ☐ **Total no. of questions answered =** ☐

Status of Interview<br>साक्षात्कार की स्थिति	1. Completed interview (पूर्ण साक्षात्कार)<br>2. Incomplete interview (अपूर्ण साक्षात्कार)<br>3. Refused (मना कर दिया)<br>66.Others (Specify) अन्य (उल्लेख करें) __________

Signature of Interviewer:

Name of Interviewer:

SPECIAL OBSERVATIONS AND REMARKS

<u>रोगी सूचना प्रपत्र</u>

<u>परियोजना का शीर्षक</u>ः — <u>भारत में नेत्र स्वास्थ्य पर वातावरण बदलाव तथा अल्ट्रावॉयलेट रेडिऐशन (यू०वी०आर०) के प्रभाव का बहु केन्द्रीय सहयोगी अध्ययन।</u>

डॉ० राजेन्द्र प्रसाद नेत्र विज्ञान केन्द्र, अखिल भारतीय आयुर्विज्ञान संस्थान, नई दिल्ली द्वारा अल्ट्रावॉयलेट रेडिऐशन तथा नेत्र रोगों से संबंधित एक अध्ययन संचालित किया गया है। पिछले दशको में अल्ट्रावॉयलेट रेडिऐशन ने प्राकृतिक वातावरण को प्रभाकवत करने के साथ—साथ नेत्र स्वास्थ्य को भी महत्त्वपूर्ण रूप से प्रभावित किया है। जलवायु परिवर्त्तन के कारण आँख की ऊपरी सतह, कार्निया, लैंस तथा रेटिना पर दुष्प्रभाव पड़ा है। इस अध्ययन का मुख्य उद्देश्य अल्ट्रावायलेट के कारण उत्पन्न नेत्र क्षति है।भारत में मोतियाबिंद अंधता का एक मुख्य कारण है। इस अध्ययन के द्वारा हम यू०वी० रेडिएश्न तथा विभिन्न नेत्र रोग जैसे मोतियाबिंद, शुष्क नेत्र, टेरीजियम तथा स्प्रिंग नजला (वी०के०सी०) के उपचार के सहसंबंध अति गंभीर समस्याओं को रोकने में सहायता प्राप्त होगी।

उपर्युक्त जानकारी प्राप्त करने के लिए हमें वी०के०सी० के लिए 5 से 15 वर्ष की आयु वर्ग के बच्चो की तथा मोतियाबिंद, शुष्क नेत्र तथा टेरीजियम के लिए 40 वर्ष से अधिक आयु वर्ग के व्यक्तियों के नेत्रो की जॉच होगी। आपको एक प्रश्नावली दी जाएगी। सामान्यमानक के अनुसार डॉ० राजेन्द्र नेत्र विज्ञान, केन्द्र, अखिल भारतीय आयुर्विज्ञान संस्थान, नई दिल्ली में आपकी ऑखो का उपचार एवं संपूर्ण जांच की जाएगी। नेत्र परीक्षण के दौरान नेत्र डॉप्स की कुछ बूंदो द्वारा आपकी पुतली को विस्तारित किया जाएगा। इससे 1-2 दिनों तक आपको धुंधला दिखाई दे सकता है जो पूर्णतः हानिरहित है। यह स्वतः ही ठीक हो जाएगा। विशेष कैमरो द्वारा आप के नेत्रों के लैंसो की तस्वीरें ली जाएगी। उपर्युक्त सभी परीक्षण एवं जाँच एक बार में लगभग 3 घंटो में पूरी हो जाएंगे।

आप के द्वारा दी गई सभी जानकारियाँ तथा सभी परीक्षणों के परिणाम गोपनीय रखा जाएगा। इस अध्ययन में शामिल होने से आपको किसी प्रकार की हानि नही होगी।आप इस अध्ययन में सम्मिलित होने अथवा इसे बीच में छोड़ देने के लिए स्वतंत्र है। आपकी नेत्र संबंधी समस्याओं का निःशुल्क उपचार किया जाएगा। आपकी इस अध्ययन में सहभागिता अथवा अध्ययन छोड़ देने से आपके उपचार पर कोई प्रभाव नही पड़ेगा।

आपसे अनुरोध है कि इससे अतिरिक्त किसी भी जानकारी अथवा स्पष्टीकरण के लिए आप किसी भी समय निम्न व्यक्तियों से संपर्क कर सकते है :—

आचार्या राधिका टडंन, आचार्या **26593145**

डॉं० प्रवीण वशिष्ठ, सह आचार्य **9868398410, 26593143**

<u>रोगी सूचित स्वीकृति पत्र</u>

इस ट्रायल के लिए रोगी की आईडीः ....

परियोजना का शीर्षकः – भारत में नेत्र स्वास्थ्य पर वातावरण बदलाव तथा अल्ट्रावॉयलेट रेडिऐशन (यू०वी०आर०) के प्रभाव का बहु केन्द्रीय सहयोगी अध्ययन।

मुख्य अन्वेषक का नामः **आचार्या राधिका टडंन 26593145**

इस सूचना पत्र की सामग्री दिनांक .... को प्रदान की गई थी, मैने सावधानीपूर्वक पढ़लिया है। मुझे उस भाषा में विस्तार से समझा दिया गया है जो मुझे समझ में आती है और मैंने पूरी सामग्रीको अच्छी तरह समझ लिया है। मैं पुष्टि करता / करती हूँ कि मुझे प्रश्न पूछने का अवसर दिया गया है।

अध्ययन का प्रकार और प्रयोजन तथा इसके संभावित जोखिम / लाभ और अध्ययन पूरा होने की अनुमानित आवधि तथा अध्ययन के अन्य संगत विवरण मुझे विस्तार से समझा दिये गए हैं। मुझे बताया गयाहै कि मेरी भागीदारी स्वेच्छानुसार है और मैं कोई कारण बताए बिना किसी भी समय वापस जाने के लिए पूरी तरह स्वतंत्र हूँ और इस पर मेरी चिकित्सा देखभाल या कानूनी आधिकारों पर कोई प्रभाव नही पड़ेगा।

मुझे पता है कि इस अनुसंधान में मेरी भागीदारी के बारे में जमा की गई जानकारी और मेरे चिकित्सा नोट्स अखिल भारतीय आयुर्विज्ञान संस्थान (एम्स) के जिम्मेदार व्यक्तियों द्वारा देखी जाएगी, जो विनियामक प्राधिकरणों से हैं, जहां इस अनुसंधान में मेरे भाग लेने को संगत पाया जाए। मैं इन व्यक्तियों को अपने अभिलेख देखने की अनुमति देता हूँ।

मैं उपरोक्त अध्ययन में भाग लेने के लिए सहमत हूँ।

दिनांकः ....

स्थान : ....

हस्ताक्षर/बांए अंगूठे का निशानः ....

सहभागी का नामः ....

पुत्र/पुत्री/जीवन साथीः ....

डाक का पूरा पताः ....

यह प्रमाणित किया जाता है कि उपरोक्त स्वीकृति मेरी उपस्थिति में प्राप्त की गई है।

अन्वेषक के हस्ताक्षर

दिनांकः ....

स्थानः ....

गवाह – 1	गवाह – 2
गवाह का नाम	गवाह का नाम ....
डाक का पूरा पता	डाक का पूरा पता ....

<u>रोगी सूचना प्रपत्र</u>

<u>परियोजना का शीर्षकः</u> – <u>भारत में नेत्र स्वास्थ्य पर वातावरण बदलाव तथा अल्ट्रावॉयलेट रेडिऐशन (यू०वी०आर०) के प्रभाव का बहु केन्द्रीय सहयोगी अध्ययन।</u>

डॉ० राजेन्द्र प्रसाद नेत्र विज्ञान केन्द्र, अखिल भारतीय आयुर्विज्ञान संस्थान, नई दिल्ली द्वारा अल्ट्रावॉयलेट रेडिऐशन तथा नेत्र रोगों से संबंधित एक अध्ययन संचालित किया गया है। पिछले दशको में अल्ट्रावॉयलेट रेडिऐशन ने प्राकृतिक वातावरण को प्रभाकवत करने के साथ–साथ नेत्र स्वास्थ्य को भी महत्त्वपूर्ण रूप से प्रभावित किया है। जलवायु परिवर्त्तन के कारण आँख की ऊपरी सतह, कार्निया, लैंस तथा रेटिना पर दुष्प्रभाव पड़ा है। इस अध्ययन का मुख्य उद्देश्य अल्ट्रावायलेट के कारण उत्पन्न नेत्र क्षति है।भारत में मोतियाबिंद अंधता का एक मुख्य कारण है। इस अध्ययन के द्वारा हम यू०वी० रेडिएश्न तथा विभिन्न नेत्र रोग जैसे मोतियाबिंद, शुष्क नेत्र, टेरीजियम तथा स्प्रिंग नजला (वी०के०सी०) के उपचार के सहसंबंध अति गंभीर समस्याओं को रोकने में सहायता प्राप्त होगी।

उपर्युक्त जानकारी प्राप्त करने के लिए हमें वी०के०सी० के लिए 5 से 15 वर्ष की आयु वर्ग के बच्चो की तथा मोतियाबिंद, शुष्क नेत्र तथा टेरीजियम के लिए 40 वर्ष से अधिक आयु वर्ग के व्यक्तियों के नेत्रो की जॉच होगी। आपको एक प्रश्नावली दी जाएगी। सामान्यमानक के अनुसार डॉ० राजेन्द्र नेत्र विज्ञान, केन्द्र, अखिल भारतीय आयुर्विज्ञान संस्थान, नई दिल्ली में आपकी ऑखो का उपचार एवं संपूर्ण जांच की जाएगी। नेत्र परीक्षण के दौरान नेत्र डॉप्स की कुछ बूंदो द्वारा आपकी पुतली को विस्तारित किया जाएगा। इससे 1-2 दिनों तक आपको धुंधला दिखाई दे सकता है जो पूर्णतः हानिरहित है। यह स्वतः ही ठीक हो जाएगा। विशेष कैमरो द्वारा आप के नेत्रों के लैंसो की तस्वीरें ली जाएगी। उपर्युक्त सभी परीक्षण एवं जाँच एक बार में लगभग 3 घंटो में पूरी हो जाएंगे।

आप के द्वारा दी गई सभी जानकारियाँ तथा सभी परीक्षणों के परिणाम गोपनीय रखा जाएगा। इस अध्ययन में शामिल होने से आपको किसी प्रकार की हानि नही होगी।आप इस अध्ययन में सम्मिलित होने अथवा इसे बीच में छोड़ देने के लिए स्वतंत्र है। आपकी नेत्र संबंधी समस्याओं का निःशुल्क उपचार किया जाएगा। आपकी इस अध्ययन में सहभागिता अथवा अध्ययन छोड़ देने से आपके उपचार पर कोई प्रभाव नही पड़ेगा।

आपसे अनुरोध है कि इससे अतिरिक्त किसी भी जानकारी अथवा स्पष्टीकरण के लिए आप किसी भी समय निम्न व्यक्तियों से संपर्क कर सकते है :–

आचार्या राधिका टडंन, आचार्या **26593145**

डॉ० प्रवीण वशिष्ठ, सह आचार्य **9868398410, 26593143**

UVR FORM IV

INDIVIDUAL RISK ASSESSMENT QUESTIONNAIRE (5-15 YRS)

**IDENTIFICATION DATA**<br>**पहचान तथ्य**	**RESPONSE CATEGORIES**<br>**उत्तरदाता श्रेणी**
Study Location<br>प्रोजेक्ट की जगह	☐ **Delhi=1, Guwahati=2, Chennai=3**<br>दिल्ली =1, गोवाहाटी =2, चेन्नई = 3
Name of Village<br>ग्राम का नाम	____________________
Cluster Code<br>समूह संख्या	☐☐
House No.<br>मकान नम्बर	☐☐☐
Child No.<br>बच्चे का नम्बर	☐☐
Child Unique ID No.<br>**(From Enumeration Form)**<br>बच्चे को दिया गया नम्बर	☐ - ☐☐ - ☐☐☐ - ☐☐<br>Location ID (1) + Cluster ID (2) + House No(3) + Person No (2)
Interviewer Code and Name<br>साक्षात्कारकर्ता का कोड एवं नाम	☐☐ ______________________
Respondent Name<br>उत्तरदाता का नाम	__________________________
Relationship to the child<br>बच्चे से संबंध	☐ Mother=1 Father=2<br>माता =1 पिता =2<br>66. Others(specify)<br>अन्य (उल्लेख करे)_______________
Date of Interview **(dd-mm-yyyy)**<br>साक्षात्कार की तिथि	☐☐ ☐☐ ☐☐☐☐
Name of child<br>बच्चे का नाम	______________________
Gender of the child	☐ Male=1 Female=2<br>पुरूष = 1 स्त्री = 2
Age of child (In completed years)<br>बच्चे का उम्र (निकटतम वर्ष में)	☐☐

Informant: Mother/Father (Please Encircle the right response)

सूचना दाता : माता/पिता (कृपया सही उत्तर पर गोला लगाये)

S. No.	DATA	RESPONSE CATEGORIES
1.	Is there a family history of allergy or asthma (breathing problem)?<br>क्या आप के परिवार में कभी किसी को एलर्जी या अस्थमा (सांस की) समस्या रही है ?	1= Yes 2 = No 99 = Not known<br>1= हाँ 2 = नहीं 99 = पता नही
2.	Are there any pets in the family?<br>क्या परिवार में कोई पालतू पशु है ?	1=Yes 2=No<br>1= हाँ 2= नहीं
3.	Does anybody smoke in your family?<br>क्या आप के परिवार में कोइ धूम्रपान करता है ?<br>**(If No, than go to Question No.05)**	1=Yes 2=No<br>1= हाँ 2= नहीं
4.	If yes, then what is the relation to head of the house hold?<br>यदि हाँ, तो उसका परिवार के मुखिया से क्या सम्बन्ध है ?<br>01. Self मुखिया<br>02. Spouse मुखिया की पत्नी /पति<br>03. Father पिता<br>04. Mother माता<br>05. Son बेटा<br>06. Daughter बेटी<br>07. Brother भाई<br>08. Sister बहन<br>09. Daughter-in-law बहु<br>10. Grand Son पोता/नाती<br>11. Grand Daughter पोती/ नतनी<br>12. Servant नौकर<br>66. Other(Specify) अन्य (उल्लेख करे) _______________	Name of the smoker<br>धूम्रपान करने वाले का नाम<br>________________________
5.	How many hours does the child usually spend outdoor after sunrise and before sunset? (9AM to 5PM) 0 = Nil<br>आप का बच्चा अपने सारे कार्यो के लिए सूरज निकलने से सूरज डूबने तक प्रायः कितने घंटे घर से बाहर बिताता है ?	__________ घंटे
6.	For how many hours is the child usually outdoors in the middle of the day (From 11 AM to 3 PM) 0= Nil<br>आमतौर पर आप का बच्चा दोपहर में कितने घंटे घर से बाहर बिताता है? (सूबह 11बजे से 3 बजे तक)	__________ घंटे
7.	What type of head/eye gear does the child normally wear when outdoors?<br>आप का बच्चा घर से बाहर निकलते समय सिर व आँख को ढ़कने के लिए क्या इस्तेमाल करता है ?	Hrs. Per day (घंटे प्रति दिन)<br>0. None (कुछ नहीं) : …………..<br>1. Cap/Hat(टोपी) …………..<br>2. Umbrella/Japi (छाता) …………..<br>3. Towel/Gamcha (तौलीया) ..…………<br>4. Sunglasses/prescription glasses ………..<br>(धूप का चश्मा / नम्बर वाला चश्मा)<br>66. Others(specify) (अन्य उल्लेख करें)<br>_____________ …………...

UVR FORM V

CLINICAL EXAMINATION FORM ≥40 YRS

SECTION A: IDENTIFICATION DATA

IDENTIFICATION DATA	RESPONSE
Study Location	☐ Delhi=1, Guwahati=2, Chennai=3
Name of village	________________
Cluster Code	☐☐
House No.	☐☐☐
Person Number	☐☐
Person Unique ID No.	☐ - ☐☐ - ☐☐☐ - ☐☐<br>Location ID (1) + Cluster ID (2) + House No(3) + Person No (2)
Respondent Name	________________
Respondent Age<br>(in completed years)	☐☐
Gender	☐ Male = 1 ; Female = 2
Optometrist Code/Name	☐☐ ______________________________
Ophthalmologist Code/Name	☐☐ ______________________________
Place of Examination	☐ 1. Base hospital<br>2. Central field site<br>3. Home Examination
Date of Examination<br>(dd/mm/yyyy)	☐☐ - ☐☐ - ☐☐☐☐
Photograph taken	☐ Yes=1, No=2

SECTION B: BLOOD PRESSURE, BLOOD SUGAR AND ANTHROPOMETRY

BLOOD PRESSURE (in mm Hg)

	**First measurement**	**Second measurement**
Systolic blood pressure		
Diastolic blood pressure		

If blood pressure not available please state reason why (circle one):

1. Patient refused
2. Machine gives error message
66. Other (specify): ____

WEIGHT

**State value in Kg up to one decimal point**	

If weight measurement not possible, please state reason why (circle one):

1. Participant refuses
2. Participant is chair/bed bound
3. Participant is too unsteady on feet
66. Other (specify): ____

STANDING HEIGHT

**State value in cm up to one decimal point**	

If standing height measurement not possible, please state reason why (circle one):

1. Participant refuses
2. Participant is chair/bed bound
3. Participant is too unsteady on feet
4. Participant has a bent spine
66. Other (specify): ____

MID UPPER ARM CIRCUMFERENCE

**State value (in cm) up to one decimal point**	
If circumference not taken, state the reason:	

BLOOD SUGAR

**State value in mg/dl up to one decimal point**	
If blood sugar not taken, state the reason:	

HISTORY OF SYSTEMIC DISEASE (YES-1; NO=2)

	Yes/ No	Duration (yrs)	Medical Treatment (Yes/ No)
1. DM			
2. HYPERTENSION			
3. HEART DISEASE			
4. ANY OTHER (Specify)			

SECTION C1: VISUAL ACUITY

**Wearing Glass (Yes=1, No=2)**						
**Vision**		**Right Eye**		**Left Eye**		
		**Acuity**	**Total 'E's read**	**Acuity**	**Total 'E's read**	
**Unaided**	**Distance**					
	**Near**					
**Presenting (With usual glasses)**	**Distance**					
	**Near**					

If vision not recorded, state the reason: **RE**____

LE____

SECTION C2: SUBJECTIVE ACCEPTANCE

Subjective Acceptance (Done/ Not Done)

If Not Done, Give reason: Corneal disease, Lenticular opacity, Patient Not Cooperative, Machine Error, Home Visit

**Acceptance**		**Sphere**	**Cylinder**	**Axis**	**Acuity**	**Total Es read**
**Right Eye**	**Distance**					
	**Near**	**Add**	**D sph**			
**Left Eye**	**Distance**					
	**Near**	**Add**	**DSph**			

SECTION C3 : AUTOREFRACTION

Autorefraction (Done/ Not Done)

If Not Done, Give reason: Corneal disease, Lenticular opacity, Patient Not Cooperative, Machine Error, Home Visit

		**Sphere**		**Cylinder**	**Axis**
**Right Eye**	+/_		+/_		
**Left Eye**	+/_		+/_		

SECTION D: DRY EYE TESTS

**PARAMETER**	**RIGHT EYE**	**LEFT EYE**
SCHIRMER'S TEST (mm)		
BREAK UP TIME (sec)		

If dry eye tests not done please state reason why (circle one):

1. Patient refused

66. Other (specify): ____________________

SECTION E: INTRAOCULAR PRESSURE

	RE	LE
Measured (**1= Yes; 2= No)**		
IOP in mm Hg		
**Measured with:**	1. NCT 2.Perkin's Applanation	
If not measured, Give reasons		

SECTION F1: BASIC EYE EXAMINATION

(Mark: 1= Present; 2=Not present; 9=Undetermined)

	RE	LE
1.Squint (a=LDS; b=LCS; c=RDS; d=RCS)		
2.Nystagmus		
3. Anterior staphyloma		
4. Phthisis/ Disorganized/Absent Globe/Microphthalmos/Anophthalmos		
5. Corneal Opacity (specify cause)		
6. Adherent leucoma		
7. Corneal Ulcer(Infective/shield ulcer)		
66.Others (Specify)		

If undetermined give reasons LE___________________
RE___________________

SECTION F2:BIOMICROSCOPY EXAMINATION

(Mark: 1= Present; 2=Not present; 9=Undetermined)

☐ **Not Done, please explain:** ____

**1.Pterygium**		
a. Location	Nasal /temporal	Nasal /temporal
b. Size(mm)		
c. Extent beyond corneal limbus		
d. Grade		
**2. Pingecula**		
**3. Corneal Staining**		
a. Location (Superior =1, Inferior=2, Nasal =3, Temporal=4, Central=5, Total/Diffuse=6)		
**4. Other Pathology**		
specify:		

If undetermined give reasons LE___________________
RE___________________

SECTION G: PUPIL DILATION

1. Were mydriatic drops instilled (Yes=1; No=2) **RE ______ LE ______**

If no, specify reason **RE ____________________LE__________________**

2. Was dilation = 6 mm (Yes=1; No=2) **RE _____________ LE__________**

SECTION H1: LENS STATUS (MULTIPLE RESPONSES ALLOWED)

(Present=1; Not present=2; Not seen=3; Undetermined=9)

	RE	LE
1. Normal		
2. Pseudoexfoliation		
3. Cortical Cataract		
4. Nuclear Cataract		
5. Posterior subcapsular cataract		
6. Advanced cataract		
7. Developmental cataract		
8. Traumatic cataract		
9. Aphakia		
10.Aphakia + PCO		
11.Pseudophakia		
12.Pseudophakia + PCO		
13.Dislocated or subluxated lens/IOL		
66.Others (specify)		

If not seen specify the reason RE ___________________ LE ___________________

If undetermined specify the reason RE ___________________ LE ___________________

SECTION H2: LOCS III GRADING

Details of LOCS grading: **Not Applicable RE** **Not Applicable LE**

EYES	LOCS GRADING				Reasons for ungradable
	Cortical grade (C)	PSC grade (P)	Nuclear opalescence (NO)	Nuclear colour (NC)	1= poor quality<br>2= advanced opacity<br>3=aphakia/ pseudophakia<br>4= pupils not dilated<br>5= patient Refused
Right Eye					
Left Eye					

H3. IF, PSEUDOPHAKIC/APHAKIC, DETAILS ABOUT CATARACT SURGERY

Not applicable RE **Not applicable LE**

	Right eye	Left eye
**1. Age at operation (years)**		
**2. Place of operation**		
Government hospital		
Voluntary/charitable hospital		
Private hospital		
Eye camp/improvised setting		
Traditional setting		
Don't know		
**3. Cost of surgery**		
Totally free		
Partially free		
Fully paid		
Don't know		
**4. Type of surgery**		
Non IOL		
IOL implant (PCIOL/ACIOL)		
Couching		
Don't know		
If aphakic, using aphakic glasses 1=Yes ;2= No<br>If No, state reason		

SECTION I: FUNDUS EXAMINATION

(Present=1; Not present=2; Not seen=3; Undetermined=9)

	RE	LE
1. Normal		
2. Dry ARMD		
3. Wet ARMD		
4. Optic Atrophy		
5. Glaucomatous cupping		
6. High Myopia		
7. Vascular Retinopathy		
8. Diabetic Retinopathy<br>Type(a=NPDR, b=PDR, c=Maculopathy)		
9. Chorioretinitis		
10. Other Maculopathy		
11. Significant Vitreous opacities		
12. Retinitis Pigmentosa		
13. Retinal Detachment		
14. Congenital anomaly		
66. Others (specify)		

If not seen specify the reason RE __________________ LE _____________

If undetermined specify the reason RE __________________ LE _____________

SECTION J: MAIN CAUSE PRESENTING VA<6/18

(Mark only one cause for each eye) (Present=1; Not present=2; Not seen=3; Undetermined=9)

	Not applicable RE	Not applicable LE	Mark one principal disorder
	**Right eye**	**Left eye**	
1. Phthisical, disorganised or absent globe			1
2. Refractive error			2
3. Cataract, untreated			3
4. Aphakia, uncorrected			4
5. Posterior capsular opacification			5
6. Trachoma			6
7. Optic Atrophy			7
8. Corneal opacity			8
9. Globe abnormality			9
10. Glaucoma			10
11. Diabetic Retinopathy			11
12. ARMD			12
13. Chorioretinitis			13
14. Vascular retinopathy			14
15. Amblyopia			15
66. Other post. segment /CNS			66
Not examined (can see 6/18)			

If not seen specify the reason RE __________________ LE ____________

If undetermined specify the reason RE __________________ LE ____________

Examination completed/ Not completed

PROTOCOL FOR VISUAL ACUITY TESTING

1. RIGHT EYE –UNAIDED

**Four Metres**							
						**Acuity** (Circle one)	**# of letters read**
First Row	E	m	Ǝ	E	w	6/60	
Second Row	w	Ǝ	E	w	E	6/48	
Third Row	m	E	m	Ǝ	E	6/38	
Fourth Row	w	E	Ǝ	E	Ǝ	6/30	
Fifth Row	m	Ǝ	w	w	m	6/24	
Sixth Row	E	w	Ǝ	m	w	6/19	
Seventh Row	w	m	E	Ǝ	E	6/15	
Eighth Row	Ǝ	w	Ǝ	w	E	6/12	
Ninth Row	Ǝ	E	w	E	m	6/9.5	
Tenth Row	w	m	E	m	w	6/7.5	
Eleventh Row	Ǝ	E	m	Ǝ	w	6/6	
**Total E's read at 4 meters (actual number of letters + 30)**							
**One Metre**							
First Row	m	Ǝ	w	E	Ǝ	1/40	
Second Row	w	E	Ǝ	m	w	1/32	
Third Row	m	E	w	E	E	1/25	
Fourth Row	w	Ǝ	m	Ǝ	E	1/20	
Fifth Row	E	Ǝ	w	E	m	1/16	
Sixth Row	Ǝ	E	w	m	Ǝ	1/12.5	
**Total E's read at 1 meter (actual number of letters)**							
Finger Counting close to face							FCCF
Hand Movements							HM
Light Perception							PL
No Light Perception							N PL

Note:
Enter the number of letters read in each row and enter the total in the appropriate box depending on whether read at 4 metres or 1metre. If there is no recording even at 1 metre, ircle one of the four visual acuities (FCCF/HM/PL/NPL) as appropriate.

2. LEFT EYE-UNAIDED

Four Metres							
						**Acuity** (Circle one)	**# of letters read**
First Row	E	m	Ǝ	E	ɯ	6/60	
Second Row	ɯ	Ǝ	E	ɯ	E	6/48	
Third Row	m	E	m	Ǝ	E	6/38	
Fourth Row	ɯ	E	Ǝ	E	Ǝ	6/30	
Fifth Row	m	Ǝ	ɯ	ɯ	m	6/24	
Sixth Row	E	ɯ	Ǝ	m	ɯ	6/19	
Seventh Row	ɯ	m	E	Ǝ	E	6/15	
Eighth Row	Ǝ	ɯ	Ǝ	ɯ	E	6/12	
Ninth Row	Ǝ	E	ɯ	E	m	6/9.5	
Tenth Row	ɯ	m	E	m	ɯ	6/7.5	
Eleventh Row	Ǝ	E	m	Ǝ	ɯ	6/6	
**Total E's read at 4 meters (actual number of letters + 30)**							
**One Metre**							
First Row	m	Ǝ	ɯ	E	Ǝ	1/40	
Second Row	ɯ	E	Ǝ	m	ɯ	1/32	
Third Row	m	E	ɯ	E	E	1/25	
Fourth Row	ɯ	Ǝ	m	Ǝ	E	1/20	
Fifth Row	E	Ǝ	ɯ	E	m	1/16	
Sixth Row	Ǝ	E	ɯ	m	Ǝ	1/12.5	
**Total E's read at 1 meter (actual number of letters)**							
Finger Counting close to face							FCCF
Hand Movements							HM
Light Perception							PL
No Light Perception							N PL

Note:
Enter the number of letters read in each row and enter the total in the appropriate box depending on whether read at 4 metres or 1metre. If there is no recording even at 1 metre. Circle one of the four visual acuities (FCCF/HM/PL/NPL) as appropriate.

3. RIGHT EYE –PRESENTING

**Four Metres**								
						**Acuity** (Circle one)	**# of letters read**	
First Row	E	m	Ǝ	E	ш	6/60		
Second Row	ш	Ǝ	E	ш	E	6/48		
Third Row	m	E	m	Ǝ	E	6/38		
Fourth Row	ш	E	Ǝ	E	Ǝ	6/30		
Fifth Row	m	Ǝ	ш	ш	m	6/24		
Sixth Row	E	ш	Ǝ	m	ш	6/19		
Seventh Row	ш	m	E	Ǝ	E	6/15		
Eighth Row	Ǝ	ш	Ǝ	ш	E	6/12		
Ninth Row	Ǝ	E	ш	E	m	6/9.5		
Tenth Row	ш	m	E	m	ш	6/7.5		
Eleventh Row	Ǝ	E	m	Ǝ	ш	6/6		
**Total E's read at 4 meters (actual number of letters + 30)**								
**One Metre**								
First Row	m	Ǝ	ш	E	Ǝ	1/40		
Second Row	ш	E	Ǝ	m	ш	1/32		
Third Row	m	E	ш	E	E	1/25		
Fourth Row	ш	Ǝ	m	Ǝ	E	1/20		
Fifth Row	E	Ǝ	ш	E	m	1/16		
Sixth Row	Ǝ	E	ш	m	Ǝ	1/12.5		
**Total E's read at 1 meter (actual number of letters)**								
Finger Counting close to face								FCCF
Hand Movements								HM
Light Perception								PL
No Light Perception								N PL

Note:
Enter the number of letters read in each row and enter the total in the appropriate box depending on whether read at 4 metres or 1metre. If there is no recording even at 1 metre, circle one of the four visual acuities (FCCF/HM/PL/NPL) as appropriate.

4 LEFT EYE-PRESENTING

Four Metres								
						**Acuity** (Circle one)	**# of letters read**	
First Row	E	m	∃	E	ш	6/60		
Second Row	ш	∃	E	ш	E	6/48		
Third Row	m	E	m	∃	E	6/38		
Fourth Row	ш	E	∃	E	∃	6/30		
Fifth Row	m	∃	ш	ш	m	6/24		
Sixth Row	E	ш	∃	m	ш	6/19		
Seventh Row	ш	m	E	∃	E	6/15		
Eighth Row	∃	ш	∃	ш	E	6/12		
Ninth Row	∃	E	ш	E	m	6/9.5		
Tenth Row	ш	m	E	m	ш	6/7.5		
Eleventh Row	∃	E	m	∃	ш	6/6		
**Total E's read at 4 meters (actual number of letters + 30)**								
**One Metre**								
First Row	m	∃	ш	E	∃	1/40		
Second Row	ш	E	∃	m	ш	1/32		
Third Row	m	E	ш	E	E	1/25		
Fourth Row	ш	∃	m	∃	E	1/20		
Fifth Row	E	∃	ш	E	m	1/16		
Sixth Row	∃	E	ш	m	∃	1/12.5		
**Total E's read at 1 meter (actual number of letters)**								
Finger Counting close to face								FCCF
Hand Movements								HM
Light Perception								PL
No Light Perception								N PL

Note:
Enter the number of letters read in each row and enter the total in the appropriate box depending on whether read at 4 metres or 1metre. If there is no recording even at 1 metre, circle one of the four visual acuities (FCCF/HM/PL/NPL) as appropriate.

5. RIGHT EYE – SUBJECTIVE ACCEPTANCE

**Four Metres**								
						**Acuity** (Circle one)	**# of letters read**	
First Row	E	m	Ǝ	E	ш	6/60		
Second Row	ш	Ǝ	E	ш	E	6/48		
Third Row	m	E	m	Ǝ	E	6/38		
Fourth Row	ш	E	Ǝ	E	Ǝ	6/30		
Fifth Row	m	Ǝ	ш	ш	m	6/24		
Sixth Row	E	ш	Ǝ	m	ш	6/19		
Seventh Row	ш	m	E	Ǝ	E	6/15		
Eighth Row	Ǝ	ш	Ǝ	ш	E	6/12		
Ninth Row	Ǝ	E	ш	E	m	6/9.5		
Tenth Row	ш	m	E	m	ш	6/7.5		
Eleventh Row	Ǝ	E	m	Ǝ	ш	6/6		
**Total E's read at 4 meters (actual number of letters + 30)**								
**One Metre**								
First Row	m	Ǝ	ш	E	Ǝ	1/40		
Second Row	ш	E	Ǝ	m	ш	1/32		
Third Row	m	E	ш	E	E	1/25		
Fourth Row	ш	Ǝ	m	Ǝ	E	1/20		
Fifth Row	E	Ǝ	ш	E	m	1/16		
Sixth Row	Ǝ	E	ш	m	Ǝ	1/12.5		
**Total E's read at 1 meter (actual number of letters)**								
Finger Counting close to face								FCCF
Hand Movements								HM
Light Perception								PL
No Light Perception								N PL

Note:
Enter the number of letters read in each row and enter the total in the appropriate box depending on whether read at 4 metres or 1metre. If there is no recording even at 1 metre, circle one of the four visual acuities (FCCF/HM/PL/NPL) as appropriate.

6. LEFT EYE – SUBJECTIVE ACCEPTANCE

**Four Metres**							
						**Acuity** (Circle one)	**# of letters read**
First Row	E	m	Ǝ	E	ш	6/60	
Second Row	ш	Ǝ	E	ш	E	6/48	
Third Row	m	E	m	Ǝ	E	6/38	
Fourth Row	ш	E	Ǝ	E	Ǝ	6/30	
Fifth Row	m	Ǝ	ш	ш	m	6/24	
Sixth Row	E	ш	Ǝ	m	ш	6/19	
Seventh Row	ш	m	E	Ǝ	E	6/15	
Eighth Row	Ǝ	ш	Ǝ	ш	E	6/12	
Ninth Row	Ǝ	E	ш	E	m	6/9.5	
Tenth Row	ш	m	E	m	ш	6/7.5	
Eleventh Row	Ǝ	E	m	Ǝ	ш	6/6	
**Total E’s read at 4 meters (actual number of letters + 30)**							
**One Metre**							
First Row	m	Ǝ	ш	E	Ǝ	1/40	
Second Row	ш	E	Ǝ	m	ш	1/32	
Third Row	m	E	ш	E	E	1/25	
Fourth Row	ш	Ǝ	m	Ǝ	E	1/20	
Fifth Row	E	Ǝ	ш	E	m	1/16	
Sixth Row	Ǝ	E	ш	m	Ǝ	1/12.5	
**Total E’s read at 1 meter (actual number of letters)**							

Finger Counting close to face	FCCF
Hand Movements	HM
Light Perception	PL
No Light Perception	N PL

Note:
Enter the number of letters read in each row and enter the total in the appropriate box depending on whether read at 4 metres or 1metre. If there is no recording even at 1 metre, circle one of the four visual acuities (FCCF/HM/PL/NPL) as appropriate.

7. NEAR VISION: Right Eye ***<u>Unaided</u>***

						**Acuity** (Circle one)	**# of letters read**
First Row	ш	m	Ǝ	ш	E	6/120	
Second Row	m	Ǝ	ш	E	ш	6/95	
Third Row	E	ш	m	ш	Ǝ	6/75	
Fourth Row	E	Ǝ	ш	E	m	6/60	
Fifth Row	Ǝ	m	E	ш	Ǝ	6/48	
Sixth Row	ш	E	m	Ǝ	ш	6/38	
Seventh Row	m	ш	Ǝ	m	E	6/30	
Eighth Row	Ǝ	E	ш	E	m	6/24	
Ninth Row	E	ш	m	ш	Ǝ	6/19	
Tenth Row	Ǝ	m	E	Ǝ	ш	6/15	
Eleventh Row	m	E	ш	m	Ǝ	6/12	
Twelfth Row	ш	Ǝ	m	ш	E	6/9.5	
Thirteenth Row	E	m	E	Ǝ	ш	6/7.5	
**Total Es read**							

8. NEAR VISION: Left Eye ***<u>Unaided</u>***

						**Acuity** (Circle one)	**# of letters read**
First Row	ш	m	Ǝ	ш	E	6/120	
Second Row	m	Ǝ	ш	E	ш	6/95	
Third Row	E	ш	m	ш	Ǝ	6/75	
Fourth Row	E	Ǝ	ш	E	m	6/60	
Fifth Row	Ǝ	m	E	ш	Ǝ	6/48	
Sixth Row	ш	E	m	Ǝ	ш	6/38	
Seventh Row	m	ш	Ǝ	m	E	6/30	
Eighth Row	Ǝ	E	ш	E	m	6/24	
Ninth Row	E	ш	m	ш	Ǝ	6/19	
Tenth Row	Ǝ	m	E	Ǝ	ш	6/15	
Eleventh Row	m	E	ш	m	Ǝ	6/12	
Twelfth Row	ш	Ǝ	m	ш	E	6/9.5	
Thirteenth Row	E	m	E	Ǝ	ш	6/7.5	
**Total Es read**							

Note: Enter the number of letters read in each row and enter the total in the appropriate box.

9. NEAR VISION: Right Eye *Presenting*

						**Acuity** (Circle one)	**# of letters read**
First Row	ш	m	Ǝ	ш	E	6/120	
Second Row	m	Ǝ	ш	E	ш	6/95	
Third Row	E	ш	m	ш	Ǝ	6/75	
Fourth Row	E	Ǝ	ш	E	m	6/60	
Fifth Row	Ǝ	m	E	ш	Ǝ	6/48	
Sixth Row	ш	E	m	Ǝ	ш	6/38	
Seventh Row	m	ш	Ǝ	m	E	6/30	
Eighth Row	Ǝ	E	ш	E	m	6/24	
Ninth Row	E	ш	m	ш	Ǝ	6/19	
Tenth Row	Ǝ	m	E	Ǝ	ш	6/15	
Eleventh Row	m	E	ш	m	Ǝ	6/12	
Twelfth Row	ш	Ǝ	m	ш	E	6/9.5	
Thirteenth Row	E	m	E	Ǝ	ш	6/7.5	
**Total Es read**							

10. NEAR VISION: Left Eye *Presenting*

						**Acuity** (Circle one)	**# of letters read**
First Row	ш	m	Ǝ	ш	E	6/120	
Second Row	m	Ǝ	ш	E	ш	6/95	
Third Row	E	ш	m	ш	Ǝ	6/75	
Fourth Row	E	Ǝ	ш	E	m	6/60	
Fifth Row	Ǝ	m	E	ш	Ǝ	6/48	
Sixth Row	ш	E	m	Ǝ	ш	6/38	
Seventh Row	m	ш	Ǝ	m	E	6/30	
Eighth Row	Ǝ	E	ш	E	m	6/24	
Ninth Row	E	ш	m	ш	Ǝ	6/19	
Tenth Row	Ǝ	m	E	Ǝ	ш	6/15	
Eleventh Row	m	E	ш	m	Ǝ	6/12	
Twelfth Row	ш	Ǝ	m	ш	E	6/9.5	
Thirteenth Row	E	m	E	Ǝ	ш	6/7.5	
**Total Es read**							

Note: Enter the number of letters read in each row and enter the total in the appropriate box.

11. NEAR VISION : Right Eye *Corrected*

						**Acuity** (Circle one)	**# of letters read**
First Row	ш	m	∃	ш	E	6/120	
Second Row	m	∃	ш	E	ш	6/95	
Third Row	E	ш	m	ш	∃	6/75	
Fourth Row	E	∃	ш	E	m	6/60	
Fifth Row	∃	m	E	ш	∃	6/48	
Sixth Row	ш	E	m	∃	ш	6/38	
Seventh Row	m	ш	∃	m	E	6/30	
Eighth Row	∃	E	ш	E	m	6/24	
Ninth Row	E	ш	m	ш	∃	6/19	
Tenth Row	∃	m	E	∃	ш	6/15	
Eleventh Row	m	E	ш	m	∃	6/12	
Twelfth Row	ш	∃	m	ш	E	6/9.5	
Thirteenth Row	E	m	E	∃	ш	6/7.5	
**Total Es read**							

12. NEAR VISION : Left Eye *Corrected*

						**Acuity** (Circle one)	**# of letters read**
First Row	ш	m	∃	ш	E	6/120	
Second Row	m	∃	ш	E	ш	6/95	
Third Row	E	ш	m	ш	∃	6/75	
Fourth Row	E	∃	ш	E	m	6/60	
Fifth Row	∃	m	E	ш	∃	6/48	
Sixth Row	ш	E	m	∃	ш	6/38	
Seventh Row	m	ш	∃	m	E	6/30	
Eighth Row	∃	E	ш	E	m	6/24	
Ninth Row	E	ш	m	ш	∃	6/19	
Tenth Row	∃	m	E	∃	ш	6/15	
Eleventh Row	m	E	ш	m	∃	6/12	
Twelfth Row	ш	∃	m	ш	E	6/9.5	
Thirteenth Row	E	m	E	∃	ш	6/7.5	
**Total Es read**							

Note: Enter the number of letters read in each row and enter the total in the appropriate box.

UVR FORM VI

CLINICAL EXAMINATION FORM for VKC (5-15 YEARS)

SECTION A: IDENTIFICATION DATA

IDENTIFICATION DATA	RESPONSE
Study Location	☐ Delhi=1, Guwahati=2, Chennai=3
Name of village	________________
Cluster Code	☐☐
House No.	☐☐☐
Person Number	☐☐
Person Unique ID No.	☐ - ☐☐ - ☐☐☐ - ☐☐<br>Location ID (1) + Cluster ID (2) + House No(3) + Person No (2)
Respondent Name	________________
Respondent Age<br>(in completed years)	☐☐
Gender	☐ Male = 1 ; Female = 2
Optometrist Code/Name	☐ ______________________________
Ophthalmologist Code/Name	☐ ______________________________
Place of Examination	☐ 1. Base hospital<br>2. Central field site<br>3. Home Examination
Date of Examination<br>(dd-mm-yyyy)	☐☐ - ☐☐ - ☐☐☐☐
Photograph taken	☐ Yes=1, No=2

SECTION B: ANTHROPOMETRY

WEIGHT

**State value in Kg up to one decimal point**	
**If weight measurement not possible, please state reason why (circle one):**<br>1. Participant refuses<br>2. Participant is chair/bed bound<br>3. Participant is too unsteady on feet<br>66. Other *Please specify:* ______________________________	

STANDING HEIGHT

**State value in cm up to one decimal point**	
**If standing height measurement not possible, please state reason why (circle one):**<br>1. Participant refuses<br>2. Participant is chair/bed bound<br>3. Participant is too unsteady on feet<br>4. Participant has a bent spine<br>66. Other *Please specify:* ______________________________	

MID UPPER ARM CIRCUMFERENCE

**State value (in cm) up to one decimal point**	
If circumference not taken, state the reason:	

C1: VISUAL ACUITY

**Wearing Glass (Yes=1, No=2)**					
**Vision**		**Right Eye**		**Left Eye**	
		**Acuity**	**Total 'E's read**	**Acuity**	**Total 'E's read**
**Unaided**	**Distance**				
	**Near**				
**Presenting (With usual glasses)**	**Distance**				
	**Near**				
**If vision not recorded, state the reason:**	**RE**______________________<br>**LE**______________________				

C2: SUBJECTIVE ACCEPTANCE

**Subjective Acceptance (Done/ Not Done)**						
**If Not Done, Give reason:** Corneal disease, Lenticular opacity, Patient Not Cooperative, Machine Error, Home Visit						
**Acceptance**		**Sphere**	**Cylinder**	**Axis**	**Acuity**	**Total Es read**
**Right Eye**	**Distance**					
	**Near**	**Add**	**D sph**			
**Left Eye**	**Distance**					
	**Near**	**Add**	**DSph**			

If undetermined give reasons RE________________ **LE**____________________

SECTION D: SLIT LAMP BIOMICROSCOPY EXAMINATON

☐ **Not Done, please explain:** ____

	**OD (Right)**				**OS (Left)**			
	None<br>0	Mild<br>+1	Moderate<br>+2	Severe<br>+3	None<br>0	Mild<br>+1	Moderate<br>+2	Severe<br>+3
a. Palpebral VKC	☐	☐	☐	☐	☐	☐	☐	☐
b. Limbal VKC	☐	☐	☐	☐	☐	☐	☐	☐
c. Bitot's spots	☐	☐	☐	☐	☐	☐	☐	☐
d. Corneal Staining	☐	☐	☐	☐	☐	☐	☐	☐
e.Corneal opacity	☐	☐	☐	☐	☐	☐	☐	☐
f. Shield Ulcer	☐	☐	☐	☐	☐	☐	☐	☐
g. Other Pathology	☐	☐	☐	☐	☐	☐	☐	☐
specify:								

PROTOCOL FOR VISUAL ACUITY TESTING

1. RIGHT EYE –UNAIDED

**Four Metres**							
						**Acuity** (Circle one)	**# of letters read**
First Row	E	m	∃	E	ш	6/60	
Second Row	ш	∃	E	ш	E	6/48	
Third Row	m	E	m	∃	E	6/38	
Fourth Row	ш	E	∃	E	∃	6/30	
Fifth Row	m	∃	ш	ш	m	6/24	
Sixth Row	E	ш	∃	m	ш	6/19	
Seventh Row	ш	m	E	∃	E	6/15	
Eighth Row	∃	ш	∃	ш	E	6/12	
Ninth Row	∃	E	ш	E	m	6/9.5	
Tenth Row	ш	m	E	m	ш	6/7.5	
Eleventh Row	∃	E	m	∃	ш	6/6	
						**Total E's read at 4 meters (actual number of letters + 30)**	
**One Metre**							
First Row	m	∃	ш	E	∃	1/40	
Second Row	ш	E	∃	m	ш	1/32	
Third Row	m	E	ш	E	E	1/25	
Fourth Row	ш	∃	m	∃	E	1/20	
Fifth Row	E	∃	ш	E	m	1/16	
Sixth Row	∃	E	ш	m	∃	1/12.5	
						**Total E's read at 1 meter (actual number of letters)**	
						Finger Counting close to face	FCCF
						Hand Movements	HM
						Light Perception	PL
						No Light Perception	N PL

Note:
Enter the number of letters read in each row and enter the total in the appropriate box depending on whether read at 4 metres or 1metre. If there is no recording even at 1 metre, ircle one of the four visual acuities (FCCF/HM/PL/NPL) as appropriate.

2. LEFT EYE-UNAIDED

**Four Metres**								
						**Acuity** (Circle one)	**# of letters read**	
First Row	E	m	Ǝ	E	ш	6/60		
Second Row	ш	Ǝ	E	ш	E	6/48		
Third Row	m	E	m	Ǝ	E	6/38		
Fourth Row	ш	E	Ǝ	E	Ǝ	6/30		
Fifth Row	m	Ǝ	ш	ш	m	6/24		
Sixth Row	E	ш	Ǝ	m	ш	6/19		
Seventh Row	ш	m	E	Ǝ	E	6/15		
Eighth Row	Ǝ	ш	Ǝ	ш	E	6/12		
Ninth Row	Ǝ	E	ш	E	m	6/9.5		
Tenth Row	ш	m	E	m	ш	6/7.5		
Eleventh Row	Ǝ	E	m	Ǝ	ш	6/6		
**Total E's read at 4 meters (actual number of letters + 30)**								
**One Metre**								
First Row	m	Ǝ	ш	E	Ǝ	1/40		
Second Row	ш	E	Ǝ	m	ш	1/32		
Third Row	m	E	ш	E	E	1/25		
Fourth Row	ш	Ǝ	m	Ǝ	E	1/20		
Fifth Row	E	Ǝ	ш	E	m	1/16		
Sixth Row	Ǝ	E	ш	m	Ǝ	1/12.5		
**Total E's read at 1 meter (actual number of letters)**								
Finger Counting close to face								FCCF
Hand Movements								HM
Light Perception								PL
No Light Perception								N PL

Note:
Enter the number of letters read in each row and enter the total in the appropriate box depending on whether read at 4 metres or 1metre. If there is no recording even at 1 metre. Circle one of the four visual acuities (FCCF/HM/PL/NPL) as appropriate.

3. RIGHT EYE –PRESENTING

**Four Metres**								
						**Acuity** (Circle one)	**# of letters read**	
First Row	E	m	Ǝ	E	ш	6/60		
Second Row	ш	Ǝ	E	ш	E	6/48		
Third Row	m	E	m	Ǝ	E	6/38		
Fourth Row	ш	E	Ǝ	E	Ǝ	6/30		
Fifth Row	m	Ǝ	ш	ш	m	6/24		
Sixth Row	E	ш	Ǝ	m	ш	6/19		
Seventh Row	ш	m	E	Ǝ	E	6/15		
Eighth Row	Ǝ	ш	Ǝ	ш	E	6/12		
Ninth Row	Ǝ	E	ш	E	m	6/9.5		
Tenth Row	ш	m	E	m	ш	6/7.5		
Eleventh Row	Ǝ	E	m	Ǝ	ш	6/6		
**Total E's read at 4 meters (actual number of letters + 30)**								
**One Metre**								
First Row	m	Ǝ	ш	E	Ǝ	1/40		
Second Row	ш	E	Ǝ	m	ш	1/32		
Third Row	m	E	ш	E	E	1/25		
Fourth Row	ш	Ǝ	m	Ǝ	E	1/20		
Fifth Row	E	Ǝ	ш	E	m	1/16		
Sixth Row	Ǝ	E	ш	m	Ǝ	1/12.5		
**Total E's read at 1 meter (actual number of letters)**								
Finger Counting close to face								FCCF
Hand Movements								HM
Light Perception								PL
No Light Perception								N PL

Note:
Enter the number of letters read in each row and enter the total in the appropriate box depending on whether read at 4 metres or 1metre. If there is no recording even at 1 metre, circle one of the four visual acuities (FCCF/HM/PL/NPL) as appropriate.

4. LEFT EYE-PRESENTING

**Four Metres**							
						**Acuity** (Circle one)	**# of letters read**
First Row	E	m	Ǝ	E	ш	6/60	
Second Row	ш	Ǝ	E	ш	E	6/48	
Third Row	m	E	m	Ǝ	E	6/38	
Fourth Row	ш	E	Ǝ	E	Ǝ	6/30	
Fifth Row	m	Ǝ	ш	ш	m	6/24	
Sixth Row	E	ш	Ǝ	m	ш	6/19	
Seventh Row	ш	m	E	Ǝ	E	6/15	
Eighth Row	Ǝ	ш	Ǝ	ш	E	6/12	
Ninth Row	Ǝ	E	ш	E	m	6/9.5	
Tenth Row	ш	m	E	m	ш	6/7.5	
Eleventh Row	Ǝ	E	m	Ǝ	ш	6/6	
**Total E's read at 4 meters (actual number of letters + 30)**							
**One Metre**							
First Row	m	Ǝ	ш	E	Ǝ	1/40	
Second Row	ш	E	Ǝ	m	ш	1/32	
Third Row	m	E	ш	E	E	1/25	
Fourth Row	ш	Ǝ	m	Ǝ	E	1/20	
Fifth Row	E	Ǝ	ш	E	m	1/16	
Sixth Row	Ǝ	E	ш	m	Ǝ	1/12.5	
**Total E's read at 1 meter (actual number of letters)**							
Finger Counting close to face							FCCF
Hand Movements							HM
Light Perception							PL
No Light Perception							N PL

Note:
Enter the number of letters read in each row and enter the total in the appropriate box depending on whether read at 4 metres or 1metre. If there is no recording even at 1 metre, circle one of the four visual acuities (FCCF/HM/PL/NPL) as appropriate.

5. RIGHT EYE – SUBJECTIVE ACCEPTANCE

**Four Metres**								
						**Acuity** (Circle one)	**# of letters read**	
First Row	E	m	Ǝ	E	ш	6/60		
Second Row	ш	Ǝ	E	ш	E	6/48		
Third Row	m	E	m	Ǝ	E	6/38		
Fourth Row	ш	E	Ǝ	E	Ǝ	6/30		
Fifth Row	m	Ǝ	ш	ш	m	6/24		
Sixth Row	E	ш	Ǝ	m	ш	6/19		
Seventh Row	ш	m	E	Ǝ	E	6/15		
Eighth Row	Ǝ	ш	Ǝ	ш	E	6/12		
Ninth Row	Ǝ	E	ш	E	m	6/9.5		
Tenth Row	ш	m	E	m	ш	6/7.5		
Eleventh Row	Ǝ	E	m	Ǝ	ш	6/6		
							**Total E's read at 4 meters (actual number of letters + 30)**	
**One Metre**								
First Row	m	Ǝ	ш	E	Ǝ	1/40		
Second Row	ш	E	Ǝ	m	ш	1/32		
Third Row	m	E	ш	E	E	1/25		
Fourth Row	ш	Ǝ	m	Ǝ	E	1/20		
Fifth Row	E	Ǝ	ш	E	m	1/16		
Sixth Row	Ǝ	E	ш	m	Ǝ	1/12.5		
							**Total E's read at 1 meter (actual number of letters)**	
							Finger Counting close to face	FCCF
							Hand Movements	HM
							Light Perception	PL
							No Light Perception	N PL

Note:
Enter the number of letters read in each row and enter the total in the appropriate box depending on whether read at 4 metres or 1metre. If there is no recording even at 1 metre, circle one of the four visual acuities (FCCF/HM/PL/NPL) as appropriate.

6. LEFT EYE – SUBJECTIVE ACCEPTANCE

**Four Metres**								
						**Acuity** (Circle one)	**# of letters read**	
First Row	E	m	Ǝ	E	ш	6/60		
Second Row	ш	Ǝ	E	ш	E	6/48		
Third Row	m	E	m	Ǝ	E	6/38		
Fourth Row	ш	E	Ǝ	E	Ǝ	6/30		
Fifth Row	m	Ǝ	ш	ш	m	6/24		
Sixth Row	E	ш	Ǝ	m	ш	6/19		
Seventh Row	ш	m	E	Ǝ	E	6/15		
Eighth Row	Ǝ	ш	Ǝ	ш	E	6/12		
Ninth Row	Ǝ	E	ш	E	m	6/9.5		
Tenth Row	ш	m	E	m	ш	6/7.5		
Eleventh Row	Ǝ	E	m	Ǝ	ш	6/6		
**Total E's read at 4 meters (actual number of letters + 30)**								
**One Metre**								
First Row	m	Ǝ	ш	E	Ǝ	1/40		
Second Row	ш	E	Ǝ	m	ш	1/32		
Third Row	m	E	ш	E	E	1/25		
Fourth Row	ш	Ǝ	m	Ǝ	E	1/20		
Fifth Row	E	Ǝ	ш	E	m	1/16		
Sixth Row	Ǝ	E	ш	m	Ǝ	1/12.5		
**Total E's read at 1 meter (actual number of letters)**								
Finger Counting close to face								FCCF
Hand Movements								HM
Light Perception								PL
No Light Perception								N PL

Note:
Enter the number of letters read in each row and enter the total in the appropriate box depending on whether read at 4 metres or 1metre. If there is no recording even at 1 metre, circle one of the four visual acuities (FCCF/HM/PL/NPL) as appropriate.

7. NEAR VISION: Right Eye ***Unaided***

						**Acuity** (Circle one)	**# of letters read**
First Row	ɯ	m	Ǝ	ɯ	E	6/120	
Second Row	m	Ǝ	ɯ	E	ɯ	6/95	
Third Row	E	ɯ	m	ɯ	Ǝ	6/75	
Fourth Row	E	Ǝ	ɯ	E	m	6/60	
Fifth Row	Ǝ	m	E	ɯ	Ǝ	6/48	
Sixth Row	ɯ	E	m	Ǝ	ɯ	6/38	
Seventh Row	m	ɯ	Ǝ	m	E	6/30	
Eighth Row	Ǝ	E	ɯ	E	m	6/24	
Ninth Row	E	ɯ	m	ɯ	Ǝ	6/19	
Tenth Row	Ǝ	m	E	Ǝ	ɯ	6/15	
Eleventh Row	m	E	ɯ	m	Ǝ	6/12	
Twelfth Row	ɯ	Ǝ	m	ɯ	E	6/9.5	
Thirteenth Row	E	m	E	Ǝ	ɯ	6/7.5	
**Total Es read**							

8. NEAR VISION: Left Eye ***Unaided***

						**Acuity** (Circle one)	**# of letters read**
First Row	ɯ	m	Ǝ	ɯ	E	6/120	
Second Row	m	Ǝ	ɯ	E	ɯ	6/95	
Third Row	E	ɯ	m	ɯ	Ǝ	6/75	
Fourth Row	E	Ǝ	ɯ	E	m	6/60	
Fifth Row	Ǝ	m	E	ɯ	Ǝ	6/48	
Sixth Row	ɯ	E	m	Ǝ	ɯ	6/38	
Seventh Row	m	ɯ	Ǝ	m	E	6/30	
Eighth Row	Ǝ	E	ɯ	E	m	6/24	
Ninth Row	E	ɯ	m	ɯ	Ǝ	6/19	
Tenth Row	Ǝ	m	E	Ǝ	ɯ	6/15	
Eleventh Row	m	E	ɯ	m	Ǝ	6/12	
Twelfth Row	ɯ	Ǝ	m	ɯ	E	6/9.5	
Thirteenth Row	E	m	E	Ǝ	ɯ	6/7.5	
**Total Es read**							

Note: Enter the number of letters read in each row and enter the total in the appropriate box.

9. NEAR VISION: Right Eye *Presenting*

						**Acuity** (Circle one)	**# of letters read**
First Row	ɯ	m	Ǝ	ɯ	E	6/120	
Second Row	m	Ǝ	ɯ	E	ɯ	6/95	
Third Row	E	ɯ	m	ɯ	Ǝ	6/75	
Fourth Row	E	Ǝ	ɯ	E	m	6/60	
Fifth Row	Ǝ	m	E	ɯ	Ǝ	6/48	
Sixth Row	ɯ	E	m	Ǝ	ɯ	6/38	
Seventh Row	m	ɯ	Ǝ	m	E	6/30	
Eighth Row	Ǝ	E	ɯ	E	m	6/24	
Ninth Row	E	ɯ	m	ɯ	Ǝ	6/19	
Tenth Row	Ǝ	m	E	Ǝ	ɯ	6/15	
Eleventh Row	m	E	ɯ	m	Ǝ	6/12	
Twelfth Row	ɯ	Ǝ	m	ɯ	E	6/9.5	
Thirteenth Row	E	m	E	Ǝ	ɯ	6/7.5	
**Total Es read**							

10. NEAR VISION: Left Eye *Presenting*

						**Acuity** (Circle one)	**# of letters read**
First Row	ɯ	m	Ǝ	ɯ	E	6/120	
Second Row	m	Ǝ	ɯ	E	ɯ	6/95	
Third Row	E	ɯ	m	ɯ	Ǝ	6/75	
Fourth Row	E	Ǝ	ɯ	E	m	6/60	
Fifth Row	Ǝ	m	E	ɯ	Ǝ	6/48	
Sixth Row	ɯ	E	m	Ǝ	ɯ	6/38	
Seventh Row	m	ɯ	Ǝ	m	E	6/30	
Eighth Row	Ǝ	E	ɯ	E	m	6/24	
Ninth Row	E	ɯ	m	ɯ	Ǝ	6/19	
Tenth Row	Ǝ	m	E	Ǝ	ɯ	6/15	
Eleventh Row	m	E	ɯ	m	Ǝ	6/12	
Twelfth Row	ɯ	Ǝ	m	ɯ	E	6/9.5	
Thirteenth Row	E	m	E	Ǝ	ɯ	6/7.5	
**Total Es read**							

Note: Enter the number of letters read in each row and enter the total in the appropriate box.

11. NEAR VISION : Right Eye *Corrected*

						**Acuity** (Circle one)	**# of letters read**
First Row	ш	m	Ǝ	ш	E	6/120	
Second Row	m	Ǝ	ш	E	ш	6/95	
Third Row	E	ш	m	ш	Ǝ	6/75	
Fourth Row	E	Ǝ	ш	E	m	6/60	
Fifth Row	Ǝ	m	E	ш	Ǝ	6/48	
Sixth Row	ш	E	m	Ǝ	ш	6/38	
Seventh Row	m	ш	Ǝ	m	E	6/30	
Eighth Row	Ǝ	E	ш	E	m	6/24	
Ninth Row	E	ш	m	ш	Ǝ	6/19	
Tenth Row	Ǝ	m	E	Ǝ	ш	6/15	
Eleventh Row	m	E	ш	m	Ǝ	6/12	
Twelfth Row	ш	Ǝ	m	ш	E	6/9.5	
Thirteenth Row	E	m	E	Ǝ	ш	6/7.5	
**Total Es read**							

12. NEAR VISION : Left Eye *Corrected*

						**Acuity** (Circle one)	**# of letters read**
First Row	ш	m	Ǝ	ш	E	6/120	
Second Row	m	Ǝ	ш	E	ш	6/95	
Third Row	E	ш	m	ш	Ǝ	6/75	
Fourth Row	E	Ǝ	ш	E	m	6/60	
Fifth Row	Ǝ	m	E	ш	Ǝ	6/48	
Sixth Row	ш	E	m	Ǝ	ш	6/38	
Seventh Row	m	ш	Ǝ	m	E	6/30	
Eighth Row	Ǝ	E	ш	E	m	6/24	
Ninth Row	E	ш	m	ш	Ǝ	6/19	
Tenth Row	Ǝ	m	E	Ǝ	ш	6/15	
Eleventh Row	m	E	ш	m	Ǝ	6/12	
Twelfth Row	ш	Ǝ	m	ш	E	6/9.5	
Thirteenth Row	E	m	E	Ǝ	ш	6/7.5	
**Total Es read**							

Note: Enter the number of letters read in each row and enter the total in the appropriate box.
