# Supplementary material for: Association of cataract and sun exposure in geographically diverse populations of India: The CASE study. First Report of the ICMR-EYE SEE Study Group
Source: PLoS One. 2020 Jan 23;15(1):e0227868. doi: 10.1371/journal.pone.0227868 (PMC6977762; doi:10.1371/journal.pone.0227868)
Supplement: S2 File — The study dataset in Stata and spreadsheet formats, codebooks, analysis commands, output logs and instructions for using the dataset. (ZIP) [file pone.0227868.s002.zip › LOCSeyePattern - Codebook.pdf]

Statistics/Data Analysis

```
      name:  <unnamed>
       log:  F:\Dropbox\VIVEK\paper\UVR Ctaract\PlOS Submission\R1\dataR1\UVR-cataract
> -analysis\LOCSeyePattern - Codebook.smcl
  log type:  smcl
 opened on:   6 Jan 2020, 12:50:17

1 . codebook

------------------------------------------------------------------------------
unique_id                                                          (unlabeled)
------------------------------------------------------------------------------

                  type:  numeric (long)

                 range:  [10100101,33509702]          units:  1
         unique values:  8,846                    missing .:  0/17,692

                  mean:   2.1e+07
              std. dev:   8.1e+06

           percentiles:        10%       25%       50%       75%       90%
                           1.1e+07   1.3e+07   2.1e+07   3.1e+07   3.2e+07

------------------------------------------------------------------------------
eye                                                                (unlabeled)
------------------------------------------------------------------------------

                  type:  numeric (byte)

                 range:  [1,2]                        units:  1
         unique values:  2                        missing .:  0/17,692

            tabulation:  Freq.  Value
                         8,846  1
                         8,846  2

------------------------------------------------------------------------------
study_location                                                     (unlabeled)
------------------------------------------------------------------------------

                  type:  numeric (byte)
                 label:  locationlb

                 range:  [1,3]                        units:  1
         unique values:  3                        missing .:  0/17,692

            tabulation:  Freq.   Numeric  Label
                         6,340         1  Delhi(1)
                         6,170         2  Guwahati(2)
                         5,182         3  Prakasam(3)

------------------------------------------------------------------------------
agecat                                                          RECODE of age
------------------------------------------------------------------------------

                  type:  numeric (int)
                 label:  agecatlb

                 range:  [4,7]                        units:  1
         unique values:  4                        missing .:  0/17,692

            tabulation:  Freq.   Numeric  Label
                         7,942         4  40-49 years
                         4,638         5  50-59 years
                         3,400         6  60-69 years
                         1,712         7  70+ years

------------------------------------------------------------------------------
gender                                                             (unlabeled)
------------------------------------------------------------------------------
```

```
                  type:  numeric (int)
                 label:  genderlb

                 range:  [1,2]                        units:  1
         unique values:  2                        missing .:  0/17,692

            tabulation:  Freq.   Numeric  Label
                         8,156         1  Male
                         9,536         2  Female
```

---

age **(unlabeled)**

```
                  type:  numeric (int)

                 range:  [40,99]                      units:  1
         unique values:  57                       missing .:  0/17,692

                  mean:    52.979
              std. dev:   10.6275

           percentiles:        10%       25%       50%       75%       90%
                                41        45        50        60        69
```

---

indoor16_kitchen **(unlabeled)**

```
                  type:  numeric (byte)
                 label:  ynlb2, but 1 nonmissing value is not labeled

                 range:  [0,1]                        units:  1
         unique values:  2                        missing .:  0/17,692

            tabulation:  Freq.   Numeric  Label
                         5,072         0
                        12,620         1  Yes
```

---

smoke20 **(unlabeled)**

```
                  type:  numeric (byte)
                 label:  ynlb2, but 1 nonmissing value is not labeled

                 range:  [0,1]                        units:  1
         unique values:  2                        missing .:  10/17,692

            tabulation:  Freq.   Numeric  Label
                        11,350         0
                         6,332         1  Yes
                            10         .
```

---

educat2 **RECODE of education**

```
                  type:  numeric (int)
                 label:  educat2, but 1 nonmissing value is not labeled

                 range:  [0,99]                       units:  1
         unique values:  7                        missing .:  0/17,692
```

```
            tabulation:  Freq.   Numeric  Label
                         8,738         0  Illiterate
                         3,354         1  Can read & write
                         1,774         2  Middle School (6-8)
                         3,294         3  High School (9-12)
                           512         4  Graduation
                             2         5  Others
                            18        99
```

---

workTypePrCat **(unlabeled)**

```
                  type:  numeric (float)
                 label:  workTypelb

                 range:  [1,2]                        units:  1
         unique values:  2                        missing .:  24/17,692

            tabulation:  Freq.   Numeric  Label
                         2,704         1  Indoor work
                        14,964         2  Outdoor work
                            24         .
```

---

tExposureQuantilesAll **5 quantiles of tExposure**

```
                  type:  numeric (byte)

                 range:  [1,5]                        units:  1
         unique values:  5                        missing .:  6/17,692

            tabulation:  Freq.  Value
                         3,728  1
                         3,740  2
                         3,644  3
                         3,554  4
                         3,020  5
                             6  .
```

---

cort_locs **(unlabeled)**

```
                  type:  numeric (float)
                 label:  cort, but 1 nonmissing value is not labeled

                 range:  [0,1]                        units:  1
         unique values:  2                        missing .:  593/17,692

            tabulation:  Freq.   Numeric  Label
                        16,118         0
                           981         1  Cort
                           593         .
```

---

nuc_locs **(unlabeled)**

```
                  type:  numeric (float)
                 label:  nuc, but 1 nonmissing value is not labeled

                 range:  [0,1]                        units:  1
         unique values:  2                        missing .:  593/17,692

            tabulation:  Freq.   Numeric  Label
                        13,808         0
                         3,291         1  Nuc
                           593         .
```

---

psc_locs **(unlabeled)**

```
                  type:  numeric (float)
                 label:  psc, but 1 nonmissing value is not labeled

                 range:  [0,1]                        units:  1
         unique values:  2                        missing .:  593/17,692

           tabulation:  Freq.   Numeric  Label
                       16,518         0
                          581         1  Psc
                          593         .
```

---

pva **(unlabeled)**

```
                  type:  numeric (float)
                 label:  pvaLe

                 range:  [1,5]                        units:  1
         unique values:  5                        missing .:  106/17,692

           tabulation:  Freq.   Numeric  Label
                       11,940         1  No VI
                        1,269         2  Mild VI
                        3,219         3  Moderate VI
                          243         4  Severe VI
                          915         5  Blind
                          106         .
```

---

bva **(unlabeled)**

```
                  type:  numeric (float)
                 label:  bva2Le

                 range:  [1,5]                        units:  1
         unique values:  5                        missing .:  106/17,692

           tabulation:  Freq.   Numeric  Label
                       15,021         1  No VI
                          531         2  Mild VI
                        1,111         3  Moderate VI
                          137         4  Severe VI
                          786         5  Blind
                          106         .
```

---

i **(unlabeled)**

```
                  type:  numeric (float)

                 range:  [1,8846]                     units:  1
         unique values:  8,846                    missing .:  0/17,692

                  mean:    4423.5
              std. dev:   2553.69

           percentiles:        10%       25%       50%       75%       90%
                               885      2212    4423.5      6635      7962
```

---

pattern **group(cort_locs nuc_locs psc_locs)**

```
                  type:  numeric (float)
                 label:  pattern

                 range:  [1,8]                        units:  1
         unique values:  8                        missing .:  593/17,692

            tabulation:  Freq.   Numeric  Label
                        13,624         1  0 0 0
                           101         2  0 0 Psc
                         2,226         3  0 Nuc 0
                           167         4  0 Nuc Psc
                            71         5  Cort 0 0
                            12         6  Cort 0 Psc
                           597         7  Cort Nuc 0
                           301         8  Cort Nuc Psc
                           593         .
```

---

cortAny **(unlabeled)**

```
                  type:  numeric (float)

                 range:  [0,1]                        units:  1
         unique values:  2                        missing .:  593/17,692

            tabulation:  Freq.  Value
                        16,118  0
                           981  1
                           593  .
```

---

nucAny **(unlabeled)**

```
                  type:  numeric (float)

                 range:  [0,1]                        units:  1
         unique values:  2                        missing .:  593/17,692

            tabulation:  Freq.  Value
                        13,808  0
                         3,291  1
                           593  .
```

---

pscAny **(unlabeled)**

```
                  type:  numeric (float)

                 range:  [0,1]                        units:  1
         unique values:  2                        missing .:  593/17,692

            tabulation:  Freq.  Value
                        16,518  0
                           581  1
                           593  .
```

```
2 . log close
      name:  <unnamed>
       log:  F:\Dropbox\VIVEK\paper\UVR Ctaract\PlOS Submission\R1\dataR1\UVR-cataract
> -analysis\LOCSeyePattern - Codebook.smcl
  log type:  smcl
 closed on:   6 Jan 2020, 12:50:27
```
