# Supplementary material for: Association of cataract and sun exposure in geographically diverse populations of India: The CASE study. First Report of the ICMR-EYE SEE Study Group
Source: PLoS One. 2020 Jan 23;15(1):e0227868. doi: 10.1371/journal.pone.0227868 (PMC6977762; doi:10.1371/journal.pone.0227868)
Supplement: S2 File — The study dataset in Stata and spreadsheet formats, codebooks, analysis commands, output logs and instructions for using the dataset. (ZIP) [file pone.0227868.s002.zip › Results.pdf]

Statistics/Data Analysis

```
      name:  <unnamed>
       log:  F:\Dropbox\VIVEK\paper\UVR Ctaract\PlOS Submission\R1\dataR1\UVR-cataract
> -analysis\ 6 Jan 2020_combinedUVR40+_6.smcl
  log type:  smcl
 opened on:   6 Jan 2020, 12:59:44

1 .
2 . // Table 1: Demographic characteristics of study participants examined for the study
  >
3 . // Age
4 . bysort study_location: ci mean age

-> study_location = Delhi(1)

    Variable |        Obs        Mean    Std. Err.       [95% Conf. Interval]
-------------+---------------------------------------------------------------
         age |      3,595    55.34604    .2016998        54.95058    55.74149

-> study_location = Guwahati(2)

    Variable |        Obs        Mean    Std. Err.       [95% Conf. Interval]
-------------+---------------------------------------------------------------
         age |      3,231    53.39152    .2024728        52.99453    53.78851

-> study_location = Prakasam(3)

    Variable |        Obs        Mean    Std. Err.       [95% Conf. Interval]
-------------+---------------------------------------------------------------
         age |      2,909    54.56824    .2070982        54.16216    54.97431

5 . ci mean age

    Variable |        Obs        Mean    Std. Err.       [95% Conf. Interval]
-------------+---------------------------------------------------------------
         age |      9,735    54.46492    .1181515        54.23332    54.69652

6 . bysort study_location: tabstat age, stat(median min max)

-> study_location = Delhi(1)

    variable |       p50       min       max
-------------+------------------------------
         age |        53        40        99
--------------------------------------------

-> study_location = Guwahati(2)

    variable |       p50       min       max
-------------+------------------------------
         age |        50        40        99
--------------------------------------------

-> study_location = Prakasam(3)

    variable |       p50       min       max
-------------+------------------------------
         age |        52        40        99
--------------------------------------------
```

7 . tabstat age, stat(median min max)

variable	p50	min	max
age	52	40	99

8 . // Gender
9 . bysort study_location: tab gender

-> study_location = Delhi(1)

gender	Freq.	Percent	Cum.
Male	1,614	44.90	44.90
Female	1,981	55.10	100.00
Total	3,595	100.00	

-> study_location = Guwahati(2)

gender	Freq.	Percent	Cum.
Male	1,491	46.15	46.15
Female	1,740	53.85	100.00
Total	3,231	100.00	

-> study_location = Prakasam(3)

gender	Freq.	Percent	Cum.
Male	1,321	45.41	45.41
Female	1,588	54.59	100.00
Total	2,909	100.00	

10. tab gender

gender	Freq.	Percent	Cum.
Male	4,426	45.46	45.46
Female	5,309	54.54	100.00
Total	9,735	100.00	

11. // Education
12. bysort study_location: tab educat2

-> study_location = Delhi(1)

RECODE of education	Freq.	Percent	Cum.
Illiterate	1,769	49.21	49.21
Can read & write	532	14.80	64.01
Middle School (6-8)	471	13.10	77.11
High School (9-12)	721	20.06	97.16
Graduation	102	2.84	100.00
Total	3,595	100.00	

-> study_location = Guwahati(2)

RECODE of education	Freq.	Percent	Cum.
Illiterate	1,306	40.42	40.42
Can read & write	779	24.11	64.53
Middle School (6-8)	294	9.10	73.63
High School (9-12)	742	22.97	96.60
Graduation	101	3.13	99.72
99	9	0.28	100.00
Total	3,231	100.00	

-> study_location = Prakasam(3)

RECODE of education	Freq.	Percent	Cum.
Illiterate	1,925	66.17	66.17
Can read & write	487	16.74	82.92
Middle School (6-8)	169	5.81	88.72
High School (9-12)	262	9.01	97.73
Graduation	65	2.23	99.97
Others	1	0.03	100.00
Total	2,909	100.00	

13. tab educat2

RECODE of education	Freq.	Percent	Cum.
Illiterate	5,000	51.36	51.36
Can read & write	1,798	18.47	69.83
Middle School (6-8)	934	9.59	79.42
High School (9-12)	1,725	17.72	97.14
Graduation	268	2.75	99.90
Others	1	0.01	99.91
99	9	0.09	100.00
Total	9,735	100.00	

14. // Occupation (Present)
15. bysort study_location: tab workTypePrCat

-> study_location = Delhi(1)

workTypePrCat	Freq.	Percent	Cum.
Indoor work	569	15.85	15.85
Outdoor work	3,021	84.15	100.00
Total	3,590	100.00	

-> study_location = Guwahati(2)

workTypePrCat	Freq.	Percent	Cum.
Indoor work	102	3.16	3.16
Outdoor work	3,121	96.84	100.00
Total	3,223	100.00	

-> study_location = Prakasam(3)

workTypePrCat	Freq.	Percent	Cum.
Indoor work	1,062	36.51	36.51
Outdoor work	1,847	63.49	100.00
Total	2,909	100.00	

```
16. tab workTypePrCat
```

workTypePrCat	Freq.	Percent	Cum.
Indoor work	1,733	17.83	17.83
Outdoor work	7,989	82.17	100.00
Total	9,722	100.00	

```
17. // Lifetime cumulative effective sun exposure
18. bysort study_location: tabstat tExposure, stat(p50 min max)
```

-> study_location = Delhi(1)

variable	p50	min	max
tExposure	114140.6	7305	314104

-> study_location = Guwahati(2)

variable	p50	min	max
tExposure	72759.63	7305	223763.1

-> study_location = Prakasam(3)

variable	p50	min	max
tExposure	109889.1	7305	252183.2

```
19. tabstat tExposure, stat(p50 min max)
```

variable	p50	min	max
tExposure	96062.57	7305	314104

```
20.
21. // Diabetes
22. bysort study_location: tab sugar200
```

-> study_location = Delhi(1)

sugar200	Freq.	Percent	Cum.
0	3,366	94.23	94.23
1	206	5.77	100.00
Total	3,572	100.00	

-> study_location = Guwahati(2)

sugar200	Freq.	Percent	Cum.
0	2,995	94.75	94.75
1	166	5.25	100.00
Total	3,161	100.00	

-> study_location = Prakasam(3)

sugar200	Freq.	Percent	Cum.
0	2,381	83.81	83.81
1	460	16.19	100.00
Total	2,841	100.00	

23. tab sugar200 study_location, col chi

Key
frequency
column percentage

	study_location			
sugar200	Delhi(1)	Guwahati(	Prakasam(	Total
0	3,366<br>94.23	2,995<br>94.75	2,381<br>83.81	8,742<br>91.31
1	206<br>5.77	166<br>5.25	460<br>16.19	832<br>8.69
Total	3,572<br>100.00	3,161<br>100.00	2,841<br>100.00	9,574<br>100.00

Pearson chi2(2) = 287.0320   Pr = 0.000

24.
25. // Vision Status
26. bysort study_location: tab pvaBe

-> study_location = Delhi(1)

pvaBe	Freq.	Percent	Cum.
1	2,480	69.04	69.04
2	308	8.57	77.62
3	695	19.35	96.97
4	32	0.89	97.86
5	77	2.14	100.00
Total	3,592	100.00	

-> study_location = Guwahati(2)

pvaBe	Freq.	Percent	Cum.
1	2,278	70.75	70.75
2	188	5.84	76.58
3	470	14.60	91.18
4	51	1.58	92.76
5	233	7.24	100.00
Total	3,220	100.00	

-> study_location = Prakasam(3)

pvaBe	Freq.	Percent	Cum.
1	2,002	69.90	69.90
2	258	9.01	78.91
3	544	18.99	97.91
4	29	1.01	98.92
5	31	1.08	100.00
Total	2,864	100.00	

27. tab pvaBe study_location, col chi

Key
frequency<br>*column percentage*

pvaBe	study_location<br>Delhi(1)	Guwahati(	Prakasam(	Total
1	2,480<br>69.04	2,278<br>70.75	2,002<br>69.90	6,760<br>69.86
2	308<br>8.57	188<br>5.84	258<br>9.01	754<br>7.79
3	695<br>19.35	470<br>14.60	544<br>18.99	1,709<br>17.66
4	32<br>0.89	51<br>1.58	29<br>1.01	112<br>1.16
5	77<br>2.14	233<br>7.24	31<br>1.08	341<br>3.52
Total	3,592<br>100.00	3,220<br>100.00	2,864<br>100.00	9,676<br>100.00

Pearson chi2(**8**) = **252.0816** Pr = **0.000**

28.
29.
30. // Table 2: Demographic details classified gender wise
31. for var anyCataract cort_prev nuc_prev psc_prev: tab X study_location, col nokey

-> tab anyCataract study_location, col nokey

anyCataract	study_location<br>Delhi(1)	Guwahati(	Prakasam(	Total
0	2,443<br>68.11	2,368<br>73.45	1,674<br>57.62	6,485<br>66.75
1	1,144<br>31.89	856<br>26.55	1,231<br>42.38	3,231<br>33.25
Total	3,587<br>100.00	3,224<br>100.00	2,905<br>100.00	9,716<br>100.00

-> tab cort_prev study_location, col nokey

cort_prev	Delhi(1)	Guwahati(	Prakasam(	Total
	study_location			
NormOrNotcort	2,819<br>88.93	2,842<br>92.12	2,571<br>99.23	8,232<br>93.06
cort	351<br>11.07	243<br>7.88	20<br>0.77	614<br>6.94
Total	3,170<br>100.00	3,085<br>100.00	2,591<br>100.00	8,846<br>100.00

-> tab nuc_prev study_location, col nokey

nuc_prev	Delhi(1)	Guwahati(	Prakasam(	Total
	study_location			
NormOrNotnuc	2,572<br>81.14	2,465<br>79.90	1,877<br>72.44	6,914<br>78.16
nuc	598<br>18.86	620<br>20.10	714<br>27.56	1,932<br>21.84
Total	3,170<br>100.00	3,085<br>100.00	2,591<br>100.00	8,846<br>100.00

-> tab psc_prev study_location, col nokey

psc_prev	Delhi(1)	Guwahati(	Prakasam(	Total
	study_location			
NormOrNotpsc	2,846<br>89.78	3,031<br>98.25	2,558<br>98.73	8,435<br>95.35
psc	324<br>10.22	54<br>1.75	33<br>1.27	411<br>4.65
Total	3,170<br>100.00	3,085<br>100.00	2,591<br>100.00	8,846<br>100.00

```
32.
33.
34. // Table 3: Prevalence of cataract in the presence of various risk factors
35. for var study_location agecat gender workTypePrCat ///
  >         educat2 smoke20 indoor16_kitchen tExposureQuantilesAll sugar200 pvaBe bvaBe:
  >  tab X anyCataract, row nokey chi
```

-> tab study_location anyCataract, row nokey chi

study_location	0	1	Total
	anyCataract		
Delhi(1)	2,443<br>68.11	1,144<br>31.89	3,587<br>100.00
Guwahati(2)	2,368<br>73.45	856<br>26.55	3,224<br>100.00
Prakasam(3)	1,674<br>57.62	1,231<br>42.38	2,905<br>100.00
Total	6,485<br>66.75	3,231<br>33.25	9,716<br>100.00

Pearson chi2(**2**) = **177.1463**   Pr = **0.000**

-> tab agecat anyCataract, row nokey chi

RECODE of age	anyCataract 0	1	Total
40-49 years	**3,738**<br>**93.54**	**258**<br>**6.46**	**3,996**<br>**100.00**
50-59 years	**1,826**<br>**75.05**	**607**<br>**24.95**	**2,433**<br>**100.00**
60-69 years	**791**<br>**40.01**	**1,186**<br>**59.99**	**1,977**<br>**100.00**
70+ years	**130**<br>**9.92**	**1,180**<br>**90.08**	**1,310**<br>**100.00**
Total	**6,485**<br>**66.75**	**3,231**<br>**33.25**	**9,716**<br>**100.00**

Pearson chi2(**3**) = **3.9e+03**   Pr = **0.000**

-> tab gender anyCataract, row nokey chi

gender	anyCataract 0	1	Total
Male	**2,990**<br>**67.71**	**1,426**<br>**32.29**	**4,416**<br>**100.00**
Female	**3,495**<br>**65.94**	**1,805**<br>**34.06**	**5,300**<br>**100.00**
Total	**6,485**<br>**66.75**	**3,231**<br>**33.25**	**9,716**<br>**100.00**

Pearson chi2(**1**) = **3.3807**   Pr = **0.066**

-> tab workTypePrCat anyCataract, row nokey chi

workTypePrCat	anyCataract 0	1	Total
Indoor work	**644**<br>**37.29**	**1,083**<br>**62.71**	**1,727**<br>**100.00**
Outdoor work	**5,833**<br>**73.13**	**2,143**<br>**26.87**	**7,976**<br>**100.00**
Total	**6,477**<br>**66.75**	**3,226**<br>**33.25**	**9,703**<br>**100.00**

Pearson chi2(**1**) = **821.7218**   Pr = **0.000**

-> tab educat2 anyCataract, row nokey chi

RECODE of education	anyCataract 0	1	Total
Illiterate	**2,815**<br>**56.49**	**2,168**<br>**43.51**	**4,983**<br>**100.00**
Can read & write	**1,288**<br>**71.64**	**510**<br>**28.36**	**1,798**<br>**100.00**
Middle School (6-8)	**727**<br>**77.84**	**207**<br>**22.16**	**934**<br>**100.00**
High School (9-12)	**1,411**<br>**81.89**	**312**<br>**18.11**	**1,723**<br>**100.00**
Graduation	**235**<br>**87.69**	**33**<br>**12.31**	**268**<br>**100.00**

Others	1<br>100.00	0<br>0.00	1<br>100.00
99	8<br>88.89	1<br>11.11	9<br>100.00
Total	6,485<br>66.75	3,231<br>33.25	9,716<br>100.00

Pearson chi2(**6**) = **540.6879**   Pr = **0.000**

-> tab smoke20 anyCataract, row nokey chi

smoke20	anyCataract<br>0	1	Total
0	4,280<br>69.76	1,855<br>30.24	6,135<br>100.00
Yes	2,203<br>61.62	1,372<br>38.38	3,575<br>100.00
Total	6,483<br>66.77	3,227<br>33.23	9,710<br>100.00

Pearson chi2(**1**) = **67.4714**   Pr = **0.000**

-> tab indoor16_kitchen anyCataract, row nokey chi

indoor16_k<br>itchen	anyCataract<br>0	1	Total
0	1,817<br>65.06	976<br>34.94	2,793<br>100.00
Yes	4,668<br>67.43	2,255<br>32.57	6,923<br>100.00
Total	6,485<br>66.75	3,231<br>33.25	9,716<br>100.00

Pearson chi2(**1**) = **5.0444**   Pr = **0.025**

-> tab tExposureQuantilesAll anyCataract, row nokey chi

5<br>quantiles<br>of<br>tExposure	anyCataract<br>0	1	Total
1	1,621<br>83.34	324<br>16.66	1,945<br>100.00
2	1,573<br>80.96	370<br>19.04	1,943<br>100.00
3	1,413<br>72.72	530<br>27.28	1,943<br>100.00
4	1,225<br>63.08	717<br>36.92	1,942<br>100.00
5	651<br>33.59	1,287<br>66.41	1,938<br>100.00
Total	6,483<br>66.76	3,228<br>33.24	9,711<br>100.00

Pearson chi2(**4**) = **1.4e+03**   Pr = **0.000**

-> tab sugar200 anyCataract, row nokey chi

sugar200	anyCataract 0	1	Total
0	5,885<br>67.43	2,842<br>32.57	8,727<br>100.00
1	491<br>59.01	341<br>40.99	832<br>100.00
Total	6,376<br>66.70	3,183<br>33.30	9,559<br>100.00

Pearson chi2(**1**) = **24.2459** Pr = **0.000**

-> tab pvaBe anyCataract, row nokey chi

pvaBe	anyCataract 0	1	Total
1	5,628<br>83.28	1,130<br>16.72	6,758<br>100.00
2	361<br>47.88	393<br>52.12	754<br>100.00
3	464<br>27.23	1,240<br>72.77	1,704<br>100.00
4	5<br>4.46	107<br>95.54	112<br>100.00
5	12<br>3.61	320<br>96.39	332<br>100.00
Total	6,470<br>66.98	3,190<br>33.02	9,660<br>100.00

Pearson chi2(**4**) = **3.0e+03** Pr = **0.000**

-> tab bvaBe anyCataract, row nokey chi

bvaBe	anyCataract 0	1	Total
1	6,380<br>75.23	2,101<br>24.77	8,481<br>100.00
2	41<br>13.90	254<br>86.10	295<br>100.00
3	40<br>7.38	502<br>92.62	542<br>100.00
4	2<br>2.78	70<br>97.22	72<br>100.00
5	7<br>2.59	263<br>97.41	270<br>100.00
Total	6,470<br>66.98	3,190<br>33.02	9,660<br>100.00

Pearson chi2(**4**) = **2.1e+03** Pr = **0.000**

```
36.
37.
38. ************************** CLINICAL ANY CATARACT
39. tab anyCataract
```

anyCataract	Freq.	Percent	Cum.
0	6,485	66.75	66.75
1	3,231	33.25	100.00
Total	9,716	100.00	

```
40. tab anyCataract study_location, col nokey chi
```

anyCataract	study_location			
	Delhi(1)	Guwahati(	Prakasam(	Total
0	2,443	2,368	1,674	6,485
	68.11	73.45	57.62	66.75
1	1,144	856	1,231	3,231
	31.89	26.55	42.38	33.25
Total	3,587	3,224	2,905	9,716
	100.00	100.00	100.00	100.00

Pearson chi2(**2**) = **177.1463**   Pr = **0.000**

```
41. tab1 beOp  oneEyeOp
```

-> tabulation of beOp

beOp	Freq.	Percent	Cum.
0	9,122	93.89	93.89
1	594	6.11	100.00
Total	9,716	100.00	

-> tabulation of oneEyeOp

oneEyeOp	Freq.	Percent	Cum.
0	9,087	93.53	93.53
1	629	6.47	100.00
Total	9,716	100.00	

```
42. tab1 beOp  oneEyeOp
```

-> tabulation of beOp

beOp	Freq.	Percent	Cum.
0	9,122	93.89	93.89
1	594	6.11	100.00
Total	9,716	100.00	

-> tabulation of oneEyeOp

oneEyeOp	Freq.	Percent	Cum.
0	9,087	93.53	93.53
1	629	6.47	100.00
Total	9,716	100.00	

43.
44. ************* LOCS Assessment in eyes
45. tab excludeLocs

excludeLocs	Freq.	Percent	Cum.
0	**8,846**	**91.05**	**91.05**
1	**870**	**8.95**	**100.00**
Total	**9,716**	**100.00**	

46. tab2 locsRe_cort locsLe_cort if anyCataract!=., mis

-> tabulation of locsRe_cort by locsLe_cort if anyCataract!=.

locsRe_cort	locsLe_cort 0	Cort	3.Adv	7.Tr.	8.Op	9.Mis	Total
0	**7,791**	**39**	**24**	**1**	**142**	**31**	**8,028**
Cort	**56**	**367**	**6**	**0**	**65**	**5**	**499**
3.Adv	**26**	**12**	**43**	**0**	**46**	**6**	**133**
7.Tr.	**3**	**0**	**1**	**0**	**2**	**0**	**6**
8.Op	**178**	**63**	**63**	**4**	**594**	**33**	**935**
9.Mis	**36**	**1**	**3**	**0**	**33**	**42**	**115**
Total	**8,090**	**482**	**140**	**5**	**882**	**117**	**9,716**

47. *** Type fo cataract
48. tab1 cort_locs_re cort_locs_le nuc_locs_re nuc_locs_le psc_locs_re psc_locs_le if
> anyCataract!=.

-> tabulation of cort_locs_re if anyCataract!=.

cort_locs_re	Freq.	Percent	Cum.
0	**8,028**	**94.15**	**94.15**
Cort	**499**	**5.85**	**100.00**
Total	**8,527**	**100.00**	

-> tabulation of cort_locs_le if anyCataract!=.

cort_locs_le	Freq.	Percent	Cum.
0	**8,090**	**94.38**	**94.38**
Cort	**482**	**5.62**	**100.00**
Total	**8,572**	**100.00**	

-> tabulation of nuc_locs_re if anyCataract!=.

nuc_locs_re	Freq.	Percent	Cum.
0	**6,893**	**80.84**	**80.84**
Nuc	**1,634**	**19.16**	**100.00**
Total	**8,527**	**100.00**	

-> tabulation of nuc_locs_le if anyCataract!=.

nuc_locs_le	Freq.	Percent	Cum.
0	6,915	80.67	80.67
Nuc	1,657	19.33	100.00
Total	8,572	100.00	

-> tabulation of psc_locs_re if anyCataract!=.

psc_locs_re	Freq.	Percent	Cum.
0	8,231	96.53	96.53
Psc	296	3.47	100.00
Total	8,527	100.00	

-> tabulation of psc_locs_le if anyCataract!=.

psc_locs_le	Freq.	Percent	Cum.
0	8,287	96.68	96.68
Psc	285	3.32	100.00
Total	8,572	100.00	

```
49. tab2 study_location cort_locs_re cort_locs_le nuc_locs_re nuc_locs_le  psc_locs_re
  > psc_locs_le if anyCataract!=. , firstonly row nokey chi
```

-> tabulation of study_location by cort_locs_re if anyCataract!=.

study_location	cort_locs_re 0	Cort	Total
Delhi(1)	2,761	285	3,046
	90.64	9.36	100.00
Guwahati(2)	2,806	202	3,008
	93.28	6.72	100.00
Prakasam(3)	2,461	12	2,473
	99.51	0.49	100.00
Total	8,028	499	8,527
	94.15	5.85	100.00

Pearson chi2(**2**) = **201.2515**   Pr = **0.000**

-> tabulation of study_location by cort_locs_le if anyCataract!=.

study_location	cort_locs_le 0	Cort	Total
Delhi(1)	2,784	261	3,045
	91.43	8.57	100.00
Guwahati(2)	2,824	203	3,027
	93.29	6.71	100.00
Prakasam(3)	2,482	18	2,500
	99.28	0.72	100.00
Total	8,090	482	8,572
	94.38	5.62	100.00

Pearson chi2(**2**) = **169.8238**   Pr = **0.000**

-> tabulation of study_location by nuc_locs_re if anyCataract!=.

study_location	nuc_locs_re 0	Nuc	Total
Delhi(1)	2,549<br>83.68	497<br>16.32	3,046<br>100.00
Guwahati(2)	2,463<br>81.88	545<br>18.12	3,008<br>100.00
Prakasam(3)	1,881<br>76.06	592<br>23.94	2,473<br>100.00
Total	6,893<br>80.84	1,634<br>19.16	8,527<br>100.00

Pearson chi2(**2**) = **54.4601** Pr = **0.000**

-> tabulation of study_location by nuc_locs_le if anyCataract!=.

study_location	nuc_locs_le 0	Nuc	Total
Delhi(1)	2,560<br>84.07	485<br>15.93	3,045<br>100.00
Guwahati(2)	2,473<br>81.70	554<br>18.30	3,027<br>100.00
Prakasam(3)	1,882<br>75.28	618<br>24.72	2,500<br>100.00
Total	6,915<br>80.67	1,657<br>19.33	8,572<br>100.00

Pearson chi2(**2**) = **71.2313** Pr = **0.000**

-> tabulation of study_location by psc_locs_re if anyCataract!=.

study_location	psc_locs_re 0	Psc	Total
Delhi(1)	2,811<br>92.28	235<br>7.72	3,046<br>100.00
Guwahati(2)	2,967<br>98.64	41<br>1.36	3,008<br>100.00
Prakasam(3)	2,453<br>99.19	20<br>0.81	2,473<br>100.00
Total	8,231<br>96.53	296<br>3.47	8,527<br>100.00

Pearson chi2(**2**) = **255.9309** Pr = **0.000**

-> tabulation of study_location by psc_locs_le if anyCataract!=.

study_location	psc_locs_le 0	Psc	Total
Delhi(1)	2,826<br>92.81	219<br>7.19	3,045<br>100.00
Guwahati(2)	2,981<br>98.48	46<br>1.52	3,027<br>100.00
Prakasam(3)	2,480<br>99.20	20<br>0.80	2,500<br>100.00
Total	8,287<br>96.68	285<br>3.32	8,572<br>100.00

Pearson chi2(**2**) = **221.9551**   Pr = **0.000**

```
50. tab2 sugar200           cort_locs_re cort_locs_le nuc_locs_re nuc_locs_le  psc_locs_
  > re  psc_locs_le if anyCataract!=. , firstonly row nokey chi
```

-> tabulation of sugar200 by cort_locs_re if anyCataract!=.

sugar200	cort_locs_re 0	Cort	Total
0	7,226<br>93.91	469<br>6.09	7,695<br>100.00
1	671<br>96.27	26<br>3.73	697<br>100.00
Total	7,897<br>94.10	495<br>5.90	8,392<br>100.00

Pearson chi2(**1**) = **6.4380**   Pr = **0.011**

-> tabulation of sugar200 by cort_locs_le if anyCataract!=.

sugar200	cort_locs_le 0	Cort	Total
0	7,285<br>94.24	445<br>5.76	7,730<br>100.00
1	671<br>95.72	30<br>4.28	701<br>100.00
Total	7,956<br>94.37	475<br>5.63	8,431<br>100.00

Pearson chi2(**1**) = **2.6379**   Pr = **0.104**

-> tabulation of sugar200 by nuc_locs_re if anyCataract!=.

sugar200	nuc_locs_re 0	Nuc	Total
0	6,241<br>81.10	1,454<br>18.90	7,695<br>100.00
1	541<br>77.62	156<br>22.38	697<br>100.00
Total	6,782<br>80.82	1,610<br>19.18	8,392<br>100.00

Pearson chi2(**1**) = **5.0100**   Pr = **0.025**

-> tabulation of sugar200 by nuc_locs_le if anyCataract!=.

sugar200	nuc_locs_le 0	Nuc	Total
0	6,266<br>81.06	1,464<br>18.94	7,730<br>100.00
1	536<br>76.46	165<br>23.54	701<br>100.00
Total	6,802<br>80.68	1,629<br>19.32	8,431<br>100.00

Pearson chi2(**1**) = **8.7191**   Pr = **0.003**

-> tabulation of sugar200 by psc_locs_re if anyCataract!=.

sugar200	psc_locs_re 0	Psc	Total
0	**7,420**<br>**96.43**	**275**<br>**3.57**	**7,695**<br>**100.00**
1	**676**<br>**96.99**	**21**<br>**3.01**	**697**<br>**100.00**
Total	**8,096**<br>**96.47**	**296**<br>**3.53**	**8,392**<br>**100.00**

Pearson chi2(**1**) =   **0.5908**   Pr = **0.442**

-> tabulation of sugar200 by psc_locs_le if anyCataract!=.

sugar200	psc_locs_le 0	Psc	Total
0	**7,465**<br>**96.57**	**265**<br>**3.43**	**7,730**<br>**100.00**
1	**684**<br>**97.57**	**17**<br>**2.43**	**701**<br>**100.00**
Total	**8,149**<br>**96.66**	**282**<br>**3.34**	**8,431**<br>**100.00**

Pearson chi2(**1**) =   **2.0004**   Pr = **0.157**

```
51.
52.
53. *** Pattern in eyes
54. use "LOCSeyePattern.dta", clear

55. merge m:1 unique_id using  "UVR-cataract-analysis.dta", keepusing(sugar200)
  (label locationlb already defined)
  (label workTypelb already defined)
  (label educat2 already defined)
  (label ynlb2 already defined)
  (label genderlb already defined)
  (label agecatlb already defined)
  (label cort already defined)
  (label nuc already defined)
  (label psc already defined)
```

Result	# of obs.	
not matched	**889**	
from master	**0**	(_merge==1)
from using	**889**	(_merge==2)
matched	**17,692**	(_merge==3)

```
56. keep if _merge==3
  (889 observations deleted)

57. drop _merge
```

58.
59. tab pattern

group(cort_l ocs nuc_locs psc_locs)	Freq.	Percent	Cum.
0 0 0	13,624	79.68	79.68
0 0 Psc	101	0.59	80.27
0 Nuc 0	2,226	13.02	93.29
0 Nuc Psc	167	0.98	94.26
Cort 0 0	71	0.42	94.68
Cort 0 Psc	12	0.07	94.75
Cort Nuc 0	597	3.49	98.24
Cort Nuc Psc	301	1.76	100.00
Total	17,099	100.00	

60. tab2 pattern study_location , col nokey chi

-> tabulation of pattern by study_location

group(cort_l ocs nuc_locs psc_locs)	study_location			
	Delhi(1)	Guwahati(	Prakasam(	Total
0 0 0	5,044<br>82.81	4,884<br>80.93	3,696<br>74.32	13,624<br>79.68
0 0 Psc	33<br>0.54	29<br>0.48	39<br>0.78	101<br>0.59
0 Nuc 0	335<br>5.50	683<br>11.32	1,208<br>24.29	2,226<br>13.02
0 Nuc Psc	133<br>2.18	34<br>0.56	0<br>0.00	167<br>0.98
Cort 0 0	21<br>0.34	23<br>0.38	27<br>0.54	71<br>0.42
Cort 0 Psc	11<br>0.18	0<br>0.00	1<br>0.02	12<br>0.07
Cort Nuc 0	237<br>3.89	358<br>5.93	2<br>0.04	597<br>3.49
Cort Nuc Psc	277<br>4.55	24<br>0.40	0<br>0.00	301<br>1.76
Total	6,091<br>100.00	6,035<br>100.00	4,973<br>100.00	17,099<br>100.00

Pearson chi2(**14**) = **1.7e+03** Pr = **0.000**

61. tab2 cort_locs eye

-> tabulation of cort_locs by eye

cort_locs	eye 1	2	Total
0	8,028	8,090	16,118
Cort	499	482	981
Total	8,527	8,572	17,099

62. tab2 nuc_locs eye

-> tabulation of nuc_locs by eye

nuc_locs	eye 1	2	Total
0	6,893	6,915	13,808
Nuc	1,634	1,657	3,291
Total	8,527	8,572	17,099

63. tab2 psc_locs eye

-> tabulation of psc_locs by eye

psc_locs	eye 1	2	Total
0	8,231	8,287	16,518
Psc	296	285	581
Total	8,527	8,572	17,099

64.
65. tab2 cortAny eye, col

-> tabulation of cortAny by eye

Key
frequency<br>*column percentage*

cortAny	eye 1	2	Total
0	8,028<br>94.15	8,090<br>94.38	16,118<br>94.26
1	499<br>5.85	482<br>5.62	981<br>5.74
Total	8,527<br>100.00	8,572<br>100.00	17,099<br>100.00

66. tab2 nucAny eye, col

-> tabulation of nucAny by eye

Key
frequency<br>*column percentage*

nucAny	eye 1	2	Total
0	6,893<br>80.84	6,915<br>80.67	13,808<br>80.75
1	1,634<br>19.16	1,657<br>19.33	3,291<br>19.25
Total	8,527<br>100.00	8,572<br>100.00	17,099<br>100.00

67. tab2 pscAny eye, col

-> tabulation of pscAny by eye

Key
frequency   *column percentage*

pscAny	eye 1	2	Total
0	**8,231** **96.53**	**8,287** **96.68**	**16,518** **96.60**
1	**296** **3.47**	**285** **3.32**	**581** **3.40**
Total	**8,527** **100.00**	**8,572** **100.00**	**17,099** **100.00**

68. tab2 cortAny study_location, col

-> tabulation of cortAny by study_location

Key
frequency   *column percentage*

cortAny	study_location Delhi(1)	Guwahati(	Prakasam(	Total
0	**5,545** **91.04**	**5,630** **93.29**	**4,943** **99.40**	**16,118** **94.26**
1	**546** **8.96**	**405** **6.71**	**30** **0.60**	**981** **5.74**
Total	**6,091** **100.00**	**6,035** **100.00**	**4,973** **100.00**	**17,099** **100.00**

69. tab2 nucAny study_location, col

-> tabulation of nucAny by study_location

Key
frequency   *column percentage*

nucAny	study_location Delhi(1)	Guwahati(	Prakasam(	Total
0	**5,109** **83.88**	**4,936** **81.79**	**3,763** **75.67**	**13,808** **80.75**
1	**982** **16.12**	**1,099** **18.21**	**1,210** **24.33**	**3,291** **19.25**
Total	**6,091** **100.00**	**6,035** **100.00**	**4,973** **100.00**	**17,099** **100.00**

```
70. tab2 pscAny study_location, col

  -> tabulation of pscAny by study_location
```

Key
frequency
column percentage

pscAny	study_location Delhi(1)	Guwahati(	Prakasam(	Total
0	**5,637**<br>**92.55**	**5,948**<br>**98.56**	**4,933**<br>**99.20**	**16,518**<br>**96.60**
1	**454**<br>**7.45**	**87**<br>**1.44**	**40**<br>**0.80**	**581**<br>**3.40**
Total	**6,091**<br>**100.00**	**6,035**<br>**100.00**	**4,973**<br>**100.00**	**17,099**<br>**100.00**

```
71.
72. * Diabetes and type of opcacity
73. tab2 cortAny sugar200, col chi

  -> tabulation of cortAny by sugar200
```

Key
frequency
column percentage

cortAny	sugar200 0	1	Total
0	**14,511**<br>**94.07**	**1,342**<br>**95.99**	**15,853**<br>**94.23**
1	**914**<br>**5.93**	**56**<br>**4.01**	**970**<br>**5.77**
Total	**15,425**<br>**100.00**	**1,398**<br>**100.00**	**16,823**<br>**100.00**

Pearson chi2(**1**) = **8.6942** Pr = **0.003**

```
74. tab2 nucAny sugar200, col  chi

  -> tabulation of nucAny by sugar200
```

Key
frequency
column percentage

nucAny	sugar200 0	1	Total
0	**12,507** **81.08**	**1,077** **77.04**	**13,584** **80.75**
1	**2,918** **18.92**	**321** **22.96**	**3,239** **19.25**
Total	**15,425** **100.00**	**1,398** **100.00**	**16,823** **100.00**

Pearson chi2(**1**) = **13.4842** Pr = **0.000**

75. tab2 pscAny sugar200, col chi

-> tabulation of pscAny by sugar200

Key
frequency *column percentage*

pscAny	sugar200 0	1	Total
0	**14,885** **96.50**	**1,360** **97.28**	**16,245** **96.56**
1	**540** **3.50**	**38** **2.72**	**578** **3.44**
Total	**15,425** **100.00**	**1,398** **100.00**	**16,823** **100.00**

Pearson chi2(**1**) = **2.3665** Pr = **0.124**

76.
77.
78. * Gender wise
79. bysort gender: tab2 pattern study_location if pattern !=. & pattern !=1, mis nokey
> chi

-> gender = Male

-> tabulation of pattern by study_location if pattern !=. & pattern !=1

group(cort_locs nuc_locs psc_locs)	study_location Delhi(1)	Guwahati(	Prakasam(	Total
0 0 Psc	**18**	**10**	**16**	**44**
0 Nuc 0	**157**	**325**	**564**	**1,046**
0 Nuc Psc	**61**	**14**	**0**	**75**
Cort 0 0	**8**	**11**	**17**	**36**
Cort 0 Psc	**5**	**0**	**0**	**5**
Cort Nuc 0	**105**	**149**	**2**	**256**
Cort Nuc Psc	**131**	**10**	**0**	**141**
Total	**485**	**519**	**599**	**1,603**

Pearson chi2(**12**) = **688.5459** Pr = **0.000**

-> gender = Female

-> tabulation of pattern by study_location if pattern !=. & pattern !=1

group(cort_locs nuc_locs psc_locs)	study_location Delhi(1)	Guwahati(	Prakasam(	Total
0 0 Psc	15	19	23	57
0 Nuc 0	178	358	644	1,180
0 Nuc Psc	72	20	0	92
Cort 0 0	13	12	10	35
Cort 0 Psc	6	0	1	7
Cort Nuc 0	132	209	0	341
Cort Nuc Psc	146	14	0	160
Total	562	632	678	1,872

Pearson chi2(**12**) = **818.3996**   Pr = **0.000**

80. bysort gender: tab2 pattern study_location if pattern !=. & pattern !=1, mis col nok
> ey nofreq chi

-> gender = Male

-> tabulation of pattern by study_location if pattern !=. & pattern !=1

group(cort_locs nuc_locs psc_locs)	study_location Delhi(1)	Guwahati(	Prakasam(	Total
0 0 Psc	3.71	1.93	2.67	2.74
0 Nuc 0	32.37	62.62	94.16	65.25
0 Nuc Psc	12.58	2.70	0.00	4.68
Cort 0 0	1.65	2.12	2.84	2.25
Cort 0 Psc	1.03	0.00	0.00	0.31
Cort Nuc 0	21.65	28.71	0.33	15.97
Cort Nuc Psc	27.01	1.93	0.00	8.80
Total	100.00	100.00	100.00	100.00

Pearson chi2(**12**) = **688.5459**   Pr = **0.000**

-> gender = Female

-> tabulation of pattern by study_location if pattern !=. & pattern !=1

group(cort_locs nuc_locs psc_locs)	study_location Delhi(1)	Guwahati(	Prakasam(	Total
0 0 Psc	2.67	3.01	3.39	3.04
0 Nuc 0	31.67	56.65	94.99	63.03
0 Nuc Psc	12.81	3.16	0.00	4.91
Cort 0 0	2.31	1.90	1.47	1.87
Cort 0 Psc	1.07	0.00	0.15	0.37
Cort Nuc 0	23.49	33.07	0.00	18.22
Cort Nuc Psc	25.98	2.22	0.00	8.55
Total	100.00	100.00	100.00	100.00

Pearson chi2(**12**) = **818.3996**   Pr = **0.000**

81. tab2 pattern gender if pattern !=. & pattern !=1, mis col nokey chi

-> tabulation of pattern by gender if pattern !=. & pattern !=1

group(cort_locs nuc_locs psc_locs)	gender Male	Female	Total
0 0 Psc	44<br>2.74	57<br>3.04	101<br>2.91
0 Nuc 0	1,046<br>65.25	1,180<br>63.03	2,226<br>64.06
0 Nuc Psc	75<br>4.68	92<br>4.91	167<br>4.81
Cort 0 0	36<br>2.25	35<br>1.87	71<br>2.04
Cort 0 Psc	5<br>0.31	7<br>0.37	12<br>0.35
Cort Nuc 0	256<br>15.97	341<br>18.22	597<br>17.18
Cort Nuc Psc	141<br>8.80	160<br>8.55	301<br>8.66
Total	1,603<br>100.00	1,872<br>100.00	3,475<br>100.00

Pearson chi2(**6**) = **4.3218** Pr = **0.633**

82. bysort study_location: tab2 pattern gender if pattern !=. & pattern !=1, col nokey
> chi

-> study_location = Delhi(1)

-> tabulation of pattern by gender if pattern !=. & pattern !=1

group(cort_locs nuc_locs psc_locs)	gender Male	Female	Total
0 0 Psc	18<br>3.71	15<br>2.67	33<br>3.15
0 Nuc 0	157<br>32.37	178<br>31.67	335<br>32.00
0 Nuc Psc	61<br>12.58	72<br>12.81	133<br>12.70
Cort 0 0	8<br>1.65	13<br>2.31	21<br>2.01
Cort 0 Psc	5<br>1.03	6<br>1.07	11<br>1.05
Cort Nuc 0	105<br>21.65	132<br>23.49	237<br>22.64
Cort Nuc Psc	131<br>27.01	146<br>25.98	277<br>26.46
Total	485<br>100.00	562<br>100.00	1,047<br>100.00

Pearson chi2(**6**) = **2.0166** Pr = **0.918**

-> study_location = Guwahati(2)

-> tabulation of pattern by gender if pattern !=. & pattern !=1

group(cort_locs nuc_locs psc_locs)	gender Male	Female	Total
0 0 Psc	10<br>1.93	19<br>3.01	29<br>2.52
0 Nuc 0	325<br>62.62	358<br>56.65	683<br>59.34
0 Nuc Psc	14<br>2.70	20<br>3.16	34<br>2.95
Cort 0 0	11<br>2.12	12<br>1.90	23<br>2.00
Cort Nuc 0	149<br>28.71	209<br>33.07	358<br>31.10
Cort Nuc Psc	10<br>1.93	14<br>2.22	24<br>2.09
Total	519<br>100.00	632<br>100.00	1,151<br>100.00

Pearson chi2(**5**) = **5.1684** Pr = **0.396**

-> study_location = Prakasam(3)

-> tabulation of pattern by gender if pattern !=. & pattern !=1

group(cort_locs nuc_locs psc_locs)	gender Male	Female	Total
0 0 Psc	16<br>2.67	23<br>3.39	39<br>3.05
0 Nuc 0	564<br>94.16	644<br>94.99	1,208<br>94.60
Cort 0 0	17<br>2.84	10<br>1.47	27<br>2.11
Cort 0 Psc	0<br>0.00	1<br>0.15	1<br>0.08
Cort Nuc 0	2<br>0.33	0<br>0.00	2<br>0.16
Total	599<br>100.00	678<br>100.00	1,277<br>100.00

Pearson chi2(**4**) = **6.5069** Pr = **0.164**

```
83.
84.
85. tab2 pattern sugar200 if pattern !=. & pattern !=1,  col nokey chi
```

-> tabulation of pattern by sugar200 if pattern !=. & pattern !=1

group(cort_locs nuc_locs psc_locs)	sugar200 0	1	Total
0 0 Psc	**89**<br>**2.89**	**12**<br>**3.53**	**101**<br>**2.95**
0 Nuc 0	**1,926**<br>**62.47**	**261**<br>**76.76**	**2,187**<br>**63.89**
0 Nuc Psc	**154**<br>**5.00**	**11**<br>**3.24**	**165**<br>**4.82**
Cort 0 0	**64**<br>**2.08**	**7**<br>**2.06**	**71**<br>**2.07**
Cort 0 Psc	**12**<br>**0.39**	**0**<br>**0.00**	**12**<br>**0.35**
Cort Nuc 0	**553**<br>**17.94**	**34**<br>**10.00**	**587**<br>**17.15**
Cort Nuc Psc	**285**<br>**9.24**	**15**<br>**4.41**	**300**<br>**8.76**
Total	**3,083**<br>**100.00**	**340**<br>**100.00**	**3,423**<br>**100.00**

Pearson chi2(**6**) = **32.9207** Pr = **0.000**

```
86. logistic sugar200 i.pattern
note: 6.pattern != 0 predicts failure perfectly
      6.pattern dropped and 12 obs not used
```

Logistic regression

Number of obs	=	**16,811**
LR chi2(**6**)	=	**49.39**
Prob > chi2	=	**0.0000**
Pseudo R2	=	**0.0051**

Log likelihood = **-4790.3067**

sugar200	Odds Ratio	Std. Err.	z	P>|z|	[95% Conf.	Interval]
pattern						
0 0 Psc	**1.572864**	**.4863062**	**1.46**	**0.143**	**.8580492**	**2.883169**
0 Nuc 0	**1.580826**	**.1159171**	**6.25**	**0.000**	**1.369204**	**1.825156**
0 Nuc Psc	**.8332433**	**.2614165**	**-0.58**	**0.561**	**.4505275**	**1.54107**
Cort 0 0	**1.275904**	**.5095768**	**0.61**	**0.542**	**.5832582**	**2.791098**
Cort 0 Psc	**1**	(empty)				
Cort Nuc 0	**.7172221**	**.1287934**	**-1.85**	**0.064**	**.5044304**	**1.019779**
Cort Nuc Psc	**.6139688**	**.1638292**	**-1.83**	**0.068**	**.3639274**	**1.035805**
_cons	**.0857235**	**.0027461**	**-76.69**	**0.000**	**.0805068**	**.0912784**

Note: **_cons** estimates baseline odds.

```
87.
88. * Vision in cataractous eyes
89. tab2 pva cortAny nucAny pscAny, firstonly nokey  chi  col
```

-> tabulation of pva by cortAny

pva	cortAny 0	1	Total
No VI	11,655<br>72.72	93<br>9.48	11,748<br>69.07
Mild VI	1,137<br>7.09	80<br>8.15	1,217<br>7.16
Moderate VI	2,580<br>16.10	475<br>48.42	3,055<br>17.96
Severe VI	175<br>1.09	54<br>5.50	229<br>1.35
Blind	481<br>3.00	279<br>28.44	760<br>4.47
Total	16,028<br>100.00	981<br>100.00	17,009<br>100.00

Pearson chi2(**4**) = **2.5e+03** Pr = **0.000**

-> tabulation of pva by nucAny

pva	nucAny 0	1	Total
No VI	11,324<br>82.26	424<br>13.07	11,748<br>69.07
Mild VI	912<br>6.63	305<br>9.40	1,217<br>7.16
Moderate VI	1,370<br>9.95	1,685<br>51.96	3,055<br>17.96
Severe VI	40<br>0.29	189<br>5.83	229<br>1.35
Blind	120<br>0.87	640<br>19.73	760<br>4.47
Total	13,766<br>100.00	3,243<br>100.00	17,009<br>100.00

Pearson chi2(**4**) = **7.1e+03** Pr = **0.000**

-> tabulation of pva by pscAny

pva	pscAny 0	1	Total
No VI	11,679<br>71.08	69<br>11.94	11,748<br>69.07
Mild VI	1,169<br>7.11	48<br>8.30	1,217<br>7.16
Moderate VI	2,784<br>16.94	271<br>46.89	3,055<br>17.96
Severe VI	197<br>1.20	32<br>5.54	229<br>1.35
Blind	602	158	760

	3.66	27.34	4.47
Total	16,431<br>100.00	578<br>100.00	17,009<br>100.00

Pearson chi2(**4**) = **1.3e+03** Pr = **0.000**

90. tab2 bva cortAny nucAny pscAny, firstonly nokey chi col

-> tabulation of bva by cortAny

bva	cortAny 0	1	Total
No VI	14,349<br>89.52	349<br>35.58	14,698<br>86.41
Mild VI	417<br>2.60	89<br>9.07	506<br>2.97
Moderate VI	765<br>4.77	271<br>27.62	1,036<br>6.09
Severe VI	94<br>0.59	34<br>3.47	128<br>0.75
Blind	403<br>2.51	238<br>24.26	641<br>3.77
Total	16,028<br>100.00	981<br>100.00	17,009<br>100.00

Pearson chi2(**4**) = **2.5e+03** Pr = **0.000**

-> tabulation of bva by nucAny

bva	nucAny 0	1	Total
No VI	13,258<br>96.31	1,440<br>44.40	14,698<br>86.41
Mild VI	161<br>1.17	345<br>10.64	506<br>2.97
Moderate VI	237<br>1.72	799<br>24.64	1,036<br>6.09
Severe VI	20<br>0.15	108<br>3.33	128<br>0.75
Blind	90<br>0.65	551<br>16.99	641<br>3.77
Total	13,766<br>100.00	3,243<br>100.00	17,009<br>100.00

Pearson chi2(**4**) = **6.1e+03** Pr = **0.000**

-> tabulation of bva by pscAny

bva	pscAny 0	1	Total
No VI	14,506<br>88.28	192<br>33.22	14,698<br>86.41
Mild VI	450<br>2.74	56<br>9.69	506<br>2.97
Moderate VI	862<br>5.25	174<br>30.10	1,036<br>6.09
Severe VI	113<br>0.69	15<br>2.60	128<br>0.75
Blind	500<br>3.04	141<br>24.39	641<br>3.77
Total	16,431<br>100.00	578<br>100.00	17,009<br>100.00

Pearson chi2(**4**) = **1.6e+03** Pr = **0.000**

91. * pure
92. tab2 pattern pva if pattern !=1 & pattern !=., firstonly nokey chi row

-> tabulation of pattern by pva if pattern !=1 & pattern !=.

group(cort_locs nuc_locs psc_locs)	No VI	Mild VI	pva Moderate	Severe VI	Blind	Total
0 0 Psc	29<br>29.59	11<br>11.22	34<br>34.69	4<br>4.08	20<br>20.41	98<br>100.00
0 Nuc 0	330<br>15.15	226<br>10.38	1,162<br>53.35	130<br>5.97	330<br>15.15	2,178<br>100.00
0 Nuc Psc	22<br>13.17	6<br>3.59	83<br>49.70	9<br>5.39	47<br>28.14	167<br>100.00
Cort 0 0	21<br>29.58	6<br>8.45	28<br>39.44	4<br>5.63	12<br>16.90	71<br>100.00
Cort 0 Psc	0<br>0.00	1<br>8.33	7<br>58.33	0<br>0.00	4<br>33.33	12<br>100.00
Cort Nuc 0	54<br>9.05	43<br>7.20	293<br>49.08	31<br>5.19	176<br>29.48	597<br>100.00
Cort Nuc Psc	18<br>5.98	30<br>9.97	147<br>48.84	19<br>6.31	87<br>28.90	301<br>100.00
Total	474<br>13.84	323<br>9.43	1,754<br>51.23	197<br>5.75	676<br>19.74	3,424<br>100.00

Pearson chi2(**24**) = **153.9316** Pr = **0.000**

93. tab2 pattern bva if pattern !=1 & pattern !=., firstonly nokey chi row

-> tabulation of pattern by bva if pattern !=1 & pattern !=.

group(cort_locs nuc_locs psc_locs)	No VI	Mild VI	bva Moderate	Severe VI	Blind	Total
0 0 Psc	53<br>54.08	8<br>8.16	16<br>16.33	4<br>4.08	17<br>17.35	98<br>100.00
0 Nuc 0	1,087<br>49.91	241<br>11.07	500<br>22.96	70<br>3.21	280<br>12.86	2,178<br>100.00
0 Nuc Psc	51<br>30.54	21<br>12.57	47<br>28.14	5<br>2.99	43<br>25.75	167<br>100.00
Cort 0 0	41<br>57.75	5<br>7.04	16<br>22.54	1<br>1.41	8<br>11.27	71<br>100.00
Cort 0 Psc	6<br>50.00	1<br>8.33	3<br>25.00	0<br>0.00	2<br>16.67	12<br>100.00
Cort Nuc 0	220<br>36.85	57<br>9.55	144<br>24.12	27<br>4.52	149<br>24.96	597<br>100.00
Cort Nuc Psc	82<br>27.24	26<br>8.64	108<br>35.88	6<br>1.99	79<br>26.25	301<br>100.00
Total	1,540<br>44.98	359<br>10.48	834<br>24.36	113<br>3.30	578<br>16.88	3,424<br>100.00

Pearson chi2(**24**) = **154.2991** Pr = **0.000**

```
94.
95. tab2 pattern bva if pattern !=. & pattern !=1, mis col nokey nofreq chi
```

-> tabulation of pattern by bva if pattern !=. & pattern !=1

group(cort_locs nuc_locs psc_locs)	No VI	Mild VI	bva Moderate	Severe VI	Blind	.	Total
0 0 Psc	3.44	2.23	1.92	3.54	2.94	5.88	2.91
0 Nuc 0	70.58	67.13	59.95	61.95	48.44	94.12	64.06
0 Nuc Psc	3.31	5.85	5.64	4.42	7.44	0.00	4.81
Cort 0 0	2.66	1.39	1.92	0.88	1.38	0.00	2.04
Cort 0 Psc	0.39	0.28	0.36	0.00	0.35	0.00	0.35
Cort Nuc 0	14.29	15.88	17.27	23.89	25.78	0.00	17.18
Cort Nuc Psc	5.32	7.24	12.95	5.31	13.67	0.00	8.66
Total	100.00	100.00	100.00	100.00	100.00	100.00	100.00

Pearson chi2(**30**) = **181.6514** Pr = **0.000**

```
96.
97.
98.
99. ** Prevalence of types of cataract in persons
100 use "UVR-cataract-analysis.dta", clear

101 save "combinedUVR40+_7.dta", replace
  file combinedUVR40+_7.dta saved

102 local dateAn: di c(current_date)

103
104
105 tab1 cort_prev nuc_prev psc_prev if excludeLocs==0
```

-> tabulation of cort_prev if excludeLocs==0

cort_prev	Freq.	Percent	Cum.
NormOrNotcort	8,232	93.06	93.06
cort	614	6.94	100.00
Total	8,846	100.00	

-> tabulation of nuc_prev if excludeLocs==0

nuc_prev	Freq.	Percent	Cum.
NormOrNotnuc	6,914	78.16	78.16
nuc	1,932	21.84	100.00
Total	8,846	100.00	

-> tabulation of psc_prev if excludeLocs==0

psc_prev	Freq.	Percent	Cum.
NormOrNotpsc	8,435	95.35	95.35
psc	411	4.65	100.00
Total	8,846	100.00	

```
106 tab2 study_location cort_prev nuc_prev psc_prev if excludeLocs==0 , firstonly row no
  > key chi
```

-> tabulation of study_location by cort_prev if excludeLocs==0

study_locat	cort_prev		
ion	NormOrNot	cort	Total
Delhi(1)	2,819	351	3,170
	88.93	11.07	100.00
Guwahati(2)	2,842	243	3,085
	92.12	7.88	100.00
Prakasam(3)	2,571	20	2,591
	99.23	0.77	100.00
Total	8,232	614	8,846
	93.06	6.94	100.00

Pearson chi2(**2**) = **240.6182**   Pr = **0.000**

-> tabulation of study_location by nuc_prev if excludeLocs==0

study_location	nuc_prev NormOrNot	nuc	Total
Delhi(1)	2,572<br>81.14	598<br>18.86	3,170<br>100.00
Guwahati(2)	2,465<br>79.90	620<br>20.10	3,085<br>100.00
Prakasam(3)	1,877<br>72.44	714<br>27.56	2,591<br>100.00
Total	6,914<br>78.16	1,932<br>21.84	8,846<br>100.00

Pearson chi2(**2**) = **71.5397** Pr = **0.000**

-> tabulation of study_location by psc_prev if excludeLocs==0

study_location	psc_prev NormOrNot	psc	Total
Delhi(1)	2,846<br>89.78	324<br>10.22	3,170<br>100.00
Guwahati(2)	3,031<br>98.25	54<br>1.75	3,085<br>100.00
Prakasam(3)	2,558<br>98.73	33<br>1.27	2,591<br>100.00
Total	8,435<br>95.35	411<br>4.65	8,846<br>100.00

Pearson chi2(**2**) = **347.2729** Pr = **0.000**

```
*** Distribution of pure cataracts
tab1 cort_assoc nuc_assoc psc_assoc pure_assoc
```

-> tabulation of cort_assoc

cort_assoc	Freq.	Percent	Cum.
0	6,640	99.43	99.43
Cort	38	0.57	100.00
Total	6,678	100.00	

-> tabulation of nuc_assoc

nuc_assoc	Freq.	Percent	Cum.
0	6,640	84.63	84.63
Nuc	1,206	15.37	100.00
Total	7,846	100.00	

-> tabulation of psc_assoc

psc_assoc	Freq.	Percent	Cum.
0	6,640	99.00	99.00
Psc	67	1.00	100.00
Total	6,707	100.00	

-> tabulation of pure_assoc

pure_assoc	Freq.	Percent	Cum.
0	6,640	83.51	83.51
1	38	0.48	83.99
2	1,206	15.17	99.16
3	67	0.84	100.00
Total	7,951	100.00	

```
110 tab2 study_location  cort_assoc nuc_assoc psc_assoc pure_assoc , firstonly row nokey
  >  chi
```

-> tabulation of study_location by cort_assoc

study_location	cort_assoc 0	Cort	Total
Delhi(1)	2,465<br>99.52	12<br>0.48	2,477<br>100.00
Guwahati(2)	2,388<br>99.58	10<br>0.42	2,398<br>100.00
Prakasam(3)	1,787<br>99.11	16<br>0.89	1,803<br>100.00
Total	6,640<br>99.43	38<br>0.57	6,678<br>100.00

Pearson chi2(**2**) = **4.5227** Pr = **0.104**

-> tabulation of study_location by nuc_assoc

study_location	nuc_assoc 0	Nuc	Total
Delhi(1)	2,465<br>93.69	166<br>6.31	2,631<br>100.00
Guwahati(2)	2,388<br>87.25	349<br>12.75	2,737<br>100.00
Prakasam(3)	1,787<br>72.11	691<br>27.89	2,478<br>100.00
Total	6,640<br>84.63	1,206<br>15.37	7,846<br>100.00

Pearson chi2(**2**) = **478.8528** Pr = **0.000**

-> tabulation of study_location by psc_assoc

study_location	psc_assoc 0	Psc	Total
Delhi(1)	2,465<br>99.12	22<br>0.88	2,487<br>100.00
Guwahati(2)	2,388<br>99.25	18<br>0.75	2,406<br>100.00
Prakasam(3)	1,787<br>98.51	27<br>1.49	1,814<br>100.00
Total	6,640<br>99.00	67<br>1.00	6,707<br>100.00

Pearson chi2(**2**) = **6.2538** Pr = **0.044**

-> tabulation of study_location by pure_assoc

study_location	pure_assoc 0	1	2	3	Total
Delhi(1)	2,465<br>92.50	12<br>0.45	166<br>6.23	22<br>0.83	2,665<br>100.00
Guwahati(2)	2,388<br>86.37	10<br>0.36	349<br>12.62	18<br>0.65	2,765<br>100.00
Prakasam(3)	1,787<br>70.88	16<br>0.63	691<br>27.41	27<br>1.07	2,521<br>100.00
Total	6,640<br>83.51	38<br>0.48	1,206<br>15.17	67<br>0.84	7,951<br>100.00

Pearson chi2(**6**) = **482.7692**   Pr = **0.000**

```
*** Logistic Regtression Analysis
logistic anyCataract ///
>         gender i.educat2 smoke20 indoor16_kitchen i.tExposureQuantilesAll ///
>                 if educat2<5 & study_location  ==1, nolog
```

Logistic regression

Number of obs	=	**3,587**
LR chi2(**11**)	=	**702.13**
Prob > chi2	=	**0.0000**
Pseudo R2	=	**0.1563**

Log likelihood = **-1894.613**

anyCataract	Odds Ratio	Std. Err.	z	P>|z|	[95% Conf. Interval
>]						
gender	**.5627393**	**.0895485**	**-3.61**	**0.000**	**.4119608**	**.768703**
**> 2**						
educat2						
Can read & write	**.5565066**	**.06911**	**-4.72**	**0.000**	**.4362782**	**.709867**
**> 2**						
Middle School (6-8)	**.3540028**	**.0541499**	**-6.79**	**0.000**	**.2623029**	**.477760**
**> 7**						
High School (9-12)	**.4232057**	**.0577947**	**-6.30**	**0.000**	**.3238232**	**.553089**
**> 1**						
Graduation	**.6263333**	**.1779921**	**-1.65**	**0.100**	**.3588476**	**1.09320**
**> 3**						
smoke20	**1.364824**	**.1284786**	**3.30**	**0.001**	**1.134875**	**1.64136**
**> 4**						
indoor16_kitchen	**1.340887**	**.1934115**	**2.03**	**0.042**	**1.01068**	**1.77897**
**> 8**						
tExposureQuantilesAll						
2	**1.217642**	**.2326445**	**1.03**	**0.303**	**.8373132**	**1.77072**
**> 7**						
3	**1.33442**	**.2401285**	**1.60**	**0.109**	**.9378194**	**1.89874**
**> 3**						
4	**2.166069**	**.3653978**	**4.58**	**0.000**	**1.556259**	**3.01483**
**> 1**						
5	**7.53346**	**1.22883**	**12.38**	**0.000**	**5.472045**	**10.3714**
**> 5**						
_cons	**.4147931**	**.1198386**	**-3.05**	**0.002**	**.2354558**	**.730724**
**> 4**						

Note: **_cons** estimates baseline odds.

```
114 est sto loc1

115 logistic anyCataract ///
  >         gender i.educat2 smoke20 indoor16_kitchen i.tExposureQuantilesAll ///
  >                 if educat2<5 & study_location  ==2, nolog

  Logistic regression                               Number of obs     =      3,207
                                                    LR chi2(11)       =     569.64
                                                    Prob > chi2       =     0.0000
  Log likelihood = -1569.6868                       Pseudo R2         =     0.1536

  ──────────────────────┬────────────────────────────────────────────────────────────
    _
          anyCataract │ Odds Ratio   Std. Err.      z    P>|z|     [95% Conf. Interval
  > ]
  ──────────────────────┼────────────────────────────────────────────────────────────
    _
               gender │   1.164095   .1398044     1.27   0.206     .9199442    1.47304
  > 2
                      │
              educat2 │
      Can read & write │    .659855   .0749383    -3.66   0.000     .5281768    .824361
  > 3
   Middle School (6-8) │   .6221617    .104369    -2.83   0.005     .4478291    .864359
  > 1
    High School (9-12) │   .5021808   .0683607    -5.06   0.000     .3845812    .655740
  > 7
            Graduation │   .3609456   .1355736    -2.71   0.007      .172872    .753631
  > 3
                      │
              smoke20 │   1.465015   .1856683     3.01   0.003     1.142787    1.87810
  > 1
     indoor16_kitchen │   1.289768   .2266879     1.45   0.148     .9139146    1.82019
  > 4
                      │
  tExposureQuantilesAll │
                    2 │   2.397287   .3412168     6.14   0.000     1.813697    3.16865
  > 8
                    3 │   6.508472     .95427    12.78   0.000     4.882881    8.67524
  > 8
                    4 │   10.57358   1.752456    14.23   0.000     7.640907    14.6318
  > 6
                    5 │   25.67425   6.896602    12.08   0.000     15.16522    43.4657
  > 4
                      │
                _cons │   .0794545   .0232141    -8.67   0.000     .0448151    .140868
  > 1
  ──────────────────────┴────────────────────────────────────────────────────────────
    _
  Note: _cons estimates baseline odds.

116 est sto loc2

117 logistic anyCataract ///
  >         gender i.educat2 smoke20 indoor16_kitchen i.tExposureQuantilesAll ///
  >                 if educat2<5 & study_location  ==3, nolog

  Logistic regression                               Number of obs     =      2,902
                                                    LR chi2(11)       =     597.45
                                                    Prob > chi2       =     0.0000
  Log likelihood = -1678.9942                       Pseudo R2         =     0.1510
```

```
────────────────────────────────────────────────────────────────────────────────────
  _
         anyCataract │ Odds Ratio   Std. Err.      z    P>|z|     [95% Conf. Interval
> ]
─────────────────────┼──────────────────────────────────────────────────────────────
  _
              gender │   .9749971   .2265448    -0.11   0.913     .6183338    1.53738
> 9
                     │
             educat2 │
    Can read & write │   .7027222   .0829431    -2.99   0.003     .5575906    .885629
> 2
 Middle School (6-8) │   .6795925   .1265055    -2.08   0.038     .4718438    .978811
> 1
  High School (9-12) │   .4024986   .0700716    -5.23   0.000     .2861392     .56617
> 6
          Graduation │   .1131929   .0605845    -4.07   0.000     .0396487     .32315
> 4
                     │
             smoke20 │   1.427572   .1729432     2.94   0.003     1.125847     1.8101
> 6
     indoor16_kitchen │    1.35786   .2929006     1.42   0.156     .8897019    2.07236
> 2
                     │
tExposureQuantilesAll │
                   2 │   .5385644   .0960985    -3.47   0.001     .3796241    .764049
> 4
                   3 │   .4563221   .0629621    -5.69   0.000      .348197    .598023
> 1
                   4 │   1.054592   .1312067     0.43   0.669     .8263854    1.34581
> 9
                   5 │   5.013201   .6817703    11.85   0.000     3.840219    6.54446
> 7
                     │
               _cons │   .5656296   .1777852    -1.81   0.070     .3054835    1.04731
> 3
────────────────────────────────────────────────────────────────────────────────────
  _
Note: _cons estimates baseline odds.
```

118 est sto loc3

119 logistic anyCataract ///

```
>         gender i.educat2 smoke20 indoor16_kitchen i.tExposureQuantilesAll i.study_lo
> cation ///
>                 if educat2<5 , nolog

Logistic regression                               Number of obs     =      9,696
                                                  LR chi2(13)       =    1758.05
                                                  Prob > chi2       =     0.0000
Log likelihood = -5287.0569                       Pseudo R2         =     0.1426

────────────────────────────────────────────────────────────────────────────────────
  _
         anyCataract │ Odds Ratio   Std. Err.      z    P>|z|     [95% Conf. Interval
> ]
─────────────────────┼──────────────────────────────────────────────────────────────
  _
              gender │   .9269099   .0750717    -0.94   0.349     .7908562    1.08636
> 9
                     │
             educat2 │
    Can read & write │   .6604907    .044212    -6.20   0.000     .5792806    .753085
> 9
 Middle School (6-8) │   .5370256   .0503385    -6.63   0.000     .4468966    .645331
> 7
  High School (9-12) │   .4688843   .0379436    -9.36   0.000      .400114    .549474
> 7
          Graduation │   .3798319   .0756661    -4.86   0.000     .2570538    .561253
> 3
                     │
             smoke20 │   1.374743   .0860931     5.08   0.000     1.215948    1.55427
```

```
> 5
     indoor16_kitchen |   1.214542   .0994495     2.37   0.018     1.034461    1.42597
> 2
                      |
tExposureQuantilesAll |
                    2 |   1.077821   .0933004     0.87   0.387     .9096271    1.27711
> 5
                    3 |   1.647977   .1360909     6.05   0.000     1.401711    1.93750
> 9
                    4 |   2.628959   .2179998    11.66   0.000     2.234601    3.09291
> 2
                    5 |    9.42244   .8331248    25.37   0.000     7.923204    11.2053
> 6
                      |
       study_location |
         Guwahati(2)  |   1.956389   .1415596     9.27   0.000     1.697713    2.25447
> 8
         Prakasam(3)  |   1.988732    .126966    10.77   0.000     1.754823     2.2538
> 2
                      |
                _cons |   .1582955   .0260081   -11.22   0.000     .1147137    .218434
> 9
-------------------------------------------------------------------------------------
  _
Note: _cons estimates baseline odds.

120 est sto locAll

121
122 /*
  > // Logistic Regression Analysis *** With Pack Year Smokeing
  > logistic anyCataract ///
  >         gender i.educat2 smoke20 i.smokepackTotCat3 indoor16_kitchen i.tExposureQuan
  > tilesAll ///
  >                 if educat2<5 & study_location  ==1 & smokepackTotCat3!=99 , nolog
  > logistic anyCataract ///
  >         gender i.educat2 smoke20 indoor16_kitchen i.tExposureQuantilesAll ///
  >                 if educat2<5 & study_location  ==2, nolog
  > logistic anyCataract ///
  >         gender i.educat2 smoke20 indoor16_kitchen i.tExposureQuantilesAll ///
  >                 if educat2<5 & study_location  ==3, nolog
  > logistic anyCataract ///
  >         gender i.educat2 smoke20 indoor16_kitchen i.tExposureQuantilesAll i.study_lo
  > cation ///
  >                 if educat2<5 , nolog
  > */
123
124
125 logistic anyCataract ///
  >         gender i.educat2 smoke20 indoor16_kitchen i.tExposureQuantilesAll  i.sugar20
  > 0  ///
  >                 if educat2<5 & study_location  ==1, nolog

Logistic regression                             Number of obs     =      3,564
                                                LR chi2(12)       =     711.25
                                                Prob > chi2       =     0.0000
Log likelihood = -1877.4219                     Pseudo R2         =     0.1593

-------------------------------------------------------------------------------------
  _
          anyCataract | Odds Ratio   Std. Err.      z    P>|z|     [95% Conf. Interval
> ]
-------------------------------------------------------------------------------------
  _
               gender |   .5628582   .0899192    -3.60   0.000     .4115434    .769807
> 9
                      |
              educat2 |
    Can read & write  |   .5534589   .0691024    -4.74   0.000     .4333195    .706907
> 5
 Middle School (6-8)  |   .3509581   .0539024    -6.82   0.000     .2597301    .474229
> 3
```

```
  High School (9-12)  |   .4134022   .0568477    -6.42   0.000     .3157348    .541281
> 5
         Graduation   |   .6050027   .1729532    -1.76   0.079     .3454799    1.05947
> 8
                      |
            smoke20   |   1.372989   .1299015     3.35   0.001     1.140599    1.65272
> 7
    indoor16_kitchen  |     1.3206   .1910381     1.92   0.055     .9945719    1.75350
> 1
                      |
tExposureQuantilesAll |
                  2   |   1.242329   .2378249     1.13   0.257     .8536638    1.80794
> 9
                  3   |    1.37008    .247214     1.75   0.081       .96196    1.95134
> 8
                  4   |   2.213943   .3745098     4.70   0.000     1.589196    3.08429
> 1
                  5   |   7.742998   1.267761    12.50   0.000     5.617485    10.6727
> 5
                      |
         1.sugar200   |   1.740088   .2907381     3.32   0.001     1.254152    2.41430
> 4
              _cons   |   .3998426   .1160148    -3.16   0.002     .2264185    .706099
> 9
----------------------------------------------------------------------------------------
  _
Note: _cons estimates baseline odds.

126 est sto loc11

127 logistic anyCataract ///
>        gender i.educat2 smoke20 indoor16_kitchen i.tExposureQuantilesAll i.sugar200
>    ///
>                if educat2<5 & study_location  ==2, nolog

Logistic regression                             Number of obs     =      3,141
                                                LR chi2(12)       =     557.20
                                                Prob > chi2       =     0.0000
Log likelihood = -1539.2591                     Pseudo R2         =     0.1533

----------------------------------------------------------------------------------------
  _
         anyCataract  | Odds Ratio   Std. Err.      z    P>|z|     [95% Conf. Interval
> ]
----------------------------------------------------------------------------------------
  _
             gender   |   1.148568    .139107     1.14   0.253     .9058673    1.45629
> 2
                      |
            educat2   |
    Can read & write  |   .6383611   .0733669    -3.91   0.000     .5096098     .79964
> 1
 Middle School (6-8)  |   .6280626   .1059798    -2.76   0.006     .4512014    .874249
> 7
  High School (9-12)  |   .5065642   .0700205    -4.92   0.000     .3863456    .664190
> 9
         Graduation   |   .3601285   .1360016    -2.70   0.007     .1717922    .754938
> 4
                      |
            smoke20   |   1.437384   .1837313     2.84   0.005     1.118843    1.84661
> 4
    indoor16_kitchen  |    1.27122   .2238697     1.36   0.173     .9001578     1.7952
> 4
                      |
tExposureQuantilesAll |
                  2   |   2.367873   .3417836     5.97   0.000     1.784408    3.14211
> 8
                  3   |   6.575926   .9776631    12.67   0.000     4.913672    8.80050
> 7
                  4   |   10.32156    1.73047    13.92   0.000     7.430815    14.3368
> 7
                  5   |   25.28855   6.822495    11.97   0.000     14.90327    42.9107
```

```
> 5
                          |
             1.sugar200   |   1.145926   .2230311     0.70   0.484     .7825057     1.6781
> 3
                  _cons   |   .0828693   .0243469    -8.48   0.000     .0465921    .147392
> 6
--------------------------------------------------------------------------------------------
  _
Note: _cons estimates baseline odds.
```

128 est sto loc12

129 logistic anyCataract ///

```
>         gender i.educat2 smoke20 indoor16_kitchen i.tExposureQuantilesAll i.sugar200
>    ///
>                 if educat2<5 & study_location  ==3, nolog

Logistic regression                             Number of obs     =      2,836
                                                LR chi2(12)       =     603.35
                                                Prob > chi2       =     0.0000
Log likelihood = -1631.3675                     Pseudo R2         =     0.1561

--------------------------------------------------------------------------------------------
  _
            anyCataract | Odds Ratio   Std. Err.      z    P>|z|     [95% Conf. Interval
> ]
--------------------------------------------------------------------------------------------
  _
                 gender |    .939887   .2213641    -0.26   0.792      .592378    1.49125
> 6
                        |
                educat2 |
      Can read & write  |   .6567706   .0790308    -3.49   0.000      .518784    .831459
> 1
   Middle School (6-8)  |     .63851    .121241    -2.36   0.018     .4400892     .92639
> 2
    High School (9-12)  |    .375214   .0664225    -5.54   0.000     .2652129    .530839
> 7
            Graduation  |   .1073612   .0574348    -4.17   0.000     .0376255    .306346
> 5
                        |
                smoke20 |   1.427559   .1760298     2.89   0.004     1.121073    1.81783
> 4
       indoor16_kitchen |    1.41234    .308437     1.58   0.114      .920551     2.1668
> 6
                        |
tExposureQuantilesAll   |
                      2 |   .5384105   .0984742    -3.39   0.001     .3762101    .770542
> 4
                      3 |   .4524056   .0640291    -5.60   0.000     .3428129    .597033
> 5
                      4 |   1.079291   .1370861     0.60   0.548     .8414405    1.38437
> 6
                      5 |   5.070824   .7022539    11.72   0.000     3.865418    6.65212
> 9
                        |
             1.sugar200 |   1.416708   .1651396     2.99   0.003     1.127352    1.78033
> 3
                  _cons |   .5594679   .1794691    -1.81   0.070     .2983474    1.04912
> 7
--------------------------------------------------------------------------------------------
  _
Note: _cons estimates baseline odds.
```

```
130 est sto loc13

131 logistic anyCataract ///
  >         gender i.educat2 smoke20 indoor16_kitchen i.tExposureQuantilesAll i.study_lo
  > cation  i.sugar200  ///
  >                 if educat2<5 , nolog

  Logistic regression                               Number of obs     =      9,541
                                                    LR chi2(14)       =    1763.82
                                                    Prob > chi2       =     0.0000
  Log likelihood = -5188.0629                       Pseudo R2         =     0.1453

  ----------------------+----------------------------------------------------------------
  _
          anyCataract | Odds Ratio   Std. Err.      z    P>|z|     [95% Conf. Interval
  > ]
  ----------------------+----------------------------------------------------------------
  _
               gender |   .9122798   .0746721    -1.12   0.262     .7770614    1.07102
  > 8
                      |
              educat2 |
    Can read & write  |   .6330921   .0429239    -6.74   0.000      .554313    .723067
  > 2
   Middle School (6-8) |   .5238093   .0495461    -6.84   0.000     .4351707    .630502
  > 3
    High School (9-12) |   .4542244   .0371995    -9.64   0.000     .3868653    .533311
  > 7
           Graduation |    .362398   .0725635    -5.07   0.000     .2447645     .53656
  > 6
                      |
              smoke20 |   1.366968   .0863799     4.95   0.000     1.207731    1.54720
  > 1
     indoor16_kitchen |   1.221469   .1009442     2.42   0.015     1.038814     1.4362
  > 4
                      |
  tExposureQuantilesAll |
                    2 |   1.072411   .0940005     0.80   0.425     .9031306    1.27342
  > 1
                    3 |   1.657421   .1382779     6.06   0.000       1.4074    1.95185
  > 8
                    4 |   2.646684   .2215953    11.62   0.000     2.246129     3.1186
  > 7
                    5 |    9.47907   .8450977    25.23   0.000     7.959351    11.2889
  > 6
                      |
       study_location |
          Guwahati(2) |   1.954937   .1426255     9.19   0.000     1.694462    2.25545
  > 1
          Prakasam(3) |   1.878646   .1219913     9.71   0.000     1.654137    2.13362
  > 7
                      |
           1.sugar200 |   1.579041   .1336159     5.40   0.000     1.337723    1.86389
  > 2
                _cons |   .1606328   .0266507   -11.02   0.000     .1160408    .222360
  > 6
  ----------------------+----------------------------------------------------------------
  _
  Note: _cons estimates baseline odds.
```

```
132 est sto locAll_1

133
134 estout loc1 loc2 loc3 locAll, eform cells(b(star fmt(2)) p(fmt(3))) lab
```

	loc1<br>b/p	loc2<br>b/p	loc3<br>b/p	locAll<br>b/p
**anyCataract**				
gender	0.56***<br>0.000	1.16<br>0.206	0.97<br>0.913	0.93<br>0.349
Illiterate	1.00<br>.	1.00<br>.	1.00<br>.	1.00<br>.
Can read & write	0.56***<br>0.000	0.66***<br>0.000	0.70**<br>0.003	0.66***<br>0.000
Middle School (6-8)	0.35***<br>0.000	0.62**<br>0.005	0.68*<br>0.038	0.54***<br>0.000
High School (9-12)	0.42***<br>0.000	0.50***<br>0.000	0.40***<br>0.000	0.47***<br>0.000
Graduation	0.63<br>0.100	0.36**<br>0.007	0.11***<br>0.000	0.38***<br>0.000
smoke20	1.36***<br>0.001	1.47**<br>0.003	1.43**<br>0.003	1.37***<br>0.000
indoor16_kitchen	1.34*<br>0.042	1.29<br>0.148	1.36<br>0.156	1.21*<br>0.018
5 quantiles of tEx~1	1.00<br>.	1.00<br>.	1.00<br>.	1.00<br>.
5 quantiles of tEx~2	1.22<br>0.303	2.40***<br>0.000	0.54***<br>0.001	1.08<br>0.387
5 quantiles of tEx~3	1.33<br>0.109	6.51***<br>0.000	0.46***<br>0.000	1.65***<br>0.000
5 quantiles of tEx~4	2.17***<br>0.000	10.57***<br>0.000	1.05<br>0.669	2.63***<br>0.000
5 quantiles of tEx~5	7.53***<br>0.000	25.67***<br>0.000	5.01***<br>0.000	9.42***<br>0.000
Delhi(1)				1.00<br>.
Guwahati(2)				1.96***<br>0.000
Prakasam(3)				1.99***<br>0.000
_cons	0.41**<br>0.002	0.08***<br>0.000	0.57<br>0.070	0.16***<br>0.000

```
135 di "With Diabetes"
```
With Diabetes

```
136 estout loc11 loc12 loc13 locAll_1, eform cells(b( star  fmt(2)) p(fmt(3))) lab
```

	loc11<br>b/p	loc12<br>b/p	loc13<br>b/p	locAll_1<br>b/p
**anyCataract**				
gender	0.56***<br>0.000	1.15<br>0.253	0.94<br>0.792	0.91<br>0.262
Illiterate	1.00<br>.	1.00<br>.	1.00<br>.	1.00<br>.
Can read & write	0.55***<br>0.000	0.64***<br>0.000	0.66***<br>0.000	0.63***<br>0.000
Middle School (6-8)	0.35***<br>0.000	0.63**<br>0.006	0.64*<br>0.018	0.52***<br>0.000
High School (9-12)	0.41***<br>0.000	0.51***<br>0.000	0.38***<br>0.000	0.45***<br>0.000
Graduation	0.61<br>0.079	0.36**<br>0.007	0.11***<br>0.000	0.36***<br>0.000
smoke20	1.37***<br>0.001	1.44**<br>0.005	1.43**<br>0.004	1.37***<br>0.000
indoor16_kitchen	1.32<br>0.055	1.27<br>0.173	1.41<br>0.114	1.22*<br>0.015

5 quantiles of tEx~1	1.00	1.00	1.00	1.00
	.	.	.	.
5 quantiles of tEx~2	1.24	2.37***	0.54***	1.07
	0.257	0.000	0.001	0.425
5 quantiles of tEx~3	1.37	6.58***	0.45***	1.66***
	0.081	0.000	0.000	0.000
5 quantiles of tEx~4	2.21***	10.32***	1.08	2.65***
	0.000	0.000	0.548	0.000
5 quantiles of tEx~5	7.74***	25.29***	5.07***	9.48***
	0.000	0.000	0.000	0.000
sugar200=0	1.00	1.00	1.00	1.00
	.	.	.	.
sugar200=1	1.74***	1.15	1.42**	1.58***
	0.001	0.484	0.003	0.000
Delhi(1)				1.00
				.
Guwahati(2)				1.95***
				0.000
Prakasam(3)				1.88***
				0.000
_cons	0.40**	0.08***	0.56	0.16***
	0.002	0.000	0.070	0.000

```
*** Muninomial Lostic TRegression of pure cataracts
mlogit pure_assoc gender i.educat2 smoke20 indoor16_kitchen i.tExposureQuantilesAll
  > i.study_location if educat2<5, allbaselevels  nolog
```

Multinomial logistic regression Number of obs = 7,935
LR chi2(39) = 1138.63
Prob > chi2 = 0.0000
Log likelihood = -3416.6865 Pseudo R2 = 0.1428

_ pure_assoc >]	Coef.	Std. Err.	z	P>|z|	[95% Conf. Interval
_ 0	(base outcome)					
_ 1						
gender	-.5063656	.5640616	-0.90	0.369	-1.611906	.599174
> 8						
educat2						
Illiterate	0	(base)				
Can read & write	-.5382653	.4774667	-1.13	0.260	-1.474083	.397552
> 2						
Middle School (6-8)	-1.681295	1.040678	-1.62	0.106	-3.720987	.358397
> 7						
High School (9-12)	-.6431838	.5495617	-1.17	0.242	-1.720305	.433937
> 5						
Graduation	-13.46945	725.5199	-0.02	0.985	-1435.462	1408.52
> 3						
smoke20	.0945115	.4102408	0.23	0.818	-.7095457	.898568
> 7						
indoor16_kitchen	.1207513	.5574276	0.22	0.829	-.9717867	1.21328
> 9						
tExposureQuantilesAll						
1	0	(base)				
2	1.336689	.7980449	1.67	0.094	-.2274502	2.90082
> 9						
3	1.033413	.8284534	1.25	0.212	-.5903255	2.65715

```
> 2
                   4 |   1.258725   .8302683     1.52   0.130    -.3685714    2.88602
> 1
                   5 |   2.618798   .8031306     3.26   0.001     1.044691    4.19290
> 5
                     |
      study_location |
            Delhi(1) |          0  (base)
         Guwahati(2) |   .4261454   .5407398     0.79   0.431    -.6336852    1.48597
> 6
         Prakasam(3) |   .6230743   .4284841     1.45   0.146     -.216739    1.46288
> 8
                     |
               _cons |  -5.896327   1.240614    -4.75   0.000    -8.327886   -3.46476
> 9
---------------------+----------------------------------------------------------------
  _
2                    |
              gender |  -.0837351   .1187445    -0.71   0.481      -.31647    .148999
> 8
                     |
             educat2 |
          Illiterate |          0  (base)
    Can read & write |  -.3118968   .0944173    -3.30   0.001    -.4969512   -.126842
> 4
 Middle School (6-8) |  -.5403044   .1441311    -3.75   0.000    -.8227961   -.257812
> 6
  High School (9-12) |  -.8808761   .1293984    -6.81   0.000    -1.134492     -.6272
> 6
          Graduation |  -1.318063   .3544307    -3.72   0.000    -2.012734   -.623391
> 4
                     |
             smoke20 |   .3949066   .0920235     4.29   0.000     .2145439    .575269
> 3
     indoor16_kitchen |   .3026915   .1203168     2.52   0.012     .0668749    .538508
> 1
                     |
tExposureQuantilesAll |
                   1 |          0  (base)
                   2 |   .0651374   .1275723     0.51   0.610    -.1848998    .315174
> 5
                   3 |   .4541619   .1202815     3.78   0.000     .2184146    .689909
> 2
                   4 |   .9013931   .1207905     7.46   0.000     .6646482    1.13813
> 8
                   5 |   2.052606   .1268396    16.18   0.000     1.804005    2.30120
> 7
                     |
      study_location |
            Delhi(1) |          0  (base)
         Guwahati(2) |   1.632108   .1232003    13.25   0.000      1.39064    1.87357
> 6
         Prakasam(3) |   1.920702   .1056734    18.18   0.000     1.713586    2.12781
> 8
                     |
               _cons |  -3.775041   .2505655   -15.07   0.000     -4.26614   -3.28394
> 1
---------------------+----------------------------------------------------------------
  _
3                    |
              gender |   .0655597   .4274478     0.15   0.878    -.7722226     .90334
> 2
                     |
             educat2 |
          Illiterate |          0  (base)
    Can read & write |  -.4279883   .4090676    -1.05   0.295    -1.229746    .373769
> 5
 Middle School (6-8) |   .3701851   .4065325     0.91   0.363    -.4266039    1.16697
> 4
  High School (9-12) |   .2879153   .3671808     0.78   0.433    -.4317457    1.00757
> 6
          Graduation |  -.5394073   1.051084    -0.51   0.608    -2.599493    1.52067
```

```
> 9
                          |
                 smoke20  |   .3642696   .3297639     1.10   0.269    -.2820557    1.01059
> 5
        indoor16_kitchen  |   .3960882   .4213396     0.94   0.347    -.4297222    1.22189
> 9
                          |
tExposureQuantilesAll     |
                       1  |          0  (base)
                       2  |   .2483423   .3949401     0.63   0.529     -.525726    1.02241
> 1
                       3  |   .0638297   .4152874     0.15   0.878    -.7501187     .87777
> 8
                       4  |   .4946464   .3998811     1.24   0.216    -.2891061    1.27839
> 9
                       5  |   .6864737   .4560857     1.51   0.132    -.2074378    1.58038
> 5
                          |
          study_location  |
                Delhi(1)  |          0  (base)
             Guwahati(2)  |   .0670229   .3795339     0.18   0.860    -.6768499    .810895
> 7
            Prakasam(3)   |   .7781364   .3168569     2.46   0.014     .1571083    1.39916
> 4
                          |
                   _cons  |  -5.693908   .8573752    -6.64   0.000    -7.374332   -4.01348
> 3
--------------------------------------------------------------------------------------------
  _

//
// Gender ratio in all type of cataracts
for var anyCataract cort_prev nuc_prev psc_prev cort_assoc nuc_assoc psc_assoc pure_
> assoc: tab X gender, r col chi nokey
```

-> tab anyCataract gender, r col chi nokey

anyCataract	gender Male	Female	Total
0	2,990	3,495	6,485
	46.11	53.89	100.00
	67.71	65.94	66.75
1	1,426	1,805	3,231
	44.13	55.87	100.00
	32.29	34.06	33.25
Total	4,416	5,300	9,716
	45.45	54.55	100.00
	100.00	100.00	100.00

Pearson chi2(1) =   3.3807   Pr = 0.066

-> tab cort_prev gender, r col chi nokey

cort_prev	gender Male	Female	Total
NormOrNotcort	3,803	4,429	8,232
	46.20	53.80	100.00
	93.26	92.89	93.06
cort	275	339	614
	44.79	55.21	100.00
	6.74	7.11	6.94
Total	4,078	4,768	8,846
	46.10	53.90	100.00
	100.00	100.00	100.00

Pearson chi2(1) =   0.4568   Pr = 0.499

-> tab nuc_prev gender, r col chi nokey

nuc_prev	gender Male	Female	Total
NormOrNotnuc	3,194<br>46.20<br>78.32	3,720<br>53.80<br>78.02	6,914<br>100.00<br>78.16
nuc	884<br>45.76<br>21.68	1,048<br>54.24<br>21.98	1,932<br>100.00<br>21.84
Total	4,078<br>46.10<br>100.00	4,768<br>53.90<br>100.00	8,846<br>100.00<br>100.00

Pearson chi2(1) =   0.1179   Pr = 0.731

-> tab psc_prev gender, r col chi nokey

psc_prev	gender Male	Female	Total
NormOrNotpsc	3,890<br>46.12<br>95.39	4,545<br>53.88<br>95.32	8,435<br>100.00<br>95.35
psc	188<br>45.74<br>4.61	223<br>54.26<br>4.68	411<br>100.00<br>4.65
Total	4,078<br>46.10<br>100.00	4,768<br>53.90<br>100.00	8,846<br>100.00<br>100.00

Pearson chi2(1) =   0.0222   Pr = 0.882

-> tab cort_assoc gender, r col chi nokey

cort_assoc	gender Male	Female	Total
0	3,072<br>46.27<br>99.35	3,568<br>53.73<br>99.50	6,640<br>100.00<br>99.43
Cort	20<br>52.63<br>0.65	18<br>47.37<br>0.50	38<br>100.00<br>0.57
Total	3,092<br>46.30<br>100.00	3,586<br>53.70<br>100.00	6,678<br>100.00<br>100.00

Pearson chi2(1) =   0.6160   Pr = 0.433

-> tab nuc_assoc gender, r col chi nokey

nuc_assoc	gender Male	Female	Total
0	3,072<br>46.27<br>84.49	3,568<br>53.73<br>84.75	6,640<br>100.00<br>84.63
Nuc	564<br>46.77<br>15.51	642<br>53.23<br>15.25	1,206<br>100.00<br>15.37
Total	3,636<br>46.34<br>100.00	4,210<br>53.66<br>100.00	7,846<br>100.00<br>100.00

Pearson chi2(**1**) = **0.1031** Pr = **0.748**

-> tab psc_assoc gender, r col chi nokey

psc_assoc	gender Male	Female	Total
0	3,072<br>46.27<br>99.03	3,568<br>53.73<br>98.97	6,640<br>100.00<br>99.00
Psc	30<br>44.78<br>0.97	37<br>55.22<br>1.03	67<br>100.00<br>1.00
Total	3,102<br>46.25<br>100.00	3,605<br>53.75<br>100.00	6,707<br>100.00<br>100.00

Pearson chi2(**1**) = **0.0592** Pr = **0.808**

-> tab pure_assoc gender, r col chi nokey

pure_assoc	gender Male	Female	Total
0	3,072<br>46.27<br>83.34	3,568<br>53.73<br>83.66	6,640<br>100.00<br>83.51
1	20<br>52.63<br>0.54	18<br>47.37<br>0.42	38<br>100.00<br>0.48
2	564<br>46.77<br>15.30	642<br>53.23<br>15.05	1,206<br>100.00<br>15.17
3	30<br>44.78<br>0.81	37<br>55.22<br>0.87	67<br>100.00<br>0.84
Total	3,686<br>46.36<br>100.00	4,265<br>53.64<br>100.00	7,951<br>100.00<br>100.00

Pearson chi2(**3**) = **0.7727** Pr = **0.856**

```
148 for var anyCataract cort_prev nuc_prev psc_prev cort_assoc nuc_assoc psc_assoc pure_
  > assoc: bysort study_location: tab X gender, r col chi nokey
```

-> bysort study_location: tab anyCataract gender, r col chi nokey

-> study_location = Delhi(1)

anyCataract	gender Male	Female	Total
0	1,107<br>45.31<br>68.72	1,336<br>54.69<br>67.61	2,443<br>100.00<br>68.11
1	504<br>44.06<br>31.28	640<br>55.94<br>32.39	1,144<br>100.00<br>31.89
Total	1,611<br>44.91<br>100.00	1,976<br>55.09<br>100.00	3,587<br>100.00<br>100.00

Pearson chi2(**1**) = **0.4977** Pr = **0.480**

-> study_location = Guwahati(2)

anyCataract	gender Male	Female	Total
0	1,110<br>46.88<br>74.60	1,258<br>53.13<br>72.47	2,368<br>100.00<br>73.45
1	378<br>44.16<br>25.40	478<br>55.84<br>27.53	856<br>100.00<br>26.55
Total	1,488<br>46.15<br>100.00	1,736<br>53.85<br>100.00	3,224<br>100.00<br>100.00

Pearson chi2(**1**) = **1.8664** Pr = **0.172**

-> study_location = Prakasam(3)

anyCataract	gender Male	Female	Total
0	773<br>46.18<br>58.69	901<br>53.82<br>56.74	1,674<br>100.00<br>57.62
1	544<br>44.19<br>41.31	687<br>55.81<br>43.26	1,231<br>100.00<br>42.38
Total	1,317<br>45.34<br>100.00	1,588<br>54.66<br>100.00	2,905<br>100.00<br>100.00

Pearson chi2(**1**) = **1.1280** Pr = **0.288**

-> bysort study_location: tab cort_prev gender, r col chi nokey

-> study_location = Delhi(1)

cort_prev	gender Male	Female	Total
NormOrNotcort	**1,279**	**1,540**	**2,819**
	**45.37**	**54.63**	**100.00**
	**88.88**	**88.97**	**88.93**
cort	**160**	**191**	**351**
	**45.58**	**54.42**	**100.00**
	**11.12**	**11.03**	**11.07**
Total	**1,439**	**1,731**	**3,170**
	**45.39**	**54.61**	**100.00**
	**100.00**	**100.00**	**100.00**

Pearson chi2(**1**) = **0.0057** Pr = **0.940**

-> study_location = Guwahati(2)

cort_prev	gender Male	Female	Total
NormOrNotcort	**1,327**	**1,515**	**2,842**
	**46.69**	**53.31**	**100.00**
	**92.80**	**91.54**	**92.12**
cort	**103**	**140**	**243**
	**42.39**	**57.61**	**100.00**
	**7.20**	**8.46**	**7.88**
Total	**1,430**	**1,655**	**3,085**
	**46.35**	**53.65**	**100.00**
	**100.00**	**100.00**	**100.00**

Pearson chi2(**1**) = **1.6689** Pr = **0.196**

-> study_location = Prakasam(3)

cort_prev	gender Male	Female	Total
NormOrNotcort	**1,197**	**1,374**	**2,571**
	**46.56**	**53.44**	**100.00**
	**99.01**	**99.42**	**99.23**
cort	**12**	**8**	**20**
	**60.00**	**40.00**	**100.00**
	**0.99**	**0.58**	**0.77**
Total	**1,209**	**1,382**	**2,591**
	**46.66**	**53.34**	**100.00**
	**100.00**	**100.00**	**100.00**

Pearson chi2(**1**) = **1.4408** Pr = **0.230**

-> bysort study_location: tab nuc_prev gender, r col chi nokey

-> study_location = Delhi(1)

nuc_prev	gender Male	Female	Total
NormOrNotnuc	1,164<br>45.26<br>80.89	1,408<br>54.74<br>81.34	2,572<br>100.00<br>81.14
nuc	275<br>45.99<br>19.11	323<br>54.01<br>18.66	598<br>100.00<br>18.86
Total	1,439<br>45.39<br>100.00	1,731<br>54.61<br>100.00	3,170<br>100.00<br>100.00

Pearson chi2(**1**) = **0.1043** Pr = **0.747**

-> study_location = Guwahati(2)

nuc_prev	gender Male	Female	Total
NormOrNotnuc	1,151<br>46.69<br>80.49	1,314<br>53.31<br>79.40	2,465<br>100.00<br>79.90
nuc	279<br>45.00<br>19.51	341<br>55.00<br>20.60	620<br>100.00<br>20.10
Total	1,430<br>46.35<br>100.00	1,655<br>53.65<br>100.00	3,085<br>100.00<br>100.00

Pearson chi2(**1**) = **0.5715** Pr = **0.450**

-> study_location = Prakasam(3)

nuc_prev	gender Male	Female	Total
NormOrNotnuc	879<br>46.83<br>72.70	998<br>53.17<br>72.21	1,877<br>100.00<br>72.44
nuc	330<br>46.22<br>27.30	384<br>53.78<br>27.79	714<br>100.00<br>27.56
Total	1,209<br>46.66<br>100.00	1,382<br>53.34<br>100.00	2,591<br>100.00<br>100.00

Pearson chi2(**1**) = **0.0777** Pr = **0.780**

-> bysort study_location: tab psc_prev gender, r col chi nokey

-> study_location = Delhi(1)

psc_prev	gender Male	Female	Total
NormOrNotpsc	1,287	1,559	2,846
	45.22	54.78	100.00
	89.44	90.06	89.78
psc	152	172	324
	46.91	53.09	100.00
	10.56	9.94	10.22
Total	1,439	1,731	3,170
	45.39	54.61	100.00
	100.00	100.00	100.00

Pearson chi2(**1**) = **0.3360** Pr = **0.562**

-> study_location = Guwahati(2)

psc_prev	gender Male	Female	Total
NormOrNotpsc	1,407	1,624	3,031
	46.42	53.58	100.00
	98.39	98.13	98.25
psc	23	31	54
	42.59	57.41	100.00
	1.61	1.87	1.75
Total	1,430	1,655	3,085
	46.35	53.65	100.00
	100.00	100.00	100.00

Pearson chi2(**1**) = **0.3126** Pr = **0.576**

-> study_location = Prakasam(3)

psc_prev	gender Male	Female	Total
NormOrNotpsc	1,196	1,362	2,558
	46.76	53.24	100.00
	98.92	98.55	98.73
psc	13	20	33
	39.39	60.61	100.00
	1.08	1.45	1.27
Total	1,209	1,382	2,591
	46.66	53.34	100.00
	100.00	100.00	100.00

Pearson chi2(**1**) = **0.7094** Pr = **0.400**

-> bysort study_location: tab cort_assoc gender, r col chi nokey

---

-> study_location = Delhi(1)

cort_assoc	gender Male	Female	Total
0	1,119<br>45.40<br>99.56	1,346<br>54.60<br>99.48	2,465<br>100.00<br>99.52
Cort	5<br>41.67<br>0.44	7<br>58.33<br>0.52	12<br>100.00<br>0.48
Total	1,124<br>45.38<br>100.00	1,353<br>54.62<br>100.00	2,477<br>100.00<br>100.00

Pearson chi2(**1**) = **0.0670** Pr = **0.796**

-> study_location = Guwahati(2)

cort_assoc	gender Male	Female	Total
0	1,118<br>46.82<br>99.55	1,270<br>53.18<br>99.61	2,388<br>100.00<br>99.58
Cort	5<br>50.00<br>0.45	5<br>50.00<br>0.39	10<br>100.00<br>0.42
Total	1,123<br>46.83<br>100.00	1,275<br>53.17<br>100.00	2,398<br>100.00<br>100.00

Pearson chi2(**1**) = **0.0405** Pr = **0.840**

-> study_location = Prakasam(3)

cort_assoc	gender Male	Female	Total
0	835<br>46.73<br>98.82	952<br>53.27<br>99.37	1,787<br>100.00<br>99.11
Cort	10<br>62.50<br>1.18	6<br>37.50<br>0.63	16<br>100.00<br>0.89
Total	845<br>46.87<br>100.00	958<br>53.13<br>100.00	1,803<br>100.00<br>100.00

Pearson chi2(**1**) = **1.5845** Pr = **0.208**

-> bysort study_location: tab nuc_assoc gender, r col chi nokey

---

-> study_location = Delhi(1)

nuc_assoc	gender Male	Female	Total
0	1,119	1,346	2,465
	45.40	54.60	100.00
	93.41	93.93	93.69
Nuc	79	87	166
	47.59	52.41	100.00
	6.59	6.07	6.31
Total	1,198	1,433	2,631
	45.53	54.47	100.00
	100.00	100.00	100.00

Pearson chi2(**1**) = **0.3021** Pr = **0.583**

-> study_location = Guwahati(2)

nuc_assoc	gender Male	Female	Total
0	1,118	1,270	2,388
	46.82	53.18	100.00
	87.28	87.23	87.25
Nuc	163	186	349
	46.70	53.30	100.00
	12.72	12.77	12.75
Total	1,281	1,456	2,737
	46.80	53.20	100.00
	100.00	100.00	100.00

Pearson chi2(**1**) = **0.0015** Pr = **0.969**

-> study_location = Prakasam(3)

nuc_assoc	gender Male	Female	Total
0	835	952	1,787
	46.73	53.27	100.00
	72.17	72.07	72.11
Nuc	322	369	691
	46.60	53.40	100.00
	27.83	27.93	27.89
Total	1,157	1,321	2,478
	46.69	53.31	100.00
	100.00	100.00	100.00

Pearson chi2(**1**) = **0.0032** Pr = **0.955**

-> bysort study_location: tab psc_assoc gender, r col chi nokey

-> study_location = Delhi(1)

psc_assoc	gender Male	Female	Total
0	1,119<br>45.40<br>98.94	1,346<br>54.60<br>99.26	2,465<br>100.00<br>99.12
Psc	12<br>54.55<br>1.06	10<br>45.45<br>0.74	22<br>100.00<br>0.88
Total	1,131<br>45.48<br>100.00	1,356<br>54.52<br>100.00	2,487<br>100.00<br>100.00

Pearson chi2(**1**) = **0.7363** Pr = **0.391**

-> study_location = Guwahati(2)

psc_assoc	gender Male	Female	Total
0	1,118<br>46.82<br>99.38	1,270<br>53.18<br>99.14	2,388<br>100.00<br>99.25
Psc	7<br>38.89<br>0.62	11<br>61.11<br>0.86	18<br>100.00<br>0.75
Total	1,125<br>46.76<br>100.00	1,281<br>53.24<br>100.00	2,406<br>100.00<br>100.00

Pearson chi2(**1**) = **0.4511** Pr = **0.502**

-> study_location = Prakasam(3)

psc_assoc	gender Male	Female	Total
0	835<br>46.73<br>98.70	952<br>53.27<br>98.35	1,787<br>100.00<br>98.51
Psc	11<br>40.74<br>1.30	16<br>59.26<br>1.65	27<br>100.00<br>1.49
Total	846<br>46.64<br>100.00	968<br>53.36<br>100.00	1,814<br>100.00<br>100.00

Pearson chi2(**1**) = **0.3829** Pr = **0.536**

-> bysort study_location: tab pure_assoc gender, r col chi nokey

-> study_location = Delhi(1)

pure_assoc	gender Male	Female	Total
0	1,119<br>45.40<br>92.10	1,346<br>54.60<br>92.83	2,465<br>100.00<br>92.50
1	5<br>41.67<br>0.41	7<br>58.33<br>0.48	12<br>100.00<br>0.45
2	79<br>47.59<br>6.50	87<br>52.41<br>6.00	166<br>100.00<br>6.23
3	12<br>54.55<br>0.99	10<br>45.45<br>0.69	22<br>100.00<br>0.83
Total	1,215<br>45.59<br>100.00	1,450<br>54.41<br>100.00	2,665<br>100.00<br>100.00

Pearson chi2(**3**) = **1.0911** Pr = **0.779**

-> study_location = Guwahati(2)

pure_assoc	gender Male	Female	Total
0	1,118<br>46.82<br>86.47	1,270<br>53.18<br>86.28	2,388<br>100.00<br>86.37
1	5<br>50.00<br>0.39	5<br>50.00<br>0.34	10<br>100.00<br>0.36
2	163<br>46.70<br>12.61	186<br>53.30<br>12.64	349<br>100.00<br>12.62
3	7<br>38.89<br>0.54	11<br>61.11<br>0.75	18<br>100.00<br>0.65
Total	1,293<br>46.76<br>100.00	1,472<br>53.24<br>100.00	2,765<br>100.00<br>100.00

Pearson chi2(**3**) = **0.4937** Pr = **0.920**

-> study_location = Prakasam(3)

pure_assoc	gender Male	Female	Total
0	835<br>46.73<br>70.88	952<br>53.27<br>70.89	1,787<br>100.00<br>70.88
1	10<br>62.50<br>0.85	6<br>37.50<br>0.45	16<br>100.00<br>0.63
2	322<br>46.60<br>27.33	369<br>53.40<br>27.48	691<br>100.00<br>27.41
3	11	16	27

	40.74	59.26	100.00
	0.93	1.19	1.07
Total	1,178	1,343	2,521
	46.73	53.27	100.00
	100.00	100.00	100.00

Pearson chi2(**3**) = **1.9923** Pr = **0.574**

149
150 cap log close
