# Supplementary material for: Association of cataract and sun exposure in geographically diverse populations of India: The CASE study. First Report of the ICMR-EYE SEE Study Group
Source: PLoS One. 2020 Jan 23;15(1):e0227868. doi: 10.1371/journal.pone.0227868 (PMC6977762; doi:10.1371/journal.pone.0227868)
Supplement: S2 File — The study dataset in Stata and spreadsheet formats, codebooks, analysis commands, output logs and instructions for using the dataset. (ZIP) [file pone.0227868.s002.zip › UVR-cataract-analysis - Codebook.pdf]

```
      name:  <unnamed>
       log:  F:\Dropbox\VIVEK\paper\UVR Ctaract\PlOS Submission\R1\dataR1\UVR-cataract
> -analysis\codebook.smcl
  log type:  smcl
 opened on:   6 Jan 2020, 12:03:27

1 . codebook

-------------------------------------------------------------------------------------
unique_id                                                               (unlabeled)
-------------------------------------------------------------------------------------

                  type:  numeric (long)

                 range:  [10100101,33509702]         units:  1
         unique values:  9,735                   missing .:  0/9,735

                  mean:   2.1e+07
             std. dev:   8.2e+06

           percentiles:        10%       25%       50%       75%       90%
                            1.1e+07   1.3e+07   2.1e+07   3.1e+07   3.2e+07

-------------------------------------------------------------------------------------
study_location                                                          (unlabeled)
-------------------------------------------------------------------------------------

                  type:  numeric (byte)
                 label:  locationlb

                 range:  [1,3]                       units:  1
         unique values:  3                       missing .:  0/9,735

            tabulation:  Freq.   Numeric  Label
                         3,595         1  Delhi(1)
                         3,231         2  Guwahati(2)
                         2,909         3  Prakasam(3)

-------------------------------------------------------------------------------------
agecat                                                               RECODE of age
-------------------------------------------------------------------------------------

                  type:  numeric (int)
                 label:  agecatlb

                 range:  [4,7]                       units:  1
         unique values:  4                       missing .:  0/9,735

            tabulation:  Freq.   Numeric  Label
                         3,998         4  40-49 years
                         2,438         5  50-59 years
                         1,981         6  60-69 years
                         1,318         7  70+ years

-------------------------------------------------------------------------------------
gender                                                                  (unlabeled)
-------------------------------------------------------------------------------------

                  type:  numeric (int)
                 label:  genderlb

                 range:  [1,2]                       units:  1
         unique values:  2                       missing .:  0/9,735

            tabulation:  Freq.   Numeric  Label
                         4,426         1  Male
                         5,309         2  Female
```

```
------------------------------------------------------------------------------
tExposure                                                          (unlabeled)
------------------------------------------------------------------------------

                  type:  numeric (float)

                 range:  [7305,314104.03]             units:  .0001
         unique values:  4,724                    missing .:  5/9,735

                  mean:    100565
              std. dev:   47624.5

           percentiles:        10%       25%       50%       75%       90%
                             45291   66393.3   96062.6    131081    166145

------------------------------------------------------------------------------
smokepackTotCat2                                     RECODE of smokepackTotal
------------------------------------------------------------------------------

                  type:  numeric (float)
                 label:  smokepackTotCat2, but 1 nonmissing value is not labeled

                 range:  [0,99]                       units:  1
         unique values:  5                        missing .:  0/9,735

            tabulation:  Freq.   Numeric  Label
                         6,149         0  NonSmoker
                           494         1  >0 to <=1 pack
                         1,090         2  >1 to <5 pack
                         1,659         3  >=5 pack
                           343        99

------------------------------------------------------------------------------
sugar200                                                           (unlabeled)
------------------------------------------------------------------------------

                  type:  numeric (float)

                 range:  [0,1]                        units:  1
         unique values:  2                        missing .:  161/9,735

            tabulation:  Freq.  Value
                         8,742  0
                           832  1
                           161  .

------------------------------------------------------------------------------
age                                                                (unlabeled)
------------------------------------------------------------------------------

                  type:  numeric (int)

                 range:  [40,99]                      units:  1
         unique values:  60                       missing .:  0/9,735

                  mean:   54.4649
              std. dev:   11.6576

           percentiles:        10%       25%       50%       75%       90%
                                41        45        52        62        70

------------------------------------------------------------------------------
ocptncat                                         RECODE of current_occupation
------------------------------------------------------------------------------

                  type:  numeric (int)
                 label:  ocptncatlb

                 range:  [1,5]                        units:  1
         unique values:  5                        missing .:  0/9,735
```

tabulation:

Freq.	Numeric	Label
3,711	1	House work
3,392	2	Unskilled
1,115	3	Skilled
1,508	4	Unemployed
9	5	Others

---

indoor16_kitchen **(unlabeled)**

---

type: numeric (**byte**)
label: **ynlb2**, but **1** nonmissing value is not labeled

range: [**0**,**1**]   units: **1**
unique values: **2**   missing .: **0**/**9,735**

tabulation:

Freq.	Numeric	Label
2,801	0	
6,934	1	Yes

---

smoke20 **(unlabeled)**

---

type: numeric (**byte**)
label: **ynlb2**, but **1** nonmissing value is not labeled

range: [**0**,**1**]   units: **1**
unique values: **2**   missing .: **6**/**9,735**

tabulation:

Freq.	Numeric	Label
6,144	0	
3,585	1	Yes
6	.	

---

educat2 **RECODE of education**

---

type: numeric (**int**)
label: **educat2**, but **1** nonmissing value is not labeled

range: [**0**,**99**]   units: **1**
unique values: **7**   missing .: **0**/**9,735**

tabulation:

Freq.	Numeric	Label
5,000	0	Illiterate
1,798	1	Can read & write
934	2	Middle School (6-8)
1,725	3	High School (9-12)
268	4	Graduation
1	5	Others
9	99	

---

workTypePrCat **(unlabeled)**

---

type: numeric (**float**)
label: **workTypelb**

range: [**1**,**2**]   units: **1**
unique values: **2**   missing .: **13**/**9,735**

tabulation:

Freq.	Numeric	Label
1,733	1	Indoor work
7,989	2	Outdoor work
13	.	

tExposureQuantilesAll — **5 quantiles of tExposure**

```
            type:  numeric (byte)

           range:  [1,5]                        units:  1
   unique values:  5                        missing .:  5/9,735

      tabulation:  Freq.  Value
                   1,947  1
                   1,945  2
                   1,947  3
                   1,948  4
                   1,943  5
                       5  .
```

smokepackTotCat3 — **RECODE of smokepackTotal**

```
            type:  numeric (float)
           label:  smokepackTotCat3, but 1 nonmissing value is not labeled

           range:  [0,99]                       units:  1
   unique values:  7                        missing .:  0/9,735

      tabulation:  Freq.   Numeric  Label
                   6,149         0  NonSmoker
                     838         1  >0 to 2 pack
                     802         2  >2 to 5 pack
                     555         3  >5 to 10 pack
                     482         4  >10 to 25 pack
                     566         5  >=25 pack
                     343        99
```

lens_re — **(unlabeled)**

```
            type:  numeric (float)
           label:  lens

           range:  [0,9]                        units:  1
   unique values:  6                        missing .:  0/9,735

      tabulation:  Freq.   Numeric  Label
                   6,586         0  0.Normal
                   2,004         1  1.Co/Nu/Psc
                     133         2  2.Advanced
                      10         7  7.Tr/Dev./Dis.
                     935         8  8.Operated
                      67         9  9.Undet
```

lens_le — **(unlabeled)**

```
            type:  numeric (float)
           label:  lens

           range:  [0,9]                        units:  1
   unique values:  6                        missing .:  0/9,735

      tabulation:  Freq.   Numeric  Label
                   6,598         0  0.Normal
                   2,051         1  1.Co/Nu/Psc
                     140         2  2.Advanced
                       9         7  7.Tr/Dev./Dis.
                     882         8  8.Operated
                      55         9  9.Undet
```

```
-------------------------------------------------------------------------------------
cort_locs_re                                                              (unlabeled)
-------------------------------------------------------------------------------------

                  type:  numeric (float)
                 label:  cort, but 1 nonmissing value is not labeled

                 range:  [0,1]                        units:  1
         unique values:  2                        missing .:  1,208/9,735

            tabulation:  Freq.   Numeric  Label
                         8,028         0
                           499         1  Cort
                         1,208         .

-------------------------------------------------------------------------------------
nuc_locs_re                                                               (unlabeled)
-------------------------------------------------------------------------------------

                  type:  numeric (float)
                 label:  nuc, but 1 nonmissing value is not labeled

                 range:  [0,1]                        units:  1
         unique values:  2                        missing .:  1,208/9,735

            tabulation:  Freq.   Numeric  Label
                         6,893         0
                         1,634         1  Nuc
                         1,208         .

-------------------------------------------------------------------------------------
psc_locs_re                                                               (unlabeled)
-------------------------------------------------------------------------------------

                  type:  numeric (float)
                 label:  psc, but 1 nonmissing value is not labeled

                 range:  [0,1]                        units:  1
         unique values:  2                        missing .:  1,208/9,735

            tabulation:  Freq.   Numeric  Label
                         8,231         0
                           296         1  Psc
                         1,208         .

-------------------------------------------------------------------------------------
cort_locs_le                                                              (unlabeled)
-------------------------------------------------------------------------------------

                  type:  numeric (float)
                 label:  cort, but 1 nonmissing value is not labeled

                 range:  [0,1]                        units:  1
         unique values:  2                        missing .:  1,163/9,735

            tabulation:  Freq.   Numeric  Label
                         8,090         0
                           482         1  Cort
                         1,163         .

-------------------------------------------------------------------------------------
nuc_locs_le                                                               (unlabeled)
-------------------------------------------------------------------------------------

                  type:  numeric (float)
                 label:  nuc, but 1 nonmissing value is not labeled

                 range:  [0,1]                        units:  1
         unique values:  2                        missing .:  1,163/9,735
```

```
          tabulation:  Freq.   Numeric  Label
                       6,915         0
                       1,657         1  Nuc
                       1,163         .
```

---

psc_locs_le **(unlabeled)**

```
                type:  numeric (float)
               label:  psc, but 1 nonmissing value is not labeled

               range:  [0,1]                        units:  1
       unique values:  2                        missing .:  1,163/9,735

          tabulation:  Freq.   Numeric  Label
                       8,287         0
                         285         1  Psc
                       1,163         .
```

---

anyCataract **(unlabeled)**

```
                type:  numeric (float)

               range:  [0,1]                        units:  1
       unique values:  2                        missing .:  19/9,735

          tabulation:  Freq.  Value
                       6,485  0
                       3,231  1
                          19  .
```

---

beOp **(unlabeled)**

```
                type:  numeric (float)

               range:  [0,1]                        units:  1
       unique values:  2                        missing .:  19/9,735

          tabulation:  Freq.  Value
                       9,122  0
                         594  1
                          19  .
```

---

oneEyeOp **(unlabeled)**

```
                type:  numeric (float)

               range:  [0,1]                        units:  1
       unique values:  2                        missing .:  19/9,735

          tabulation:  Freq.  Value
                       9,087  0
                         629  1
                          19  .
```

---

locsRe_cort **(unlabeled)**

```
                type:  numeric (float)
               label:  locs_cort, but 1 nonmissing value is not labeled

               range:  [0,9]                        units:  1
       unique values:  6                        missing .:  19/9,735
```

```
     tabulation:  Freq.   Numeric  Label
                  8,028         0
                    499         1  Cort
                    133         3  3.Adv
                      6         7  7.Tr.
                    935         8  8.Op
                    115         9  9.Mis
                     19         .
```

---

locsLe_cort **(unlabeled)**

```
           type:  numeric (float)
          label:  locs_cort, but 1 nonmissing value is not labeled

          range:  [0,9]                        units:  1
  unique values:  6                        missing .:  19/9,735

     tabulation:  Freq.   Numeric  Label
                  8,090         0
                    482         1  Cort
                    140         3  3.Adv
                      5         7  7.Tr.
                    882         8  8.Op
                    117         9  9.Mis
                     19         .
```

---

excludeLocs **(unlabeled)**

```
           type:  numeric (float)

          range:  [0,1]                        units:  1
  unique values:  2                        missing .:  19/9,735

     tabulation:  Freq.  Value
                  8,846  0
                    870  1
                     19  .
```

---

cort_prev **(unlabeled)**

```
           type:  numeric (float)
          label:  cort_prev

          range:  [0,1]                        units:  1
  unique values:  2                        missing .:  889/9,735

     tabulation:  Freq.   Numeric  Label
                  8,232         0  NormOrNotcort
                    614         1  cort
                    889         .
```

---

nuc_prev **(unlabeled)**

```
           type:  numeric (float)
          label:  nuc_prev

          range:  [0,1]                        units:  1
  unique values:  2                        missing .:  889/9,735

     tabulation:  Freq.   Numeric  Label
                  6,914         0  NormOrNotnuc
                  1,932         1  nuc
                    889         .
```

psc_prev (unlabeled)

```
              type:  numeric (float)
             label:  psc_prev

             range:  [0,1]                        units:  1
     unique values:  2                        missing .:  889/9,735

        tabulation:  Freq.   Numeric  Label
                     8,435         0  NormOrNotpsc
                       411         1  psc
                       889         .
```

pvaBe (unlabeled)

```
              type:  numeric (float)

             range:  [1,5]                        units:  1
     unique values:  5                        missing .:  59/9,735

        tabulation:  Freq.  Value
                     6,760  1
                       754  2
                     1,709  3
                       112  4
                       341  5
                        59  .
```

bvaBe (unlabeled)

```
              type:  numeric (float)

             range:  [1,5]                        units:  1
     unique values:  5                        missing .:  59/9,735

        tabulation:  Freq.  Value
                     8,483  1
                       295  2
                       547  3
                        72  4
                       279  5
                        59  .
```

pattern_re group(cort_locs_re nuc_locs_re psc_locs_re)

```
              type:  numeric (float)
             label:  pattern_re

             range:  [1,8]                        units:  1
     unique values:  8                        missing .:  1,208/9,735

        tabulation:  Freq.   Numeric  Label
                     6,798         1  0 0 0
                        50         2  0 0 Psc
                     1,097         3  0 Nuc 0
                        83         4  0 Nuc Psc
                        38         5  Cort 0 0
                         7         6  Cort 0 Psc
                       298         7  Cort Nuc 0
                       156         8  Cort Nuc Psc
                     1,208         .
```

---

pattern_le **group(cort_locs_le nuc_locs_le psc_locs_le)**

```
             type:  numeric (float)
            label:  pattern_le

            range:  [1,8]                        units:  1
    unique values:  8                        missing .:  1,163/9,735

      tabulation:  Freq.   Numeric  Label
                   6,826         1  0 0 0
                      51         2  0 0 Psc
                   1,129         3  0 Nuc 0
                      84         4  0 Nuc Psc
                      33         5  Cort 0 0
                       5         6  Cort 0 Psc
                     299         7  Cort Nuc 0
                     145         8  Cort Nuc Psc
                   1,163         .
```

---

cort_assoc **(unlabeled)**

```
             type:  numeric (float)
            label:  cort, but 1 nonmissing value is not labeled

            range:  [0,1]                        units:  1
    unique values:  2                        missing .:  3,057/9,735

      tabulation:  Freq.   Numeric  Label
                   6,640         0
                      38         1  Cort
                   3,057         .
```

---

nuc_assoc **(unlabeled)**

```
             type:  numeric (float)
            label:  nuc, but 1 nonmissing value is not labeled

            range:  [0,1]                        units:  1
    unique values:  2                        missing .:  1,889/9,735

      tabulation:  Freq.   Numeric  Label
                   6,640         0
                   1,206         1  Nuc
                   1,889         .
```

---

psc_assoc **(unlabeled)**

```
             type:  numeric (float)
            label:  psc, but 1 nonmissing value is not labeled

            range:  [0,1]                        units:  1
    unique values:  2                        missing .:  3,028/9,735

      tabulation:  Freq.   Numeric  Label
                   6,640         0
                      67         1  Psc
                   3,028         .
```

---

pure_assoc **(unlabeled)**

```
             type:  numeric (float)
```

```
                  range:  [0,3]                        units:  1
          unique values:  4                        missing .:  1,784/9,735

             tabulation:  Freq.  Value
                          6,640  0
                             38  1
                          1,206  2
                             67  3
                          1,784  .

2 . log close
      name:  <unnamed>
       log:  F:\Dropbox\VIVEK\paper\UVR Ctaract\PlOS Submission\R1\dataR1\UVR-cataract
  > -analysis\codebook.smcl
  log type:  smcl
 closed on:   6 Jan 2020, 12:03:35
```
