# Supplementary material for: Association of cataract and sun exposure in geographically diverse populations of India: The CASE study. First Report of the ICMR-EYE SEE Study Group
Source: PLoS One. 2020 Jan 23;15(1):e0227868. doi: 10.1371/journal.pone.0227868 (PMC6977762; doi:10.1371/journal.pone.0227868)
Supplement: Attachment — Submitted filename: Response to reviewers.docx [file pone.0227868.s003.docx]

**Comments from the Editor**
Thank you for submitting your manuscript to PLOS ONE. After careful consideration, we feel that it has merit but does not fully meet PLOS ONE’s publication criteria as it currently stands. Therefore, we invite you to submit a revised version of the manuscript that addresses the points raised during the review process. We would appreciate receiving your revised manuscript by Dec 16 2019 11:59PM. When you are ready to submit your revision, log on to <https://www.editorialmanager.com/pone/> and select the 'Submissions Needing Revision' folder to locate your manuscript file.

If you would like to make changes to your financial disclosure, please include your updated statement in your cover letter.

To enhance the reproducibility of your results, we recommend that if applicable you deposit your laboratory protocols in [protocols.io](http://protocols.io), where a protocol can be assigned its own identifier (DOI) such that it can be cited independently in the future. For instructions see: <http://journals.plos.org/plosone/s/submission-guidelines#loc-laboratory-protocols>

Please include the following items when submitting your revised manuscript:

- A rebuttal letter that responds to each point raised by the academic editor and reviewer(s). This letter should be uploaded as separate file and labeled 'Response to Reviewers'.
- A marked-up copy of your manuscript that highlights changes made to the original version. This file should be uploaded as separate file and labeled 'Revised Manuscript with Track Changes'.
- An unmarked version of your revised paper without tracked changes. This file should be uploaded as separate file and labeled 'Manuscript'.
- Please note while forming your response, if your article is accepted, you may have the opportunity to make the peer review history publicly available. The record will include editor decision letters (with reviews) and your responses to reviewer comments. If eligible, we will contact you to opt in or out.

We look forward to receiving your revised manuscript.
Kind regards,
James Wolffsohn, PhD
Academic Editor
PLOS ONE

**Authors’ Response:** Thank you for the kind consideration and opportunity provided to revise the document. We have followed the instructions and are submitting the revised version for reconsideration

**Journal Requirements:**

1. When submitting your revision, we need you to address these additional requirements. Please ensure that your manuscript meets PLOS ONE's style requirements, including those for file naming. The PLOS ONE style templates can be found at <http://www.journals.plos.org/plosone/s/file?id=wjVg/PLOSOne_formatting_sample_main_body.pdf> and <http://www.journals.plos.org/plosone/s/file?id=ba62/PLOSOne_formatting_sample_title_authors_affiliations.pdf>

**Authors’ Response** –The file has been edited to reflect the journal requirements.

2. Please include additional information regarding the survey or questionnaire used in the study and ensure that you have provided sufficient details that others could replicate the analyses. For instance, if you developed a questionnaire as part of this study and it is not under a copyright license more restrictive than CC-BY, please include a copy, in both the original language and English, as Supporting Information.

**Authors’ Response** – The questionnaire has been included as Additional/Supporting Information. (Page 31)

3. We note that you have indicated that data from this study are available upon request. PLOS only allows data to be available upon request if there are legal or ethical restrictions on sharing data publicly. For information on unacceptable data access restrictions, please see <http://journals.plos.org/plosone/s/data-availability#loc-unacceptable-data-access-restrictions>.
In your revised cover letter, please address the following prompts:
a) If there are ethical or legal restrictions on sharing a de-identified data set, please explain them in detail (e.g., data contain potentially identifying or sensitive patient information) and who has imposed them (e.g., an ethics committee). Please also provide contact information for a data access committee, ethics committee, or other institutional body to which data requests may be sent.
b) If there are no restrictions, please upload the minimal anonymized data set necessary to replicate your study findings as either Supporting Information files or to a stable, public repository and provide us with the relevant URLs, DOIs, or accession numbers. Please see <http://www.bmj.com/content/340/bmj.c181.long> for guidelines on how to de-identify and prepare clinical data for publication. For a list of acceptable repositories, please see <http://journals.plos.org/plosone/s/data-availability#loc-recommended-repositories>.
We will update your Data Availability statement on your behalf to reflect the information you provide.

**Authors’ Response** – The data has been submitted to the DRYAD repository. The information is Under Peer Review with the following Citation -

*Vashist, Praveen et al. (2019), Association of Cataract and Sun Exposure in geographically diverse populations of India: The CASE study. First Report of the ICMR-EYE SEE Study Group, v2, Dryad, Dataset,* [*https://doi.org/10.5061/dryad.5qfttdz19*](https://doi.org/10.5061/dryad.5qfttdz19)

4. Please amend either the title on the online submission form (via Edit Submission) or the title in the manuscript so that they are identical.

**Authors’ Response** – The title has been amended to be identical at both places as follows:

**Association of Cataract and Sun Exposure in geographically diverse populations of India: The CASE study. First Report of the ICMR-EYE SEE Study Group**

5. Please amend the manuscript submission data (via Edit Submission) to include authors GVS Murthy , CK Barua , Dipali Deka , Sachchidanand Singh, Vivek Gupta , Noopur Gupta , Meenakshi Wadhwani , Rashmi Singh and K Vishwanath

**Authors’ Response** – The manuscript submission data has been amended to include authors GVS Murthy, CK Barua, Dipali Deka, Sachchidanand Singh, Vivek Gupta, Noopur Gupta, Meenakshi Wadhwani, Rashmi Singh and K Vishwanath.

6. One of the noted authors is a group or consortium: ICMR-EYE SEE Study Group. In addition to naming the author group, please list the individual authors and affiliations within this group in the acknowledgments section of your manuscript. Please also indicate clearly a lead author for this group along with a contact email address.

**Authors’ Response** – We have added the information in the acknowledgments section of the manuscript and indicated clearly a lead author along with a contact email address (page 26) in acknowledgments and on title page.

**Additional Editor Comments:**

Well done on putting together such a good manuscript. Please could you address the minor comments and resubmit

**Authors’ Response- We are grateful for the encouraging observation. We have addressed the comments in the revised version.**

[Note: HTML markup is below. Please do not edit.]

Reviewers' comments:

Reviewer's Responses to Questions

**Comments to the Author**

1. Is the manuscript technically sound, and do the data support the conclusions?

The manuscript must describe a technically sound piece of scientific research with data that supports the conclusions. Experiments must have been conducted rigorously, with appropriate controls, replication, and sample sizes. The conclusions must be drawn appropriately based on the data presented.

Reviewer #1: Yes

Reviewer #2: Yes

2. Has the statistical analysis been performed appropriately and rigorously?

Reviewer #1: Yes

Reviewer #2: I Don't Know

3. Have the authors made all data underlying the findings in their manuscript fully available?

The [PLOS Data policy](http://www.plosone.org/static/policies.action#sharing) requires authors to make all data underlying the findings described in their manuscript fully available without restriction, with rare exception (please refer to the Data Availability Statement in the manuscript PDF file). The data should be provided as part of the manuscript or its supporting information, or deposited to a public repository. For example, in addition to summary statistics, the data points behind means, medians and variance measures should be available. If there are restrictions on publicly sharing data—e.g. participant privacy or use of data from a third party—those must be specified.

Reviewer #1: Yes

Reviewer #2: Yes

4. Is the manuscript presented in an intelligible fashion and written in standard English?

PLOS ONE does not copyedit accepted manuscripts, so the language in submitted articles must be clear, correct, and unambiguous. Any typographical or grammatical errors should be corrected at revision, so please note any specific errors here.

Reviewer #1: Yes

Reviewer #2: Yes

5. Review Comments to the Author

Please use the space provided to explain your answers to the questions above. You may also include additional comments for the author, including concerns about dual publication, research ethics, or publication ethics. (Please upload your review as an attachment if it exceeds 20,000 characters)

**Reviewer #1: Summary & Overall Impression**

This was a very large, comprehensive and well conducted study of the relationship of sun exposure in India. The authors were able to amass a large sample of over 12,000 subjects spread relatively evenly across 3 distinct geographic areas of India, and to collect clinical and subjective data on them. They present their data in a clear and logical fashion, and have employed adequate statistical rigor to their work. Their conclusion that cataracts were associated with increasing levels of sun exposure, smoking and in some cases, exposure to indoor kitchen smoke is consistent with other published studies, and they have pointed out nuances relevant to the population and conditions in India. I find this to be a relevant and important addition to the literature, and recommend that it be published after addressing some minor issues with consistency in the paper.

**Authors’ Response** – We thank the kind reviewer for the encouraging words

**Issues to be addressed (minor)**
**Comment 1:** At line 311, they discuss noting increasing prevalence in cataract with decreasing distance to the equator. However, their data in Table 1 shows that while the site closest to the equator (Prakasam at ~15 degrees N) had the highest rate of cataract reported (42.4%), the site at ~28 degrees N (Guwahati - 31.9%) actually had a higher rate than the site at ~26 degrees N (Gurgaon - 26.6%). Additionally, when looking at the total UV exposures reported in Table 2, neither is the median lifetime UV exposure data consistent with the rates of cataracts. The authors should discuss additional potential factors such as work on water, increase in UV exposure at higher elevations and differences due to living and working in proximity to an urban center vs. a more rural location.

**Authors’ Response** – Thank you for the pertinent observations. We agree with the reviewer’s comments. These are important issues and we have addressed them by modifying the manuscript as explained below:

LINES 316-323*: The site closest to the equator (Prakasam, 15.4 degrees N; 42.4%) had the highest rate of cataract reported than the other two sites which were located at similar latitudes (Guwahati, 26.1 degrees N; 26.6%, Delhi, 28.7 degrees N; 31.9%) and between the latter two, the rate was higher at Delhi than Guwahati. ……. The present study highlights that while latitude is indeed an important factor, there are other factors that influence development of cataract as well.*

Additionally, when relating the total UV exposure and rate of cataract, the lack of consistency has been attended to by further discussion points which have been added to address the lack of consistency between lifetime sun exposure and rates of cataract and mention additional potential factors to this effect, including elevation, work on water etc. The following text has been added to the manuscript:

LINES 324-330*:* *What is interesting is that the median lifetime sun exposure was higher in Delhi (114140) than in Prakasam (109889), and in both of these sites it was nearly one and a half times that in Guwahati (72759).* *This suggests that disparities in the nature of sun exposure such as work on water, variations in UV exposure at different elevations, and further differences due to living and working in proximity to an urban centre versus a more rural location associated with each geographical site have additional influences and roles to play in development of cataract.*

**Comment 2:** At line 328, the authors cite 2 studies discussing the association with sun exposure and cataract, but fail to mention a very important environmental factor - *working over water*, as was the case with Hong Kong fisherman and the waterman of Chesapeake Bay. In the paper, which the authors have cited as a basis of their UV exposure calculations, McCarty shows a LARGE influence of water (a factor of 1.9 applied to over water exposure times). This may be an interesting factors for the authors to characterize in future studies, particularly in the regions with ocean and large river influences. This comes up again at line 347.

**Authors’ Response** – We appreciate the significance of this comment. Accordingly, we have added discussion to this effect and also recognize this as a future research recommendation. The following lines have been added in the discussion

LINES 425-432*: Work on water, which is reported to have a large impact on effective sun exposure and recognized as an important risk factor for cataract [6,30,31], could be an additional contributory factor in Prakasam, a coastal district. This aspect needs further exploration in future studies. Based on the results, it is recommended future studies should try to get in-depth information on nature of headgear used and the time of day when used, duration of work on water, and ascertain individual level UV exposures from participants recruited from diverse altitudes and latitudes.*

**Comment 3**: Another issue which should be considered is the influence of solar angle, as described by Sasaki, which can influence ocular exposure differently based on time of year and time of day. This was not considered in McCarty’s calculations but adds an interesting point of discussion… the latitudes nearer the equator always have higher ambient exposure, but more northern sites (or southern for the southern hemisphere) have direct ocular exposures that may be as high in early and late hours of summer days, and all day during spring and autumn (Sasaki H, Sakamoto, Schnider C et al. (2011). UV-B Exposure to the Eye Depending on Solar Altitude. Eye & contact lens. 37. 191-5.)

**Authors’ Response** – We are grateful for this pertinent comment and the useful reference. We have added this study and relevant discussion points in the manuscript to highlight this as an additional factor to be accounted for.

LINES 336-341*: Another potential factor could be the relationship between solar angle and sun exposure. As demonstrated by Sasaki et al, ocular exposure to UVR is effectually maximum at times when sun protection is less likely to be used, such as in the mid-morning, mid-afternoon; and in the fall, winter, and spring.[16] These findings merit subsequent exploration via longitudinal studies and measurements of individual levels of UV exposure to the eyes.*

LINES 429-432*: Based on the results, it is recommended future studies should try to get in-depth information on nature of headgear used and the time of day when used, duration of work on water, and ascertain individual level UV exposures from participants recruited from diverse altitudes and latitudes.*

**Comment 4:** At line 403-405, the numbers quote for exposure are not consistent with those quoted in table 1 (means versus median, perhaps). And again, the reported association of higher cataract in coastal areas followed by plain and hilly areas is out of order compared to data in table 2.
**Authors’ Response** – Thank you for pointing out the discrepancy. We have made the corrections as below

LINES 436-439: *The median duration of sun exposure in plain, hilly and coastal region was 114140, 72759 and 109889 hours respectively. A higher percentage of cataract was seen in coastal areas (42.4%) followed by plain (31.9%) and hilly areas (26.6%).*

**Reviewer #2: the paper is interesting and helps add to the literature.**

**Comment 1:** However, there are quite a few instances where the first person is used in the manuscript so I believe this should be addressed to amend to the third person.

**Authors’ Response** – We are sorry for the oversight and have now corrected the language to third person.

**Comment 2:** There also needs to be a little more about UV protection worn (hats, sunglasses, CLs) - it is mentioned briefly but not explored further.

**Authors’ Response** – Thank you for pointing this out, we did capture broadly the usage of various forms of clothing or head gear or eye wear that could provide sun protection such as whether the respondent was using facial cloth protection (veil, towel, etc), traditional headgear (pagdi, mundas, towels), umbrella, caps / hats or sunglasses/ prescription glasses (supplementary file, Question 5, Q10, Q15 of Form II). Contact lens usage was not seen in our rural population in the pilot study and accordingly we did not capture this data. We accounted for the different types of headgear use when we calculated effective sun exposure.

We have added this information in methods and results of the study and are trying to address this aspect in phase 2 of the study which is ongoing. The following sentences have been added in the manuscript.

LINES 128-130*: The sun-protection factors related to the degrees of ocular protection applied in the calculation are 0.53 for facial cloth protection (veil, towel, etc), traditional headgear (pagdi, mundas, towels), umbrella, and caps / hats, and 0.21 for sunglasses.[6]*

LINES 201-204: *Among the respondents, 36.7% were not using any protective headgear against sunlight when outdoors during 9AM to 5PM. Most of the study population was using either veil/dupatta or saree or some form of traditional headgear (32.0%). Umbrella (2.46%), caps (1.54%) and sunglasses (1.06%) were used rarely.*

LINES 417-423*: The study did not capture detailed information on the designs of headgear, or the particular eye wear such as type of sunglasses, spectacles etc. None of the patients reported use of contact lenses, which was not surprising given that the study was done in rural Indian populations. It has been reported in literature that different types of headgear and sunglasses may accord widely varying levels of protection depending on the geometry, wearing position, head positions and exposure conditions.[36,37]*

LINES 432-433*: Finally, models of levels of UV protection accorded by different types of headgear and eye-wear used in India should be developed.*

6. PLOS authors have the option to publish the peer review history of their article ([what does this mean?](https://journals.plos.org/plosone/s/editorial-and-peer-review-process#loc-peer-review-history)). If published, this will include your full peer review and any attached files.

If you choose “no”, your identity will remain anonymous but your review may still be made public.

**Do you want your identity to be public for this peer review?** For information about this choice, including consent withdrawal, please see our [Privacy Policy](https://www.plos.org/privacy-policy).

Reviewer #1: Yes: Cristina Schnider, OD, MSc, MBA, FAAO

Reviewer #2: No

[NOTE: If reviewer comments were submitted as an attachment file, they will be attached to this email and accessible via the submission site. Please log into your account, locate the manuscript record, and check for the action link "View Attachments". If this link does not appear, there are no attachment files to be viewed.]


While revising your submission, please upload your figure files to the Preflight Analysis and Conversion Engine (PACE) digital diagnostic tool, <https://pacev2.apexcovantage.com/>. PACE helps ensure that figures meet PLOS requirements. To use PACE, you must first register as a user. Registration is free. Then, login and navigate to the UPLOAD tab, where you will find detailed instructions on how to use the tool. If you encounter any issues or have any questions when using PACE, please email us at [figures@plos.org](mailto:figures@plos.org). Please note that Supporting Information files do not need this step.
